# Regulation of mitophagy by the NSL complex underlies genetic risk for Parkinson’s disease at 16q11.2 and MAPT H1 loci

**DOI:** 10.1093/brain/awac325

**Published:** 2022-09-08

**Authors:** Marc P M Soutar, Daniela Melandri, Benjamin O’Callaghan, Emily Annuario, Amy E Monaghan, Natalie J Welsh, Karishma D’Sa, Sebastian Guelfi, David Zhang, Alan Pittman, Daniah Trabzuni, Anouk H A Verboven, Kylie S Pan, Demis A Kia, Magda Bictash, Sonia Gandhi, Henry Houlden, Mark R Cookson, Nael Nadif Kasri, Nicholas W Wood, Andrew B Singleton, John Hardy, Paul J Whiting, Cornelis Blauwendraat, Alexander J Whitworth, Claudia Manzoni, Mina Ryten, Patrick A Lewis, Hélène Plun-Favreau

**Affiliations:** Department of Neurodegenerative Disease, UCL Queen Square Institute of Neurology, London, UK; Department of Neurodegenerative Disease, UCL Queen Square Institute of Neurology, London, UK; Department of Neurodegenerative Disease, UCL Queen Square Institute of Neurology, London, UK; Aligning Science Across Parkinson’s (ASAP) Collaborative Research Network, Chevy Chase, MD, USA; Department of Basic and Clinical Neuroscience, King’s College, London, UK; UCL Alzheimer’s Research UK, Drug Discovery Institute, London, UK; UCL Dementia Research Institute, London, UK; MRC Mitochondrial Biology Unit, University of Cambridge, Cambridge, UK; Aligning Science Across Parkinson’s (ASAP) Collaborative Research Network, Chevy Chase, MD, USA; Francis Crick Institute, London, UK; NIHR Great Ormond Street Hospital Biomedical Research Centre, University College London, London, UK; Department of Neurodegenerative Disease, UCL Queen Square Institute of Neurology, London, UK; Aligning Science Across Parkinson’s (ASAP) Collaborative Research Network, Chevy Chase, MD, USA; Genetics Research Centre, Molecular and Clinical Sciences, St Georges University, London, UK; Department of Neurodegenerative Disease, UCL Queen Square Institute of Neurology, London, UK; Department of Human Genetics, Radboudumc, Donders Institute for Brain, Cognition and Behaviour, 6500 HB Nijmegen, The Netherlands; Department of Cognitive Neuroscience, Radboudumc, Donders Institute for Brain, Cognition and Behaviour, 6500 HB Nijmegen, The Netherlands; Department of Neurodegenerative Disease, UCL Queen Square Institute of Neurology, London, UK; Aligning Science Across Parkinson’s (ASAP) Collaborative Research Network, Chevy Chase, MD, USA; Department of Clinical and Movement Neuroscience, UCL Queen Square Institute of Neurology, London, UK; UCL Alzheimer’s Research UK, Drug Discovery Institute, London, UK; UCL Dementia Research Institute, London, UK; Aligning Science Across Parkinson’s (ASAP) Collaborative Research Network, Chevy Chase, MD, USA; Francis Crick Institute, London, UK; Department of Clinical and Movement Neuroscience, UCL Queen Square Institute of Neurology, London, UK; Department of Neuromuscular Disease, UCL Queen Square Institute of Neurology, London, UK; Laboratory of Neurogenetics, National Institute on Aging, National Institutes of Health, Bethesda, MD, USA; Department of Human Genetics, Radboudumc, Donders Institute for Brain, Cognition and Behaviour, 6500 HB Nijmegen, The Netherlands; Department of Cognitive Neuroscience, Radboudumc, Donders Institute for Brain, Cognition and Behaviour, 6500 HB Nijmegen, The Netherlands; Aligning Science Across Parkinson’s (ASAP) Collaborative Research Network, Chevy Chase, MD, USA; Department of Clinical and Movement Neuroscience, UCL Queen Square Institute of Neurology, London, UK; Laboratory of Neurogenetics, National Institute on Aging, National Institutes of Health, Bethesda, MD, USA; Center for Alzheimer's and Related Dementias, National Institute on Aging and National Institute of Neurological Disorders and Stroke, National Institutes of Health, Bethesda, MD, USA; Department of Neurodegenerative Disease, UCL Queen Square Institute of Neurology, London, UK; Aligning Science Across Parkinson’s (ASAP) Collaborative Research Network, Chevy Chase, MD, USA; UCL Dementia Research Institute, London, UK; UCL Alzheimer’s Research UK, Drug Discovery Institute, London, UK; UCL Dementia Research Institute, London, UK; Laboratory of Neurogenetics, National Institute on Aging, National Institutes of Health, Bethesda, MD, USA; Center for Alzheimer's and Related Dementias, National Institute on Aging and National Institute of Neurological Disorders and Stroke, National Institutes of Health, Bethesda, MD, USA; MRC Mitochondrial Biology Unit, University of Cambridge, Cambridge, UK; Department of Pharmacology, UCL School of Pharmacy, London, UK; Aligning Science Across Parkinson’s (ASAP) Collaborative Research Network, Chevy Chase, MD, USA; NIHR Great Ormond Street Hospital Biomedical Research Centre, University College London, London, UK; Department of Genetics and Genomic Medicine, Great Ormond Street Institute of Child Health, University College London, London, UK; Department of Neurodegenerative Disease, UCL Queen Square Institute of Neurology, London, UK; Aligning Science Across Parkinson’s (ASAP) Collaborative Research Network, Chevy Chase, MD, USA; Department of Comparative Biomedical Sciences, Royal Veterinary College, LondonUK; Department of Neurodegenerative Disease, UCL Queen Square Institute of Neurology, London, UK; Aligning Science Across Parkinson’s (ASAP) Collaborative Research Network, Chevy Chase, MD, USA

**Keywords:** GWAS, KANSL1, KAT8, mitophagy, Parkinson’s disease

## Abstract

Parkinson’s disease is a common incurable neurodegenerative disease. The identification of genetic variants via genome-wide association studies has considerably advanced our understanding of the Parkinson’s disease genetic risk. Understanding the functional significance of the risk loci is now a critical step towards translating these genetic advances into an enhanced biological understanding of the disease. Impaired mitophagy is a key causative pathway in familial Parkinson’s disease, but its relevance to idiopathic Parkinson’s disease is unclear. We used a mitophagy screening assay to evaluate the functional significance of risk genes identified through genome-wide association studies. We identified two new regulators of PINK1-dependent mitophagy initiation, KAT8 and KANSL1, previously shown to modulate lysine acetylation. These findings suggest PINK1-mitophagy is a contributing factor to idiopathic Parkinson’s disease. *KANSL1* is located on chromosome 17q21 where the risk associated gene has long been considered to be *MAPT*. While our data do not exclude a possible association between the *MAPT* gene and Parkinson’s disease, they provide strong evidence that *KANSL1* plays a crucial role in the disease. Finally, these results enrich our understanding of physiological events regulating mitophagy and establish a novel pathway for drug targeting in neurodegeneration.

## Teaser

Soutar *et al.* show that two Parkinson's disease candidate genes, *KANSL1* and *KAT8*, are regulators of PINK1-dependent mitophagy in neurons. The findings suggest that impairments in PINK1-mediated mitochondrial quality control may be a contributing factor to idiopathic Parkinson’s disease.

**See Ganley (https://doi.org/10.1093/brain/awac405) for a scientific commentary on this article.**

## Introduction

Parkinson’s disease is the most common movement disorder in old age and afflicts >125 000 in the UK.^1^ Temporary symptomatic relief remains the cornerstone of current treatments, with no disease-modifying therapies yet available.^2^ Until recently, the genetic basis for Parkinson’s disease was limited to family-based linkage studies, favouring the identification of rare Mendelian genes of high penetrance and effect. However, genome-wide association studies (GWAS) have identified large numbers of common genetic variants linked to increased risk of developing the disease.^3,4^ While these genetic discoveries have led to a rapid improvement in our understanding of the genetic architecture of Parkinson’s disease,^5^ they have resulted in two major challenges for the research community. First, conclusively identifying the causal gene(s) for a given risk locus, and second, dissecting their contribution to disease pathogenesis. Addressing these challenges is critical for moving beyond genetic insights to developing new disease-modifying strategies for Parkinson’s disease.

Previous functional analyses of *PINK1* (PTEN-induced kinase 1) and *PRKN*, two genes associated with autosomal recessive Parkinson’s disease, have highlighted the selective degradation of damaged mitochondria (mitophagy) as a key contributor to disease pathogenesis. In mammalian cells, the mitochondrial kinase PINK1 selectively accumulates at the surface of damaged mitochondria, where it phosphorylates ubiquitin, leading to the recruitment and phosphorylation of the E3 ubiquitin ligase Parkin. The recruitment of autophagy receptors leads to the engulfment of damaged mitochondria in autophagosomes, and ultimately fusion with lysosomes^6–11^. It has subsequently become clear that other Parkinson’s disease-associated Mendelian genes, such as *FBXO7*, *DJ-1* and *VPS35*,^12^ are implicated in the regulation of PINK1-mediated mitochondrial quality control. On the basis of these data, we hypothesized that Parkinson’s disease GWAS candidate genes may also be involved in this process, providing a mechanistic link between these genes and the aetiology of idiopathic Parkinson’s disease. To test that hypothesis, we used functional genomics to prioritize candidate genes at the Parkinson’s disease GWAS loci, and we developed a phenotypic high-content screening assay as a tool to identify genes that regulate PINK1-dependent mitophagy initiation and, as such, are likely to be genes that influence the risk of developing Parkinson’s disease.

In this study, we show that Parkinson’s disease GWAS risk gene candidates KAT8 (lysine acetyltransferase 8) and KANSL1 (KAT8 Regulatory NSL Complex Subunit 1), which are both part of the non-specific lethal (NSL) complex, are new and important regulators of PINK1-mediated mitochondrial quality control. KAT8 is a histone acetyltransferase belonging to the MYST family that represents the major catalytic constituent of two distinct protein complexes: the male-specific lethal and NSL complex.^13,14^ Alongside KAT8, the NSL complex consists of eight additional proteins: Host Cell Factor C1, KANSL1, KANSL2, KANSL3, Microspherule Protein 1 (MCRS1), O-linked *N*-acetylglucosaminyltransferase (OGT), PHD Finger Protein 20 (PHF20)- and WD Repeat Domain 5 (WDR5).^13^ KAT8 is the catalytically active acetytransferase of the NSL complex, responsible for the deposition of acetylation modifications on lysine 16 of histone H4 (H4K16ac) facilitating chromatin decompaction making it more permissible for transcriptional machinery and target gene expression,^15^ in addition to pro-transcriptional H4K5ac and H4K8ac.^16^ The NSL complex regulates the expression of genes involved in a multitude of crucial biological processes including proliferation, metabolism, transcription, DNA replication and autophagy.^15–23^ In addition to its canonical role in the nucleus, components of the NSL complex have also been suggested to partially localize to the mitochondria (KAT8, KANSL1, KANSL2, KANSL3 and MCRS1) where they regulate mtDNA transcription and mitochondrial oxidative metabolism.^24^

These findings suggest that mitophagy contributes to idiopathic Parkinson’s disease and provides a proof of principle for functional screening approaches to identify causative genes in GWAS loci. Finally, these results indicate lysine acetylation as a potential new avenue for mitophagy modulation and therapeutic intervention.

## Materials and methods

The following methods are available on protocols.io: cell-based *in vitro* assays, dx.doi.org/10.17504/protocols.io.5jyl89648v2w/v1; bioinformatic prioritization and hit validation, dx.doi.org/10.17504/protocols.io.3byl4br2zvo5/v1; *Drosophila* stocks, husbandry, locomotor and lifespan assays and immunohistochemistry and sample preparation, dx.doi.org/10.17504/protocols.io.eq2lyn1dqvx9/v1.

### Reagents

Oligomycin (mitochondrial complex V inhibitor) was purchased from Cayman Chemicals (11341) and from Sigma-Aldrich (O4876), and antimycin A (mitochondrial complex III inhibitor) were purchased from Sigma-Aldrich (A8674). All small-interfering RNAs (siRNAs) were purchased as pre-designed siGENOME SMARTpools from Dharmacon: non-targeting (D-001206-13), PINK1 (M-004030-02), PLK1 (L-003290-00), KIF-11 (L-003317-00), KAT8 (M-014800-00), KANSL1 (M-031748-00), KANSL2 (M-020816-01), KANSL3 (M-016928-01), Host Cell Factor C1 (M-019953-01), MCRS1 (M-018557-00), OGT (M-019111-00) and PHF20 (M-015234-02), WDR5 (M-013383-01). The following antibodies were used for immunocytochemistry: mouse anti-TOM20 (Santa Cruz, sc-17764, RRID:AB_628381, 1:1000), rabbit anti-phospho-ubiquitin (Ser65) (Cell Signaling, 37642, 1:1000), rabbit anti-phospho-Parkin (Ser65) (Abcam/Michael J. Fox Foundation, MJF17, 1:250), rabbit anti-FLAG (Sigma-Aldrich, F7425, RRID:AB_439687, 1:500), AlexaFluor 488 goat anti-rabbit (Invitrogen, A11008, RRID:AB_143165, 1:2000), AlexaFluor 568 goat anti-mouse (Invitrogen, A11004, RRID:AB_2534072, 1:2000), AlexaFluor 488 donkey anti-rabbit (Invitrogen, A21206, RRID:AB_2535792, 1:2000), AlexaFluor 647 donkey anti-mouse (Invitrogen, A31571, RRID:AB_162542, 1:2000). The following antibodies were used for immunoblotting: mouse anti-TIM23 (BD Biosciences, 611223, RRID:AB_398755, 1:1000), rabbit anti-TOM20 (Santa Cruz, sc-11415, RRID:AB_2207533, 1:1000), rabbit anti-phospho-ubiquitin (Ser65) (Merck Millipore, ABS1513-I, RRID:AB_2858191, 1:1000; and Cell Signaling, 37642, 1:1000), mouse anti-GAPDH (Abcam, ab110305, RRID:AB_10861081, 1:10 000), rabbit anti-KAT8 (Abcam, ab200660, RRID:AB_2891127, 1:1000), rabbit anti-total-Tau (DAKO, A0024, RRID:AB_10013724, 1:10 000), mouse anti-V5 tag (Invitrogen, R960-25, RRID:AB_2556564, 1:1000), rabbit anti-KANSL1 (Sigma-Aldrich, HPA006874, RRID:AB_1852393, 1:500), rabbit anti-pParkin(Ser65) (Parkin Overexpressing) (Abcam/Michael J. Fox Foundation, MJF17, 1:1000), mouse anti-FLAG M2 (Sigma-Aldrich, F3165, RRID:AB_259529, 1:1000), sheep anti-pRab8A(Ser111) (Phosphorylated Rab8a (Serine111)) (MRC Protein Phosphorylation and Ubiquitylation Unit, University of Dundee, 1 µg/ml preblocked with 10 µg/ml non-phosphorylated peptide^25^), rabbit anti-total Rab8 (Cell Signaling, 6975, RRID:AB_10827742, 1:1000), IRDye 680LT donkey anti-mouse (LI-COR Biosciences, 925-68022, RRID:AB_2814906, 1:20 000), IRDye 800CW donkey anti-rabbit (LI-COR Biosciences, 925-32213, RRID:AB_2715510, 1:20 000), IRDye 800CW Donkey anti-Goat (LI-COR Biosciences, 926-32214, RRID:AB_621846, 1:20 000), IRDye^®^ 680RD Goat anti-Rabbit (LI-COR Biosciences, 926-68071, RRID:AB_10956166, 1:20 000). The generation of rabbit monoclonal anti-PINK1 antibody has been described elsewhere,^26^ and is available on reasonable request to the corresponding author.

### Selection of genes for high-content screening

Candidates for high-content screening were selected on the basis of (i) weighted protein–protein interaction network analysis (WPPINA); (ii) complex prioritization; and (iii) coloc analysis. WPPINA analysis is reported in Ferrari *et al.*,^27^ where the 2014 Parkinson’s disease GWAS^28^ was analysed; candidate genes where selected among those prioritized and with a linkage disequilibrium (LD) *r*^2^ ≥ 0.8. The same pipeline has then been additionally applied to the 2017 Parkinson’s disease GWAS^3^ to update the list of candidate genes. Briefly, a protein–protein interaction network has been created based on the Mendelian genes for Parkinson’s disease (seeds) using data from databases within the International Molecular Exchange consortium. The network has been topologically analysed to extract the core network (i.e. the most interconnected part of the network). The core network contains the proteins/genes that can connect >60% of the initial seeds and are therefore considered relevant for sustaining communal processes and pathways, shared by the seeds. These processes have been evaluated by Gene Ontology Biological Processes enrichment analysis. The top single nucleotide polymorphisms (SNPs) of the 2017 Parkinson’s disease GWAS have been used to extract open reading frames (ORFs) in *cis*-haplotypes defined by LD *r*^2^ ≥ 0.8 (analysis performed in October 2017). These ORFs have been matched to the core network to identify overlapping proteins/genes in relevant/shared pathways. Results of complex prioritization (neurocentric prioritization strategy) were gathered from Change *et al*.,^3^ where this strategy was applied to the 2017 Parkinson’s disease GWAS. The coloc analysis was performed as reported by Kia *et al*.^29^; posterior probabilities for the hypothesis that both traits, the regulation of expression of a given gene and the risk for Parkinson’s disease share a causal variant (PPH4), were calculated for each gene and genes with PPH4 ≥ 0.75 were considered to have strong evidence for colocalization. Summary statistics were obtained from the most recent Parkinson’s disease GWAS^4^ and were used for regional association plotting using LocusZoom.^30^ With the exception of *PM20D1* all genes are expressed in SHSY5Y cells according to publicly available expression data deposited in the Human Protein Atlas (proteinatlas.org)^31^ and EBI Expression Atlas (http://www.ebi.ac.uk/gxa).^32^

### Cell culture and siRNA transfection

Parkin overexpressing (POE) SHSY5Y cells are a kind gift from H. Ardley^33^ and the mt-Keima POE SHSY5Y cells were a kind gift from C. Luft.^34^ PINK1-HA overexpressing SHSY5Y cells were a kind gift from E. Deas.^35^ Wild-type (WT) SHSY5Y (RRID:CVCL_0019) and H4 (RRID:CVCL_1239) cells were sourced from American Type Culture Collection (ATCC, RRID:SCR_00167). Lenti-X293T human embryonic kidney (HEK) cells were sourced from Takara Bio (632180, RRID: CVCL_4401). Cells were cultured in Dulbecco’s Modified Eagle Medium (DMEM, Gibco, 11995-065) supplemented with 10% heat-inactivated foetal bovine serum (FBS, Gibco) in a humidified chamber at 37°C with 5% CO_2_. For siRNA transfection, cells were transfected using DharmaFECT1 transfection reagent (Dharmacon, T-2001-03) according to the manufacturer’s instructions (for concentrations of siRNA, see the following sections). Whole genome-sequencing shows SHSY5Ys are of the H1/H1 haplotype (data not shown).

### KANSL1 iNeuron culture and differentiation

Isogenic human induced pluripotent stem cell (hiPSC) lines with/without a heterozygous loss-of-function (LoF) frameshift mutation in Exon2 of the KANSL1 gene (c.531insT), which have also being stable transduced with transgenes permitting doxycycline-inducible overexpression of murine Ngn2 were a kind gift from the laboratory of N. Nadif Kasri and have been published elsewhere.^22^ Whole genome-sequencing shows the parental line is of the H1/H2 haplotype, with Sanger sequencing of KANSL1 cDNA revealing the LoF frameshift mutation in the KANSL1^+/−^ line is on the H2 haplotype (i.e. H1/-) (data not shown). hiPSCs were cultured on Geltrex (Thermo Fisher) coated culture dishes in mTeSR1 (StemCell Technologies) and maintained in a humidified 37°C incubator, 5% CO_2_.

Isogenic KANSL1^+/+^ and KANSL1^+/−^hiPSCs were differentiated into excitatory cortical neurons by doxycycline induced overexpression of murine Ngn2 by adapting recently published protocols.^22,36^ On d0, hiPSCs were first dissociated into a single cell suspension using accutase (Sigma) before plating in induction medium consisting of DMEM/F12 supplemented with 1× Glutamax, 1× non-essential amino acids, 1× N2-supplement (all Thermo Fisher), 4 µg/ml doxycyxline (Sigma). Induction media was additionally supplemented with 10 µM Y-27632 Rho Kinase inhibitor (ROCKi, Peprotech) during initial seeding. Next, 7.5 × 10^5^ cells were seeded onto Geltrex-coated six-well plates and 24 h later (d1) and 48 h later (d2) a full medium change was performed with freshly prepared induction media without Y-27632 ROCKi. On d3, a full medium change was performed with freshly prepared N2-B27 media consisting of a 1:1 mixture of DMEM/F12:neurobasal supplemented with 0.5× N2-supplement, 0.5× B27 supplement, 0.5× non-essential amino acids, 0.5× Glutamax, 45 µM 2-Mercaptoethanol (all Thermo Fisher), 2.7 µg/ml insulin (Sigma). N2-B27 media was additionally supplemented with 2 µM cytosine β-D-arabinofuranoside (Sigma) on d3. A half media change with N2-B27 lacking cytosine β-D-arabinofuranoside was performed every 3–4 days thereafter. A half media change with N2-B27 was performed on d16 with cells collected for experimental assays 24 h later on d17.

### CRISPRi-i3N iNeuron culture and differentiation

A hiPSC line stably transduced with transgenes permitting doxycycline-inducible overexpression of murine Ngn2 at the AAVS1 safe-harbour locus, and stably transduced with constitutively expressed enzymatically dead Cas9-KRAB transcriptional repressor fusion protein at the CLYBL promoter safe-harbour locus (CRISPRi-i3N hiPSCs) was a kind gift from the laboratories of M.E. Ward and M. Kampmann, and have been published elsewhere.^37,38^ hiPSCs were cultured as outlined previously for KANSL1 hiPSCs.

Clustered regularly interspaced short palindromic repeats with interference- (CRISPRi-)i3N hiPSCs were first transduced with lentiviral particles encoding a mCherry-reporter and single-guide RNA (sgRNA) sequences targeting the promoter regions of *KANSL1*, *KAT8* or *PINK1*, or a non-targeting sgRNA control (see Supplementary Table 1 for sgRNA sequences, and below for lentiviral production). Transduced cells were then differentiated into excitatory cortical neurons by doxycycline induced overexpression of Ngn2 by adapting recently published protocols.^37,38^

On d-1 CRISPRi-i3N hiPSCs were first dissociated into a single cell suspension using accutase and reverse transduced with sgRNA lentiviral supernatant in mTeSR1 supplemented with 5 µg/ml polybrene and 10 µM Y-27632 ROCKi. Next, 4.5 × 10^5^ cells were seeded onto Geltrex-coated six-well plates and 24 h later (d0) media was changed to induction media (composition outlined previously for KANSL1 iNeurons). Then 24 h (d1) and 48 h later (d2), a full medium change was performed with freshly prepared induction media. On d3, differentiating iNeurons were dissociated into a single cell suspension using accutase (Sigma) and seeded in N2-B27 media (composition outlined previously for KANSL1 iNeurons) into Geltrex-coated 96-well CellCarrier Ultra plates for immunofluorescence (IF) (3 × 10^4^ cells per well) and 12-well plates (5 × 10^5^ cells per well) for biochemistry purposes. A half media change with N2-B27 was performed the following day (d4) and every 3–4 days thereafter. A half media change with N2-B27 was performed on d16 with cells collected for experimental assays 24 h later on d17.

### Lentiviral particle generation

Seventy to ninety percent confluent Lenti-X 293T HEK cells cultured in DMEM 10% FBS media were transfected with pMD2.G and pCMVR8.74 alongside appropriate delivery plasmids: pLV[Exp]-U6 > sgRNA-hPGK > mApple (Vectorbuilder) plasmids (for sgRNA), empty pLVX-EF1α-IRES-Puro (Clontech, Takara Bio, 631988), V5-KANSL1 pLVX-EF1α-IRES-Puro or V5-KAT8 pLV[Exp]-EF1α-IRES-Puro at a 1:1:2 molar mass ratio using Lipofectamine 3000 (Invitrogen). The next day, a full media change was performed with mTeSR1 (for sgRNA lentivirus) or DMEM 10% FBS media and cells cultured for 24 h. The lentivirus containing mTeSR1/DMEM 10% FBS was collected and diluted 1:2 with fresh mTeSR1 or 10% FBS before filtering through 0.44 µm PES filters. pMD2.G (Addgene plasmid no. 12259, RRID:Addgene_12259) and pCMVR8.74 (Addgene plasmid #22036, RRID:Addgene_22036) were gifts from Didier Trono. KANSL1 cDNA (ENST00000432791.7) with N-terminal V5 tag was cloned into the pLVX-EF1α-IRES-Puro plasmid using SpeI and NotI restriction sites (doi:10.5281/zenodo.6903553). See Supplementary Table 1 for sgRNA plasmids and V5-KAT8 pLV[Exp]-EF1α-IRES-Puro plasmids (Vectorbuilder).

### Allele-specific expressions

Sites of allele-specific expression (ASE) were identified as described by Guelfi and colleagues^39^ by mapping RNA-sequencing (RNA-seq) data to personalized genomes, an approach specifically chosen because it aims to minimize the impact of mapping biases. RNA-seq data generated from 49 putamen and 35 substantia nigra tissue samples from the UK Brain Expression Consortium was used for this analysis^39^ and can be accessed through European Genome-phenome Archive numbers EGAS00001002113 and EGAS00001003065. All samples were obtained from neuropathologically normal individuals of European descent and sites with >15 reads in a sample were tested for ASE. Only sites present in non-overlapping genes were considered and data from both the tissues were considered together to increase power. Sites with minimum false discovery rate <5% across samples were marked as ASE sites. Plots were generated using Gviz3 (https://bioconductor.org/packages/Gviz/), with gene and transcript details obtained from Ensembl v92 (http://www.ensembl.org/, RRID:SCR_002344).

### High-content siRNA screen

#### Cell plating and siRNA transfection

siRNA was dispensed into Geltrex-coated 96-well CellCarrier Ultra plates (Perkin Elmer) at a final concentration of 30 nM using the Echo 555 acoustic liquid handler (Labcyte). For each well, 25 µl of DMEM containing 4.8 µl/ml of DharmaFECT1 transfection reagent was added and incubated for 30 min before POE SHSY5Y cells were seeded using the CyBio SELMA (Analytik Jena) at 15 000 cells per well, 100 µl per well in DMEM + 10% FBS. Cells were incubated for 72 h before treatment with 10 µM oligomycin/10 µM antimycin for 3 h to induce mitophagy. Positive hits from the screen were validated further, however, without determining functional siRNA knockdown (KD) of all gene targets in the screens, none of the negative hits can be formally excluded as regulators of PINK1-dependent mitophagy initiation.

#### IF and image capture and analysis

Cells were fixed with 4% paraformaldehyde (Sigma-Aldrich, F8775), then blocked and permeabilized with 10% FBS, 0.25% Triton X-100 in phosphate buffered saline (PBS) for 1 h, before immunostaining with pUb(Ser65) [Phosphorylated Ubiquitin (Serine 65)] and TOM20 primary antibodies (in 10% FBS/PBS) for 2 h at room temperature. After 3× PBS washes, AlexaFluor 568 anti-mouse and 488 anti-rabbit secondary antibodies and Hoechst 33342 (Thermo Scientific, 62249) were added (in 10% FBS/PBS, 1:2000 dilution for all) and incubated for 1 h at room temperature. Following a final round of 3× PBS washes, plates were imaged using the Opera Phenix (Perkin Elmer). 5× fields of view and 4× 1 µm *Z* planes were acquired per well, using the ×40 water objective with a numerical aperture (NA) of 1.1. Images were analysed in an automated way using the Columbus 2.8 analysis system (Perkin Elmer, https://www.perkinelmer.com/en-ca/product/image-data-storage-and-analysis-system-columbus) to measure the integrated intensity of pUb(Ser65) within the whole cell. First of all, the image was loaded as a maximum projection, before being segmented to identify the nuclei using the Hoechst 33342 channel (method B). The cytoplasm was then identified using the ‘Find Cytoplasm’ building block (method B) on the sum of the Hoechst and Alexa 568 channels. pUb(Ser65) was identified as spots (method B) on the Alexa 488 channel, before measuring their integrated intensity.

#### Screen quality control, data processing and candidate selection

Screen plates were quality controlled based on the efficacy of the PINK1 siRNA control and O/A treatment window (minimum 3-fold). Data were checked for edge effects using Dotmatics Vortex visualization software. Raw data was quality controlled using robust *Z* prime >0.5. Data were processed using Python (http://www.python.org/, RRID:SCR_008394) for *Z* score calculation before visualization in Dotmatics Vortex. Candidates were considered a hit where *Z* score was ≥2 or ≤−2, and where replication of efficacy was seen both within and across plates.

#### siRNA libraries

The siRNA libraries were purchased from Dharmacon as an ON-TARGETplus SMARTpool Cherry-pick siRNA library, 0.25 nmol in a 384-well plate. siRNAs were resuspended in RNase-free water for a final concentration of 20 µM. SCR (Scrambled), PINK1 and PLK1 or KIF11 controls were added to the 384-well plate at a concentration of 20 µM before dispensing into barcoded assay-ready plates.

### Mitochondrial enrichment and western blotting

POE SHSY5Y and H4 cells were transfected with 100 nM siRNA and incubated for 72 h. KANSL1 iNeurons were cultured as detailed previously. Whole-cell lysates were used from POE SHSY5Y cells, H4 cells and KANSL1 iNeurons. For some experiments, POE SHSY5Y lysates were first fractionated into cytoplasmic and mitochondria-enriched preparations to facilitate detection of mitochondrial localized proteins of interest. Samples were run on sodium dodecyl sulphate–polyacrylamide gel electrophoresis before immunoblot (IB) with the Odyssey^®^ CLx Imager (LI-COR Biosciences). Mitochondrial enrichment and western blotting protocols were described previously.^26^

### siRNA KD rescue

POE SHSY5Ys were transfected with 25 nM siRNA (d0) and incubated for 48 h. siRNA KD cells were then transduced with lentivirus in the presence of 10 µg/ml polybrene (d2), full media change performed the following day (d3) and collected 4 days post-siRNA transfection (and 2 days post-lentivirus transduction) (d4).

### pRab8A(Ser111) measurements

SHSY5Y cells stably overexpressing PINK1-HA were transfected with 100 nM siRNA and incubated for 72 h. Then 200 µg of protein (whole-cell lysate) were immunoprecipitated with Protein A Dynabeads™ (Invitrogen) prebound with 0.5 µg of rabbit anti-total-Rab8 antibody (Cell Signaling, 6975) at 4°C overnight. Samples were eluted from the beads by heating at 95°C in 2× LDS supplemented with 50 mM DTT for 5 min.

### Immunofluorescence

SHSY5Y cells were reverse transfected with 50 nM siRNA in 96-well CellCarrier Ultra plates according to the manufacturer’s instructions and incubated for 72 h. CRISPRi-i3N iNeurons were cultured as described before. Cells were then treated, fixed and stained as per the screening protocol detailed previously (for treatment concentrations and times, see corresponding figures). For visualization purposes, brightness and contrast settings were selected on the SCR (siRNA KD SHSY5Y) or no transduction (CRISPRi-i3N iNeurons) controls and applied to all other images. Images are presented as maximum projections of the channels for one field of view.

### Real-time qualitative PCR

Total RNA was extracted from cells using the Monarch Total RNA Miniprep Kit (New England Bioscience) with inclusion of the optional on-column DNAse treatment and quantified using a NanoDrop One Spectrophotometer (Thermo Fisher Scientific). RNA was then reverse transcribed in a 10 µl reaction with 2.5 U/µl SuperScript IV reverse transcriptase with 2.5 µM random hexamers, 0.5 mM dNTPs, 5 mM DTT and 2 U/µl RNAseOUT (all Invitrogen). Equal amounts of RNA were reverse transcribed for all samples of a single experiment, with 500 ng of RNA in a 10 µl reverse transcription reaction being the most common. The cDNA product was then diluted such that 500 ng of reverse transcribed RNA would be in a 600 µl final volume (i.e. 0.83 ng/µl). Next, 4 µl (i.e. 3.33 ng) of the diluted cDNA was then subjected to real-time quantitative PCR (qPCR) using 1× Fast SYBR^™^ Green Master Mix (Applied Biosystems) and 500 nM gene specific primer pairs (Supplementary Table 2) on a QuantStudio^™^ 7 Flex Real-Time PCR System (Applied Biosystems). At least 2× technical replicates were performed for each sample and gene target combination, and a real-time^−^ control for all samples and gene target combinations was performed alongside in most assays, with rare exceptions due to test well number limitations. Relative mRNA expression levels were calculated using the 2^−ΔΔCt^ method and *RPL18A* (SHSY5Y and H4 cells) or *UBC* (iNeurons) as the house-keeping gene.

### Mitophagy measurement using the mt-Keima reporter

Stable mt-Keima expressing POE SHSY5Y cells were reverse transfected with 50 nM siRNA in 96-well CellCarrier Ultra plates according to the manufacturer’s instructions and incubated for 72 h. For the assay, the cell medium was replaced with phenol-free DMEM + 10% FBS containing Hoechst 33342 (1:10000) and either DMSO or 1 µM oligomycin/1 µM antimycin to induce mitophagy. Cells were immediately imaged on the Opera Phenix (PerkinElmer) at 37 °C with 5% CO_2_, acquiring 15× single plane fields of view, using the ×63 water objective, NA1.15. The following excitation wavelengths and emission filters were used: cytoplasmic Keima: 488 nm, 650–760 nm; lysosomal Keima: 561 nm, 570–630 nm; Hoechst 33342: 375 nm, 435–480 nm. Images were analysed in an automated way using the Columbus 2.8 analysis system (Perkin Elmer) to measure the mitophagy index. Cells were identified using the nuclear signal of the Hoechst 33342 channel, before segmenting and measuring the area of the cytoplasmic and lysosomal mt-Keima. The mitophagy index was calculated as the ratio between the total area of lysosomal mitochondria and the total area of mt-Keima (sum of the cytoplasmic and lysosomal mt-Keima areas) per well.

### *Drosophila* stocks and husbandry

Flies were raised under standard conditions in a humidified, temperature-controlled incubator with a 12h:12 h light:dark cycle at 25°C, on food consisting of agar, cornmeal, molasses, propionic acid and yeast. The following strains were obtained from the Bloomington *Drosophila* Stock Center (RRID:SCR_006457): *mof* RNAi lines, P{TRiP.JF01701} (RRID:BDSC_31401); and P{TRiP.HMS00537} (RRID:BDSC_58281); *nsl1* RNAi lines, P{TRiP.HMJ22458} (RRID:BDSC_58328); the pan-neuronal *nSyb-GAL4* driver (RRID:BDSC_51941); and dopaminergic neuron driver (TH-GAL4; RRID:BDSC_8848); and control (*lacZ*) RNAi P{GD936}v51446 (RRID:FlyBase_FBst0469426) from the Vienna *Drosophila* Resource Center (RRID:SCR_013805). All experiments were conducted using male flies.

#### Locomotor and lifespan assays

The startle induced negative geotaxis (climbing) assay was performed using a counter-current apparatus. Briefly, 20–23 males were placed into the first chamber, tapped to the bottom and given 10 s to climb a 10 cm distance. This procedure was repeated five times (five chambers), and the number of flies that has remained into each chamber counted. The weighted performance of several group of flies for each genotype was normalized to the maximum possible score and expressed as Climbing index.^40^

For lifespan experiments, flies were grown under identical conditions at low-density. Progeny were collected under very light anaesthesia and kept in tubes of ∼20 males each, ∼50–100 in total. Flies were transferred every 2–3 days to fresh medium and the number of dead flies recorded. Percentage survival was calculated at the end of the experiment after correcting for any accidental loss.

#### Immunohistochemistry and sample preparation

*Drosophila* brains were dissected from aged flies and immunostained as described previously.^41^ Adult brains were dissected in PBS and fixed in 4% formaldehyde for 30 min on ice, permeabilized in 0.3% Triton X-100 times 20 min, and blocked with 0.3% Triton X-100 plus 4% goat serum in PBS for 4 h at room temperature. Tissues were incubated with anti-tyrosine hydroxylase (Immunostar Inc. no. 22491, RRID:AB_572268), diluted in 0.3% Triton X-100 plus 4% goat serum in PBS for 72 h at 4°C, then rinsed three times for 20 min with 0.3% Triton X-100 in PBS and incubated with the appropriate fluorescent secondary antibodies overnight at 4°C. The tissues were washed twice in PBS and mounted on slides using Prolong Diamond Antifade mounting medium (Thermo Fisher). Brains were imaged with a Zeiss LMS 880 confocal. Tyrosine hydroxylase-positive neurons were counted under blinded conditions.

### Statistical analysis

Intensity measurements from imaging experiments were normalized for each experiment (see figure legends and graphs). The *n* numbers are shown in figure legends and refer to the number of independent, replicate experiments. Within each experiment, the mean values of every condition were calculated from a minimum of three technical replicates. Integrated density measurements from western blot experiments were normalized to control wells (see figure legends and graphs). Wherever possible, normalization to conditions for statistical comparisons were avoided in order to maintain experimental error associated. GraphPad Prism 9 (La Jolla, CA, USA) was used for statistical analyses and graph production. Data were subjected to either one- or two-way ANOVA with Dunnett’ss *post hoc* analysis for multiple comparisons, unless otherwise stated. All error bars indicate mean ± standard deviation (SD) from replicate experiments.

### Software

LocusZoom 30 (http://locuszoom.org/, RRID:SCR_021374) was used for the regional association plotting of summary statistics from Parkinson’s disease GWAS.^4^ Dotmatics Vortex v.5.1 software was used to check for edge effects and for visualization (https://www.dotmatics.com/capabilities/vortex). GraphPad Prism 9 (La Jolla, CA, USA) was used for statistical analyses and graph production: GraphPad Prism (RRID:SCR_002798) (https://www.graphpad.com/).

### Data availability

Exome and genome variant data is available from Genome Aggregation Database (gnomAD): https://gnomad.broadinstitute.org/about v.2.1.1 and subsequent releases are available for download: https://gnomad.broadinstitute.org/downloads, Google Cloud Public Datasets (https://cloud.google.com/public-datasets), the Registry of Open Data on AWS (https://registry.opendata.aws/) and Azure Open Datasets (https://azure.microsoft.com/en-us/services/open-datasets/).

Data sets from the International Molecular Exchange consortium^42^ (https://www.imexconsortium.org/) used for WPPINA analysis data are reported by Ferrari *et al*.^27^: and available through doi:10.1186/s12864-018-4804-9. 2014 Parkinson’s disease GWAS data^28^ analysed are available through doi:10.1038/ng.3043. 2017 Parkinson’s disease GWAS^3^ data are available through doi:10.1038/ng.3955. Parkinson’s disease GWAS summary statistics^4^ are available through doi:10.1016/S1474-4422(19)30320-5.

Genes expressed in SHSY5Y cells were accessed from expression data deposited in the Human Protein Atlas (proteinatlas.org) and EBI Expression Atlas (https://www.ebi.ac.uk/gxa/home).

Tabulated data for figures in the paper are available through doi:10.5281/zenodo.6952972. The datasets generated, analysed and reported in this paper are available from the corresponding author on reasonable request.

## Results

### Bioinformatic prioritization of Parkinson’s disease GWAS candidates

Genomic analyses of Parkinson’s disease have identified over 80 loci associated with an increased lifetime risk for disease.^3^ In contrast to Mendelian Parkinson’s disease genes, however, the assignment of a causative gene to a risk locus is often challenging. To identify new risk genes for Parkinson’s disease, we undertook a triage of Parkinson’s disease GWAS candidate genes using a combination of methods: (i) Colocalization (Coloc) and transcriptome-wide association analysis^43^ using expression quantitative trait loci (eQTLs) information derived from Braineac,^44^ genotype-tissue expression and CommonMind resources^29,45^ to link Parkinson’s disease risk variants with specific genes, (ii) weighted protein–protein interaction network analysis (WPPINA)^27^ on the basis of Mendelian genes associated with Parkinson’s disease and (iii) the prioritized gene set as described in Parkinson’s disease GWAS.^3,28^ This resulted in the nomination of 31 ORFs as putatively causal for associations at Parkinson’s disease risk loci. Then 55% of these genes were prioritized through multiple techniques, with three out of 31 genes (*KAT8*, *CTSB* and *NCKIPSD*) identified through all three prioritization methods (Supplementary Fig. 1A). The 31 genes, together with seven Parkinson’s disease Mendelian genes and lysosomal storage disorder genes, previously shown to be enriched for rare, probably damaging variants in Parkinson’s disease,^46^ were then taken forward for functional analysis (Table 1).

**Table 1:** Overview of bioinformatic evidence for genes prioritized and taken forward for downstream functional analysis

Gene	ColB	ColG	Protein–protein interaction	GWAS	MPD	MLS
*ATP13A2*					**X**	**X**
*CCNT2*				**X**		
*CD38*	**X**	**X**		**X**		
*CTSB*	**X**	**X**	**X**			
*DDRGK1*				**X**		
*DGKQ*				**X**		
*DJ1*					**X**	
*DNAJC13*					**X**	
*FBXO7*					**X**	
*GALC*	**X**			**X**		**X**
*GBA*			**X**		**X**	**X**
*GPNMB*	**X**	**X**		**X**		
*HSD3B7*		**X**				
*IDUA*						**X**
*INPP5F*			**X**			
*KAT8*		**X**	**X**	**X**		
*KLHL7*		**X**		**X**		
*LRRK2*			**X**	**X**	**X**	
*LSM7*	**X**	**X**		**X**		
*MAPT*			**X**	**X**		
*NCKIPSD*	**X**	**X**	**X**	**X**		
*NEK1*	**X**					
*NSF*			**X**			
*NUCKS1*		**X**		**X**		
*NUPL2*	**X**	**X**		**X**		
*PDLIM2*		**X**		**X**		
*PM20D1*	**X**					
*PRKN*					**X**	
*RAB7L1*	**X**	**X**	**X**			
*SH3GL2*			**X**			
*SLC41A1*		**X**		**X**		
*SNCA*					**X**	
*SPPL2B*	**X**					
*STK39*				**X**		
*VAMP4*	**X**	**X**				
*VPS35*					**X**	
*WDR6*	**X**	**X**				
*ZNF646*				**X**		

ColB = coloc analysis using Braineac; ColG = coloc analysis using genotype-tissue expression; GWAS = genes prioritized in Parkinson’s disease GWAS^3^; MLS = Mendelian genes associated with lysosomal storage disorders; MPD = Mendelian genes associated with Parkinson’s disease; WPPINA = weighted protein interaction network.

### pUb(Ser65)-based screen of prioritized genes identifies KAT8 as novel regulator of PINK1-dependent mitophagy initiation

**Figure 1:**
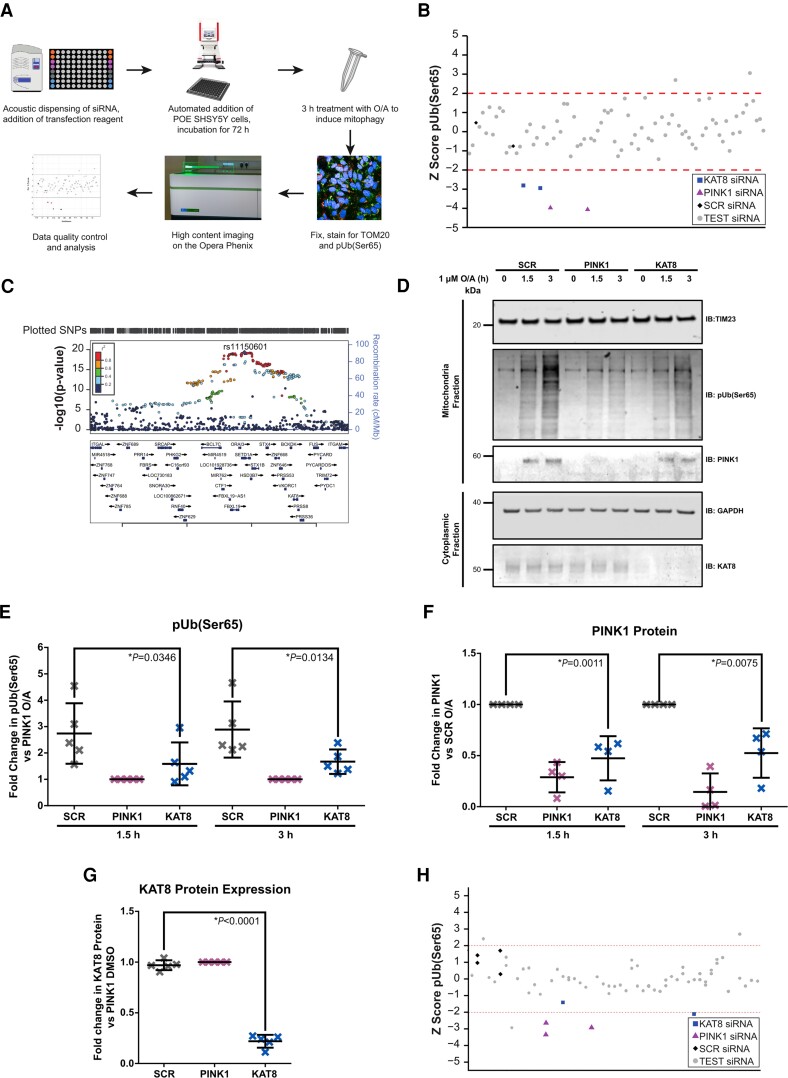
**High-content mitophagy screening of Parkinson’s disease risk genes identifies KAT8 as a modulator of pUb(Ser65) levels.** (**A**) Workflow of the high-content screen for O/A-induced pUb(Ser65) levels. (**B**) pUb(Ser65) *Z* scores of one representative mitophagy screen plate. (**C**) Overview of the Parkinson’s disease GWAS genetic signal at the *KAT8* locus. (**D**) Representative IB of mitochondrial fractions from SCR, PINK1 and KAT8 KD POE SHSY5Y treated with 1 µM O/A for 1.5 or 3 h. (**E**) Quantification of pUb(Ser65) in **E** (*n* = 5, one-way ANOVA with Dunnett’s correction). (**F**) Quantification of PINK1 in **E** (*n* = 4, one-way ANOVA with Dunnett’s correction). (**G**) Quantification of KAT8 in **E** (*n* = 5, one-way ANOVA with Dunnett’s correction). (**H**) pUb(Ser65) *Z* scores of one representative lysine acetyltransferase screen plate. See Supplementary Table 5 for the complete list of the genes screened. Data are shown as mean ± SD.

On the basis of extensive data implicating impaired mitophagy in the aetiology of familial Parkinson’s disease, we hypothesized that Parkinson’s disease GWAS candidate genes, involved in the most common, idiopathic form of the disease, may play a role in this process. To test whether the 38 prioritized genes have a role in PINK1-mitophagy, we developed and optimized a high-content screening assay for phosphorylation of ubiquitin at serine 65 [pUb(Ser65)], a PINK1-dependent mitophagy initiation marker,^47^ following mitochondrial depolarization (Fig. 1A). The 38 prioritized genes were individually KD using siRNA in Parkin overexpressing (POE)-SHSY5Y human neuroblastoma cells. Increased mitochondrial clearance following mitochondrial depolarization induced by treatment with 10 µM of oligomycin/antimycin A (O/A) was validated as an endpoint for mitophagy (Supplementary Fig. 1B). Over 97% of the pUb(Ser65) signal colocalized with the TOM20 mitochondrial marker in O/A treated cells (Supplementary Fig. 1C and D). KD efficiency was validated using both a pool of *PINK1* siRNA, which decreased O/A-induced pUb(Ser65) and subsequent TOM20 degradation (Supplementary Fig. 1E–G) without decreasing cell viability (Supplementary Fig. 2A and B), and a pool of Polo-like kinase 1 (PLK-1) siRNA that decreased cell viability by apoptosis (Supplementary Fig. 2A and B). The siRNA pools for the 38 candidate genes, together with controls, were screened in duplicate on each plate, across three replicate plates per run. Hits were identified as those wells where O/A-induced pUb(Ser65) was decreased or increased at >2 SD from the mean of the scramble (SCR) negative control siRNA.

*KAT8* was selected on the basis of reproducible downregulation of O/A-induced PINK1-dependent pUb(Ser65) across all three replicates (Fig. 1B and Supplementary Fig. 1H), without affecting cell viability (Supplementary Fig. 2C). Notably, *KAT8* was selected as a candidate gene on the basis of all three prioritization criteria: namely, proximity of the lead SNP to an ORF (Fig. 1D), colocalization of a brain-derived eQTL signal with a Parkinson’s disease GWAS association signal (Supplementary Fig. 3) and evidence of protein–protein interaction with a known Parkinson’s disease gene (Table 1). Furthermore, we find that colocalization and transcriptome-wide association analysis^48^ analyses at this locus are consistent with the KD models in the high-content screening assay (Supplementary Tables 3 and 4).^29^ Both methods predict that the risk allele operates by reducing *KAT8* expression in Parkinson’s disease cases versus controls.

The effect of KAT8 KD on pUb(Ser65) was further validated in POE SHSY5Y cells treated with 1 µM O/A, using both IB and IF (Fig. 1D–G and Supplementary Fig. 4). To assess whether other lysine acetyltransferases (KATs) could regulate PINK1-dependent mitophagy, the pUb(Ser65) screen was repeated in POE SHSY5Y cells silenced for 22 other KATs (Supplementary Table 5).^49,50^ Only KAT8 KD led to a decreased pUb(Ser65) signal, emphasizing the specificity of the KAT8 KD effect on pUb(Ser65) (Fig. 1H).

### KANSL1, another NSL member and Parkinson’s disease GWAS candidate, regulates PINK1-dependent mitophagy and dopaminergic neuron viability

These functional data complement and support the omic prioritization of *KAT8* as a causative gene candidate for the chromosome 16q11.2 Parkinson’s disease-associated locus (Fig. 1C). To gain further insight into a possible role for KAT8 in the aetiology of Parkinson’s disease, we explored the known functional interactions of this protein.

**Figure 2:**
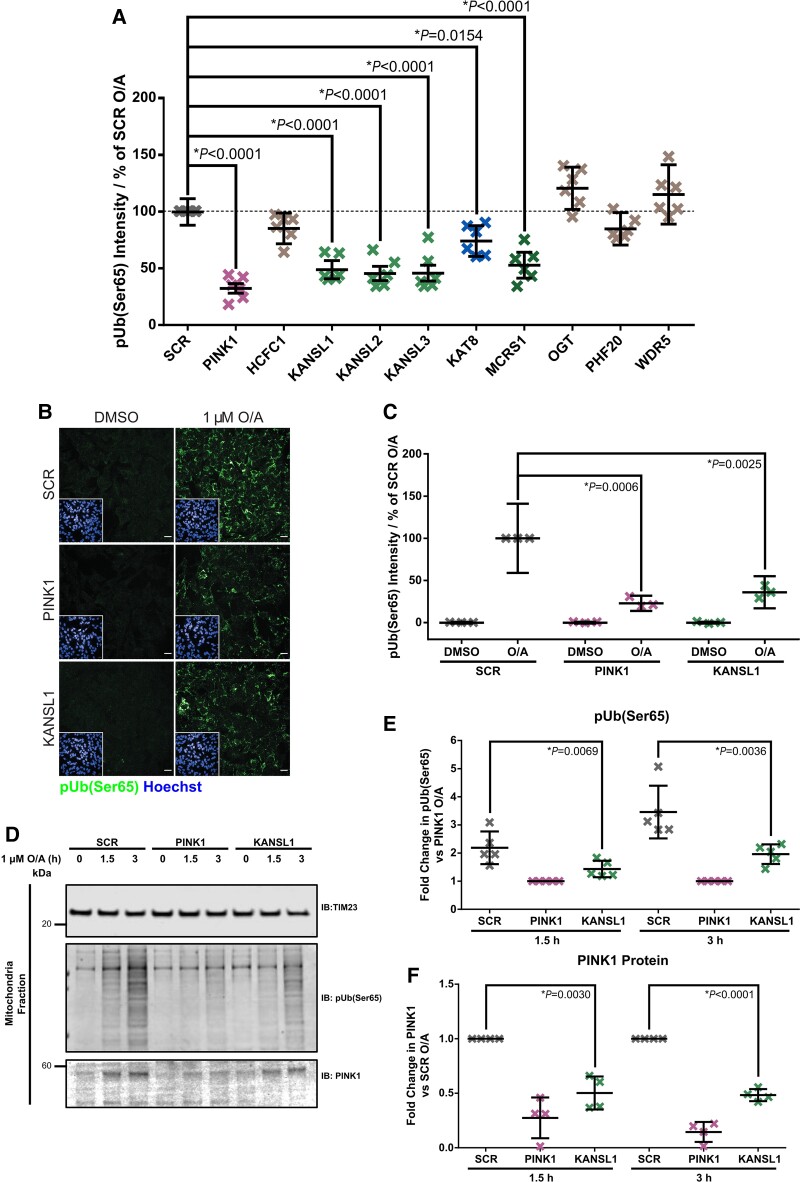
**Knockdown of KANSL1 affects pUb(Ser65) levels.** (**A**) Quantification of pUb(Ser65) following treatment of SCR, PINK1 or NSL components siRNA KD POE SHSY5Y cells with 1 μM O/A for 1.5 h. Data are shown as mean ± SD; *n* = 6, one-way ANOVA with Dunnett’s correction. (**B**) Representative images of pUb(Ser65) (green) following treatment of SCR, PINK1 and KANSL1 KD POE SHSY5Y cells with 1 µM O/A for 3 h. *Insets* show the nuclei (blue) for the same fields. Scale bar = 20 μm. (**C**) Quantification of pUb(Ser65) in **B** (*n* = 3, two-way ANOVA with Dunnett’s correction). (**D**) Representative IB of mitochondrial fractions from SCR, PINK1 and KANSL1 KD POE SHSY5Y treated with 1 μM O/A for 1.5 or 3 h. (**E**) Quantification of pUb(Ser65) in **D** (*n* = 5, one-way ANOVA with Dunnett’s correction). (**F**) Quantification of PINK1 in **D** (*n* = 4, one-way ANOVA with Dunnett’s correction). Data are shown as mean ± SD.

KAT8 has previously been shown to partially localize to mitochondria as part of the NSL complex together with KANSL1, KANSL2, KANSL3 and MCRS1.^24^ To test whether other components of the NSL complex also modulate mitophagy, the pUb(Ser65) screen was repeated in POE SHSY5Y cells silenced for each of the nine NSL components. Notably, reduction of KANSL1, KANSL2, KANSL3, MCRS1 and KAT8 expression led to decreased pUb(Ser65) after 1.5 or 3 h O/A treatment (Fig. 2A and Supplementary Fig. 5), suggesting that the NSL complex modulates PINK1-dependent mitophagy initiation. Interestingly, *KANSL1* is another Parkinson’s disease GWAS candidate gene.^3^ The effect of KANSL1 KD on pUb(Ser65) was further validated in POE SHSY5Y cells treated with 1 µM O/A, using both IF and IB (Fig. 2B–F).

**Figure 3:**
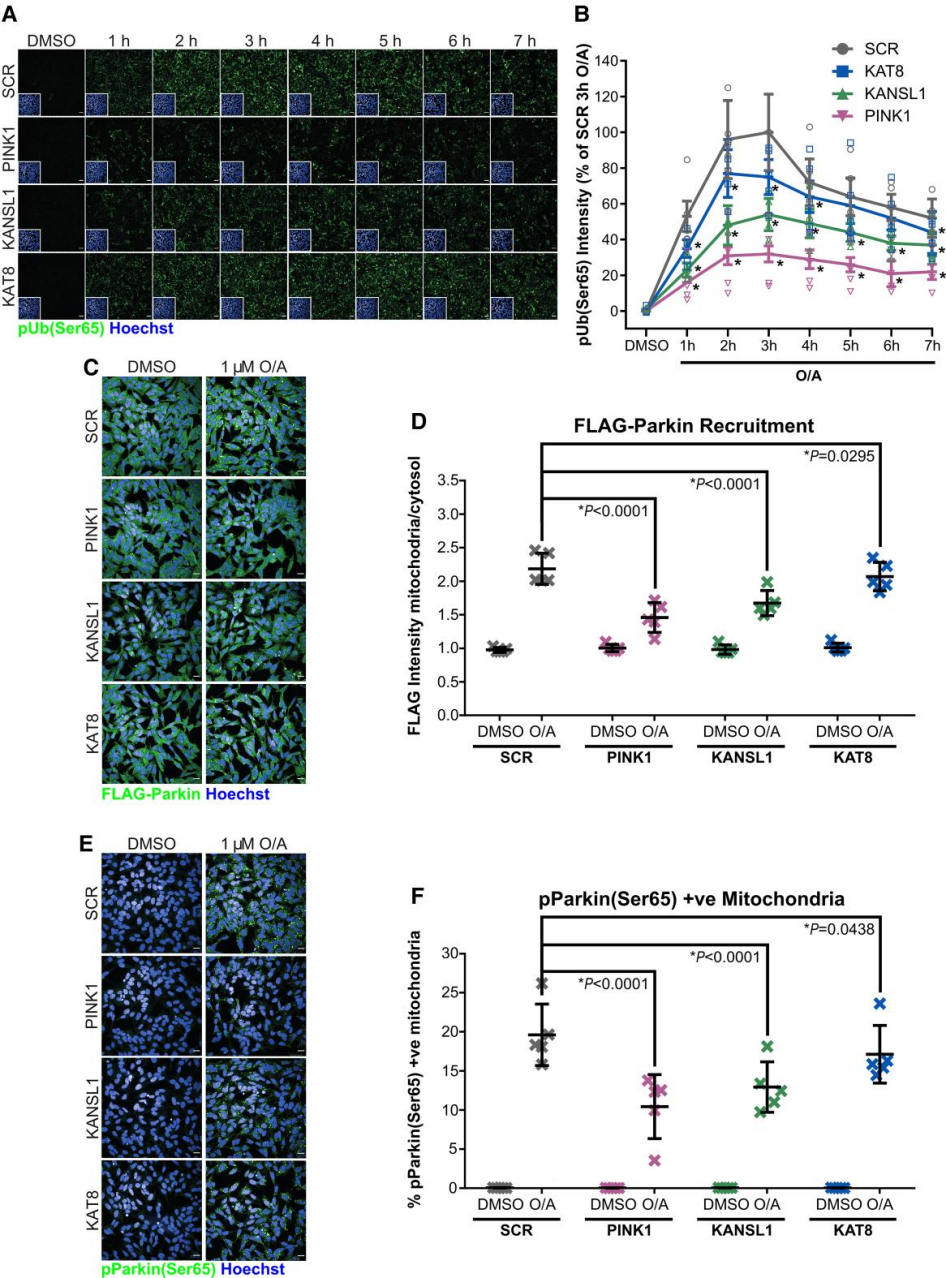
**KAT8 and KANSL1 knockdown decreases PINK1-dependent mitophagy initiation.** (**A**) Representative images of pUb(Ser65) (green) following treatment of SCR, PINK1, KAT8 and KANSL1 KD POE SHSY5Y cells with 1 μM O/A for 0–7 h. Insets show the nuclei (blue) for the same fields. Scale bar = 20 μm. (**B**) Quantification of pUb(Ser65) in **A** (*n* = 6, two-way ANOVA with Dunnett’s correction). For details on the statistical test, see Supplementary Table 6. (**C**) Representative images of FLAG-Parkin (green) with Hoechst nuclei counterstain (blue) following treatment of SCR, PINK1 and KAT8 siRNA KD POE SHSY5Y with 1 µM O/A for 3 h. Scale bar = 20 µm. (**D**) Quantification of FLAG-Parkin recruitment to the mitochondria as a ratio of FLAG intensity in the mitochondria and in the cytosol in **C** (*n* = 5, two-way ANOVA with Dunnett’s correction). (**E**) Representative images of pParkin (green) with Hoechst nuclei counterstain (blue) following treatment of SCR, PINK1 and KAT8 siRNA KD POE SHSY5Y with 1 µM O/A for 3 h. Scale bar = 20 µm. (**F**) Quantification of pParkin levels in **E** (*n* = 5, two-way ANOVA with Dunnett’s correction). Data are shown as mean ± SD.

Deconvolution of the individual siRNA within the respective pools (of 4× individual siRNA against the target gene) (Supplementary Fig. 6) and rescue of the siRNA KD phenotype with overexpression of V5-tagged KANSL1/KAT8 (Supplementary Fig. 7) are both supportive of on-target effect associated with KANSL1/KAT8 KD. The effect of the KAT8 and KANSL1 KD on pUb(Ser65) was confirmed in WT SHSY5Y cells and the astroglioma H4 cell line, both of which are expressing endogenous levels of Parkin (Supplementary Fig. 8). To further assess the effect of KAT8 and KANSL1 KD on PINK1-dependent mitophagy initiation, we measured pUb(Ser65) levels over time (Fig. 3A and B), as well as PINK1 recruitment (Fig. 1D and F and Fig. 2D and F), Parkin recruitment (Fig. 3C and D), PINK1-dependent phosphorylation of Parkin at Ser65 [pParkin(Ser65)] (Fig. 3E and F and Supplementary Fig. 9)^9^ and PINK1-dependent (but indirect) phosphorylation of Rab8A at Ser111 [pRab8A(Ser111)]^25^ (Supplementary Fig. 10). The reduction in pUb(Ser65) levels is not associated with reduced availability of Parkin (Supplementary Fig. 9) or ubiquitin (Supplementary Fig. 11).

**Figure 4:**
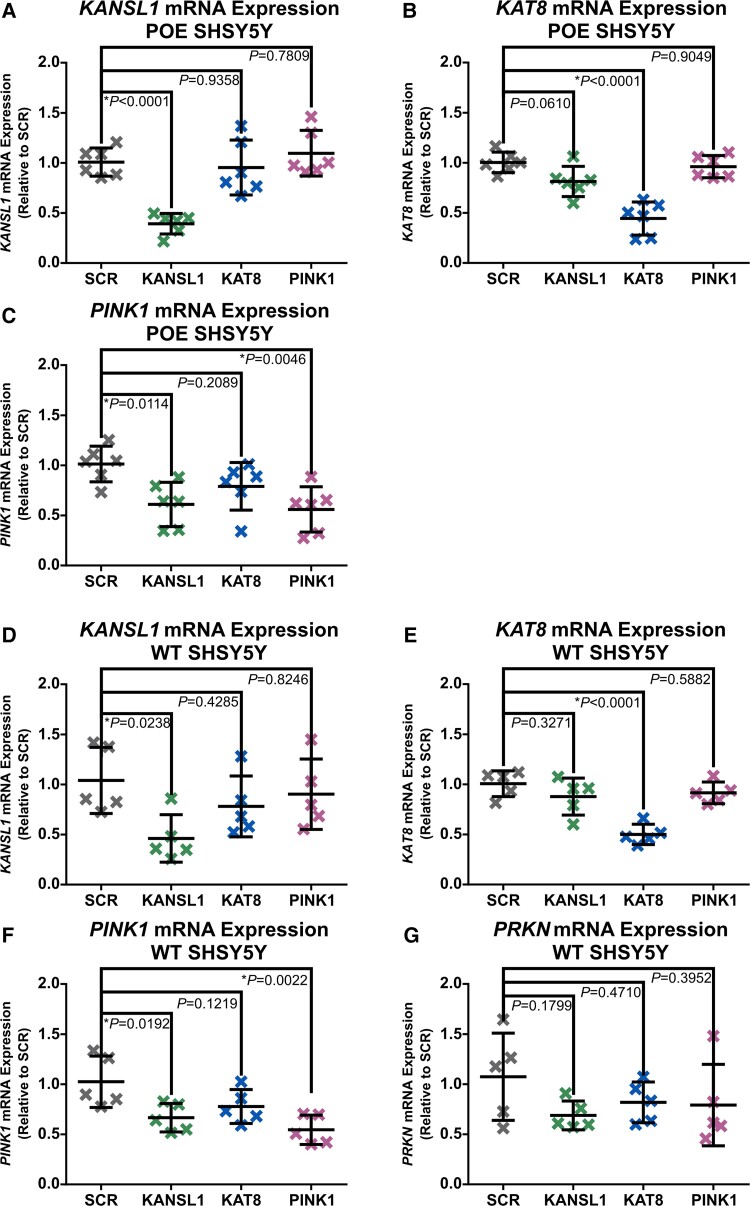
**Real-time qPCR Assessments of *KANSL1*, *KAT8*, *PINK1* and *PRKN* gene expression in POE and WT SHSY5Ys.** (**A**–**C**) Real-time qPCR quantification of *KANSL1* mRNA (**A**), *KAT8* mRNA (**B**) and *PINK1* mRNA (**C**) in POE SHSY5Ys (*n* = 6, one-way ANOVA with Dunnett’s correction). (**D**–**G**) Real-time qPCR quantification of *KANSL1* mRNA (**D**), *KAT8* mRNA (**E**), *PINK1* mRNA (**F**) and *PRKN* mRNA (**G**) in WT SHSY5Ys (*n* = 5, one-way ANOVA with Dunnett’s correction). Data are shown as mean ± SD.

Given the canonical function of the NSL complex as a pro-transcriptional epigenetic remodelling complex.^15,16^ a strong candidate mechanism accounting for the reduced PINK1 protein accumulation and PINK1-dependant mitophagy initiation could be reduced *PINK1* gene expression. In fact, real-time qPCR assessments of *PINK1* mRNA levels in WT and POE SHSY5Ys reveal that KANSL1 and, to a lesser degree, KAT8 KD both reduce *PINK1* gene expression (Fig. 4). These data are mirrored by both *PINK1* gene expression and PINK1 protein accumulation in H4 cells (Supplementary Fig. 8C, D and F–H). Finally, the reduction in *PINK1* mRNA following KANSL1/KAT8 KD in POE SHSY5Ys is also rescued with KANSL1/KAT8 overexpression (Supplementary Fig. 7C, D, G and H).

**Figure 5:**
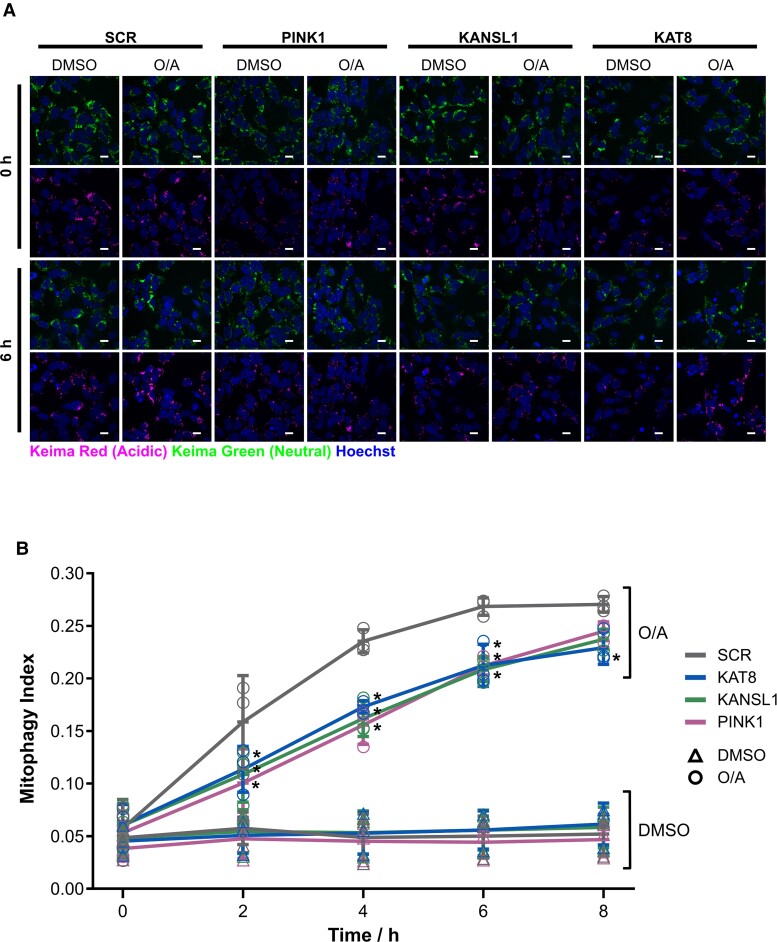
**KANSL1 and KAT8 knockdown decrease mitochondrial clearance.** (**A**) Representative images of mt-Keima following treatment of SCR, PINK1 and KAT8 siRNA KD POE SHSY5Y with 1 µM O/A for 0–8 h. The first and third rows show the neutral Keima-green signal (green) counterstained with Hoechst (blue) after 0 and 6 h, respectively, of DMSO versus O/A. The second and fourth rows show the acidic lysosomal Keima-red signal (red) counterstained with Hoechst (blue) after 0 and 6 h, respectively, of DMSO versus O/A. Scale bar = 25 µm. (**B**) Quantification of the mitophagy index, calculated as the ratio of the area of lysosomal mt-Keima signal and total mt-Keima signal in **A** (*n* = 3, one-way ANOVA with Dunnett’s correction). For details on the statistical test, see Supplementary Table 7. Data are shown as mean ± SD.

KD of both KAT8 and KANSL1 reduced subsequent mitochondrial clearance in live POE-SHSY5Y cells, as measured by the mitophagy reporter mt-Keima^51^ (Fig. 5).

To assess the role of KAT8/KANSL1 in neuronal function and survival *in vivo*, we used *Drosophila* as a model system. Notably, the NSL complex was originally discovered in *Drosophila* through the homologs of *KAT8* and *KANSL1* (*mof* and *nsl1*, respectively), but null mutations for these genes are associated with developmental lethality owing to profound transcriptional remodelling during development.^52^ Therefore, we used inducible transgenic RNAi strains to target the KD of *mof* and *nsl1* specifically in neuronal tissues. Using behavioural assays as a sensitive readout of neuronal function we found that pan-neuronal KD of *mof* or *nsl1* caused progressive loss of motor (climbing) ability (Supplementary Fig. 12A and B), and also significantly shortened lifespan (Supplementary Fig. 12C and D). Interestingly, loss of *nsl1* had a notably stronger effect than loss of *mof*. Consistent with this, KD of *nsl1* but not *mof*, in either all neurons or only in dopaminergic (DA) neurons, caused the loss of DA neurons (Supplementary Fig. 12E and F).

### KANSL1 is a likely Parkinson’s disease GWAS candidate at the 17q21 locus

**Figure 6:**
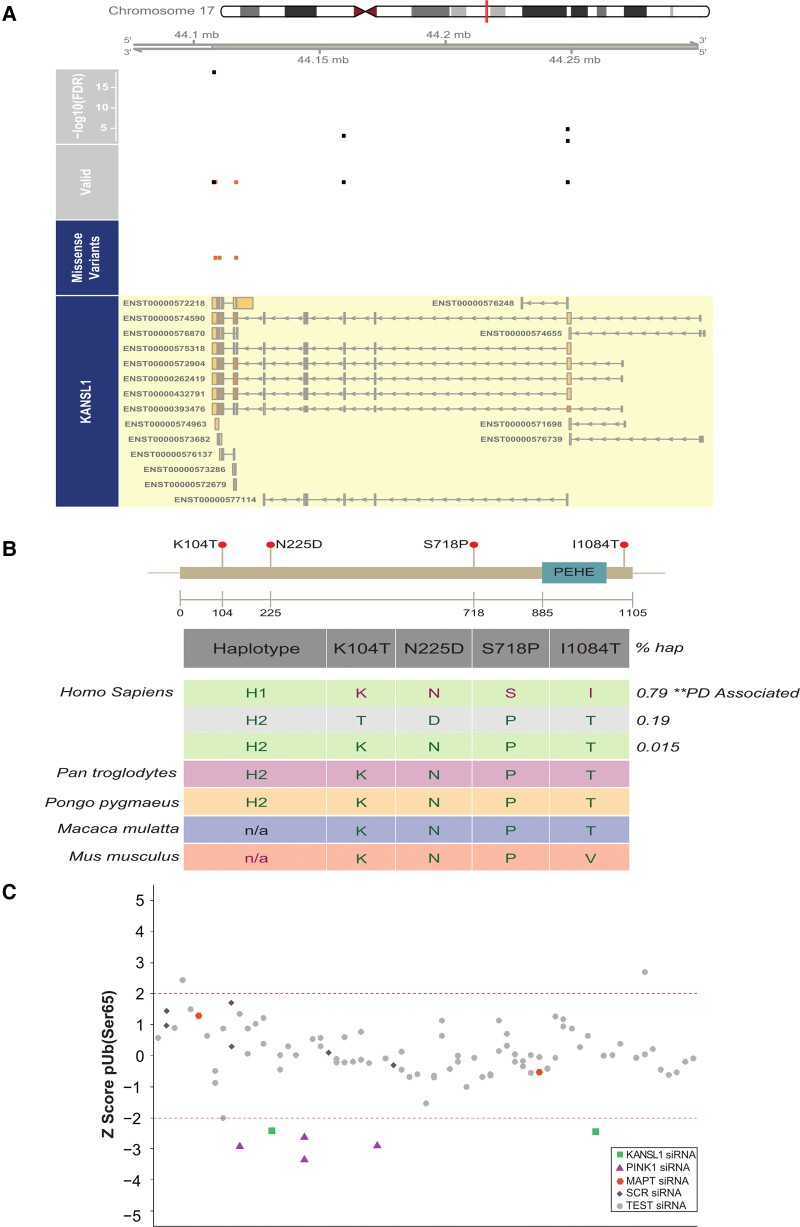
***KANSL1* presents ASE sites in LD with the H1/H2 SNP, and pUb(Ser65) levels are altered by siRNA KD of KANSL1 but not other genes present at the 17q21 locus**. (**A**) ASEs derived from putamen and substantia nigra in high LD with the H1/H2 tagging SNP, rs12185268 and their position along the *KANSL1* gene. The missense variants track displays the variants annotated as missense by gnomAD v.2.1.1.^70^ The valid track displays the heterozygous sites (orange = missense) with an average read depth >15 reads across all samples, which were examined for ASE. The topmost track displays the false discovery rate-corrected minimum −log_10_*P*-value across samples for the sites that show an ASE in at least one sample. (**B**) Conservation of the KANSL1 protein across species. The four coding variants (NM_001193465) in the *KANSL1* gene are in high LD (*r*^2^ > 0.8) with the H1/H2 haplotypes. (**C**) pUb(Ser65) *Z* scores of one representative 17q21 locus screen plate. See Supplementary Table 10 for the complete list of the genes screened.

*KANSL1* is located within the extensively studied inversion polymorphism on chromosome 17q21 (Supplementary Fig. 13A and B), which also contains *MAPT*—a gene frequently postulated to drive Parkinson’s disease risk at this locus.^53^ While the majority of individuals inherit this region in the direct orientation, up to 25% of individuals of European descent have a ∼1 Mb sequence in the opposite orientation,^54,55^ inducing a larger ∼1.3–1.6 Mb region of LD. Since this inversion polymorphism precludes recombination over a region of ∼1.3–1.6 Mb, haplotype-specific polymorphisms have arisen resulting in the generation of two major haplotype clades, termed H1 (common haplotype) and H2 (inversion carriers), with H1 previously strongly linked to neurodegenerative disease including Parkinson’s disease.^56–58^ Due to high LD, the genetics of this region have been hard to dissect, and robust eQTL analyses have been challenging due to the issue of polymorphisms within probe sequences in microarray-based analyses or mapping biases in RNA-seq-based analyses. Several variants (rs34579536, rs35833914 and rs34043286) are in high LD with the H1/H2 haplotype and are located within *KANSL1* (Fig. 6A and B), raising the possibility that they could directly affect KANSL1 protein function. In particular, one of the variants is associated with a serine (H1 haplotype) or proline (H2 haplotype) amino acid change in KANSL1 protein sequence (NM_001193465:c.T2152C:p.S718P), and would therefore be predicted to alter the gross secondary structure of the KANSL1 protein (Fig. 6B). Furthermore, we explored the possibility that Parkinson’s disease risk might be mediated at this locus through an effect on *KANSL1* expression. Using RNA-seq data generated from 84 brain samples (substantia nigra *n* = 35; putamen *n* = 49), for which we had access to whole exome sequencing and SNP genotyping data thus enabling mapping to personalized genomes,^39^ we performed ASE analysis. More specifically, we quantified the variation in expression between the H1 and H2 haplotypes (Supplementary Table 8) amongst heterozygotes. While we identified ASE sites within *MAPT* (Supplementary Fig. 14 and Supplementary Table 9), we also identified four sites of ASE in *KANSL1* (Fig. 6A), suggesting that the high Parkinson’s disease risk H1 allele is associated with lower *KANSL1* expression, consistent with our functional assessment. Interestingly, sequence analysis of the human *KANSL1* haplotype revealed that the high risk H1 haplotype is the more recent ‘mutant’ specific to *Homo sapiens*, and that other primates and mammals share the rarer non-risk ancestral H2 haplotype (Fig. 6B). To assess the specificity of the KANSL1 KD effect on PINK1-dependent mitophagy initiation, 32 ORFs in LD on the H1 haplotype at the 17q21 locus (Supplementary Fig. 13A and B and Supplementary Table 10) were knocked down individually and their effect on pUb(Ser65) was assessed. While the effect of KANSL1 KD on pUb(Ser65) was confirmed, neither the KD of MAPT (see also Supplementary Fig. 15), nor the KD of each of the other 30 genes on this locus, led to a decrease in the pUb(Ser65) signal (Fig. 6C). These data confirm the selectivity of our mitophagy screening assay and suggest that *KANSL1* is likely to be a key Parkinson’s disease risk gene at the 17q21 locus.

### Impairments in PINK1-dependent mitophagy initiation are also observed in human iNeuron models of KANSL1 and KAT8 deficiency

Finally, we sought to validate the mitophagy initiation impairments associated with reductions in KANSL1 and KAT8, in more disease-relevant hiPSC derived neuron systems.

**Figure 7:**
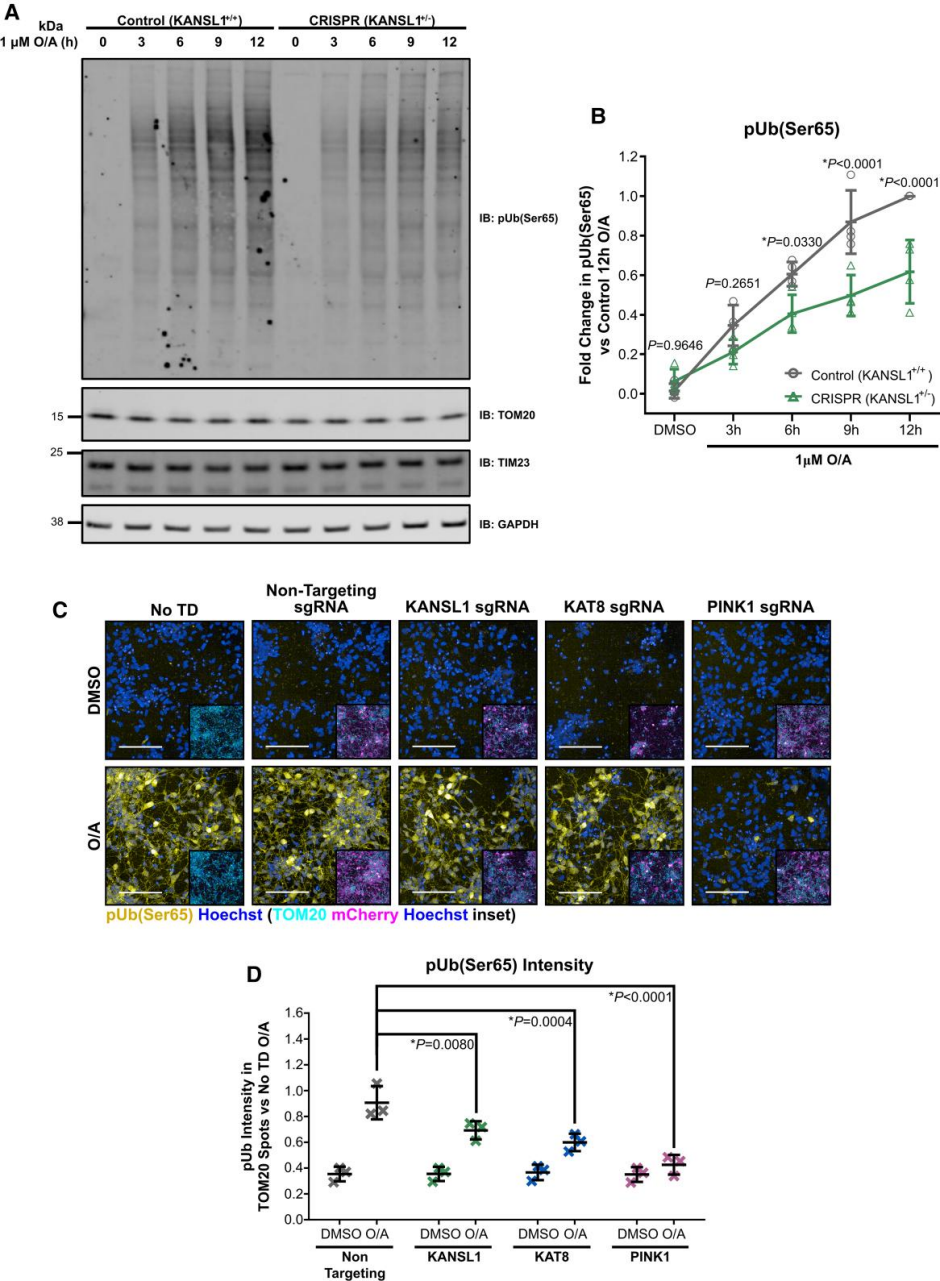
**pUb(Ser65) levels are reduced in isogenic iNeurons with heterozygous KANSL1^+/-^ loss of function and CRISPRi-i3N iNeurons with KANSL1 and KAT8 sgRNA KD.** (**A**) Representative IB of isogenic d17 iNeurons with/without heterozygous LoF frameshift mutation in KANSL1 treated with 1 µM O/A over a 12 h extended time-course. (**B**) Quantification of pUb(Ser65) in **A** (*n* = 4 inductions, two-way ANOVA with Dunnett’s correction). (**C**) Representative images of pUb(Ser65) (yellow) with Hoechst nuclei counterstain (blue) following treatment of non-transduced (No TD), non-targeting, KANSL1, KAT8 and PINK1 sgRNA KD d17 CRISPRi-i3N iNeurons with 1 µM O/A versus DMSO for 9 h. Inserts show staining for TOM20 (cyan) and mCherry transduction reporter (magenta) with Hoechst nuclei counterstain (blue) for the same field of view. Scale bar = 100 µm. (**D**) Quantification of pUb(Ser65) intensity in TOM20 defined mitochondrial area in **D** (*n* = 3 inductions, two-way ANOVA with Dunnett’s correction). Data are shown as mean ± SD.

To this end, further experiments were performed using isogenic hiPSC lines with or without a heterozygous LoF frameshift mutation (c.531insT) in KANSL1 introduced through CRISPR–Cas technology.^22^ These lines have also been stably transduced with transgenes conferring overexpression of murine neurogenin-2 (Ngn2) under a tetracycline-ON (TET-ON) system, permitting differentiation into human cortical neurons following doxycycline treatment (iNeurons). After 17 days *in vitro* (DIV), KANSL1 control (KANSL1^+/+^) and heterozygous LoF (KANSL1^+/-^) iNeurons with ∼50% reduction in *KANSL1* gene expression (Supplementary Fig. 16A) were subjected to assessments of mitophagy initiation. Treatment of KANSL1^+/+^ iNeurons with 1 µM O/A resulted in pUb(Ser65) deposition, which continued to increase across a prolonged 12 h treatment window (Fig. 7A and B). While KANSL1^+/-^ iNeurons showed detectable pUb(Ser65) deposition, the levels were lower than that of the isogenic KANSL1^+/+^ iNeurons, with this difference being significant at 9 and 12 h of O/A treatment (Fig. 7B).

Similar experiments were also performed using the CRISPRi-i3N iNeuron system, which in addition to the presence of a TET-ON system for dox-induced Ngn2 mediated neuronal differentiation,^37^ also express an enzymatically dead Cas9-KRAB transcriptional repressor fusion protein that permits target gene KD through introduction of sgRNA molecules for specific gene promoters.^38^ Similar to observations in the KANSL1^+/−^ iNeurons and KANSL1/KAT8/PINK1 siRNA KD cell line models, CRISPRi KD of all three gene targets (Supplementary Fig. 16B–D) significantly reduced mitochondrial pUb(Ser65) following 9 h O/A treatment as assessed through IF (Fig. 7C and D).

These data support an important functional link between KANSL1, KAT8 and PINK1-dependent mitophagy initiation. While our data support reductions in *PINK1* gene expression as the most likely underlying mechanism for mitophagy impairment in KAT8 and KANSL1 deficient cell lines, further experiments will be required to determine whether KAT8/KANSL1-dependent *PINK1* gene expression regulation also explains mitophagy impairments in other cellular and *in vivo* models, and/or whether other mechanisms could be involved.

## Discussion

Since the first Parkinson’s disease GWAS study was performed in 2006,^59^ GWAS have identified >80 independent loci for Parkinson’s disease.^4^ However, translating GWAS findings into a new molecular understanding of Parkinson’s disease-associated pathways and new therapeutic targets has remained a major challenge for the scientific community. To screen for Parkinson’s disease GWAS candidate genes that play a role in PINK1-mitophagy, and thus are likely to be genuine risk genes for Parkinson’s disease, we have set up and optimized a high-content screening for pUb(Ser65), a marker of PINK1-dependent mitophagy initiation, a key pathway in Parkinson’s disease pathogenesis. This approach allowed the successful identification of two new genes associated with increased Parkinson’s disease risk, that play a role in mitophagy. Interestingly, these two genes were previously shown to be part of the same complex, the NSL complex.

This study demonstrates the substantial potential of functional screens to exploit genetic data by providing orthogonal information that can confidently identify new risk genes. This is particularly important in genomic regions with uniformly high LD, such as the 17q21 inversion region that includes 32 ORFs of which many are highly expressed in brain and where existing fine-mapping and functional genomic analyses have been inconclusive. Interestingly, while *MAPT* has long been considered the risk associated gene at this locus, this has recently been questioned by Dong and colleagues, who also raised the significance of KANSL1 in driving Parkinson’s disease risk at the locus.^60^ In line with our data, eQTL analysis by other groups has shown the Parkinson’s disease risk H1 haplotype is associated with reduced *KANSL1* mRNA expression,^61,62^ and non-synonymous H1 versus H2 haplotype KANSL1 amino acid changes including K104T, N225D, S718P and I1084 we describe in this paper have been confirmed to be in high LD through Sanger sequencing by another group.^63^ Similar to our data, eQTL analysis by others has revealed that lower levels of *KAT8* gene expression are also linked to an increased Parkinson’s disease risk.^3^

Furthermore, functional screening can simultaneously provide mechanistic insights as exemplified in this case by the novel insights we provide into the molecular events regulating mitochondrial quality control and which support a role for mitophagy as a contributing factor to sporadic Parkinson’s disease. KANSL1 is part of the NSL complex and functions as a scaffolding protein by binding other subunits, including KAT8.^14^ Through the deposition of pro-transcriptional histone acetylation marks, the NSL complex underscores an important regulator of target gene expression.^16^ In this study, we show that PINK1-dependent phosphorylation of ubiquitin is reduced in the context of KANSL1 and KAT8 LoF and that this appears to be largely caused by a reduction in *PINK1* gene expression, at least in the cell line models. We hypothesize that as a consequence of reduced *PINK1* gene and PINK1 protein expression, mitochondrial accumulation of activated PINK1 is reduced, leading to reduced pUb(Ser65) deposition, Parkin activation and subsequent mitochondrial clearance.

It was previously shown that depletion of KAT8/KANSL1 causes significant downregulation of mitochondrial DNA transcription and translation, and ultimately impaired mitochondrial respiration.^24^ Future studies will need to determine whether KAT8/KANSL1-dependent modulation of mitochondrial DNA or nuclear DNA encoded mitochondrial genes could regulate PINK1 mitochondrial accumulation, activation and subsequent mitophagy. It has been further proposed that the KAT8/KANSL1 complex has targets in the mitochondria other than the mitochondrial DNA.^24^ It will be interesting to determine whether the KAT8/KANSL1 complex could acetylate ubiquitin, which has previously been shown to be acetylated on six out of its seven lysines (K6, K11, K27, K33, K48, K63).^64^ It will also be interesting to understand further whether the regulation of mitophagy related genes such as *PINK1*, is associated with a direct effect of the NSL complex at target gene promoters, or as a consequence of other upstream biochemical cascades, as previously described in the context of KANSL1 LoF.^22^

KANSL1 haploinsufficiency caused by heterozygous pathogenic genetic variants in KANSL1 is associated with the neurodevelopmental disorder Koolen-de Vries syndrome (KdVS; OMIM no. 610443).^65^ Pathogenic variants in KAT8 have also been associated with developmental disorders inclusive of a strong neurological phenotype,^66^ underpinning the functional importance of the NSL complex in neurodevelopment. Using iNeurons differentiated from KdVS-derived patient hiPSCs and genome-edited KANSL1 heterozygous hiPSCs, we have recently shown that KANSL1 deficiency leads to impairments in autophagic flux and lysosomal function that appear to be largely caused by elevated cellular reactive oxygen species,^22^ and impairments in the transcriptional regulation of autophagy related genes have also been described elsewhere in the context of both KAT8 and KANSL1 LoF.^20,23^ Together with our current work highlighting *KANSL1* and *KAT8* as Parkinson’s disease risk genes, these studies suggest that impairments in the autophagic process could be a contributing pathomechanism for idiopathic disease. Alongside our own data implicating impairments in PINK1-dependent mitophagy initiation, synergistic dysregulation in autophagosome and lysosomal-dependent steps, downstream in the mitophagy process, highlight clearance of damaged mitochondria as a particular vulnerability in the context of KAT8/KANSL1 LoF. While more severe KAT8/KANSL1 haploinsufficiency leads to impaired neurodevelopment, more subtle changes in KAT8/KANSL1 and associated mitochondrial deficits, associated with impaired bulk autophagy might lead to accumulation of cellular damage leading to selective vulnerability of dopaminergic neurons later in life.

Our data highlight the use of a cellular function high-content siRNA KD screen for prioritization of GWAS candidates, however, it is important to be aware of limitations to the use of such a strategy. While we have confirmed successful KD of KAT8 and KANSL1, without evaluating KD of all genes screened (which can be challenging with high-throughput screens), the potential pitfall for false negatives remains. siRNA KD strategies are limited to delineating the functional effect of reductions in the expression of a target gene, whereas increased expression might be more disease and functionally relevant in some cases.

Important genetic discoveries in Parkinson’s disease, in particular, the identification of the *PINK1*^67^ and *PRKN* genes,^68^ opened the field of selective mitophagy.^7^ However, there is still a clear need for a better molecular understanding of mitochondrial quality control. Here we provide new insights into the mechanism by identifying two new molecular players, KAT8 and KANSL1. These new regulators of mitophagy provide the first direct evidence for a role of the PINK1-mitophagy pathway in idiopathic Parkinson’s disease and the convergence between familial and idiopathic pathways in disease. Taken together, these findings open a novel avenue for the therapeutic modulation of mitophagy in Parkinson’s disease, with potential implications across drug discovery in frontotemporal dementia and Alzheimer’s disease, where mitophagy also plays an important role in disease pathogenesis.^69^

## Supplementary Material

awac325_Supplementary_Data
