# Supplementary material for: Regulation of mitophagy by the NSL complex underlies genetic risk for Parkinson’s disease at 16q11.2 and MAPT H1 loci
Source: Brain. 2022 Sep 8;145(12):4349–67. doi: 10.1093/brain/awac325 (PMC9762952; doi:10.1093/brain/awac325)
Supplement: awac325_Supplementary_Data [file awac325_supplementary_data.zip › brain-2021-02242-File010.pdf]

EXTENDED FIGURE LEGENDS

Extended Data Figure 1 - High Content siRNA Screen for modulators of pUb(Ser65).

A. Venn diagram highlighting the three genes prioritised by means of three prediction techniques.

B. Fold decrease in TOM20 levels following 1.5 and 3 h treatment with 0.1, 1 and 10 µM O/A, compared to DMSO control.

C. Representative images of TOM20 (red) and pUb(Ser65) (green) with Hoechst nuclei counterstain (blue) following 3 h treatment of SCR KD POE SHSY5Y cells with 10 µM O/A. Scale bar = 20 µm.

D. Quantification of the co-localization in **C** as % of TOM20-positive pUb(Ser65) spots. Graph shows all replicates of non-transfected, SCR, PINK1 and PLK1 KD for 3 independent experiments.

E. Representative images of pUb(Ser65) (green) with Hoechst nuclei counterstain (blue) following treatment of SCR and PINK1 KD POE SHSY5Y cells with 10 µM O/A for 3 h. Scale bar = 20 µm.

F. Quantification of pUb(Ser65) in **E** (n=6, two-way ANOVA with Tukey's multiple comparisons test).

G. Representative analysis of integrated intensity of pUb(Ser65) and TOM20 for a single HCS plate.

H. pUb(Ser65) Z-scores of the two other replicate screen plates.

Data are shown as mean ± SD.

Extended Data Figure 2 - KAT8 knockdown has no effect on cell viability.

A. Representative images of nuclei following treatment of SCR, PINK1 and PLK1 siRNA KD POE SHSY5Y cells with 10 µM O/A for 3 h. Scale bar = 20 µm.

B. Quantification of the number of nuclei in A (n=6, two-way ANOVA with Tukey's multiple comparisons test).

C. Z-scores of a representative screen plate showing that KAT8 or PINK1 siRNA KD don't affect cell viability, on the contrary to PLK-1 KD.

Data are shown as mean ± SD.

Extended Data Figure 3 - KAT8 eQTLs colocalise with SNPs associated with PD risk

The x-axis displays the physical position on chromosome 16 in megabases. The minus log p-values are plotted for every SNP present in both the PD GWAS [3] and *KAT8* eQTLs derived from the GTEx V7 caudate data. The p-values for the PD GWAS are plotted in yellow and p-values for *KAT8* eQTLs are plotted in blue.

Extended Data Figure 4 - KAT8 knockdown decreases pUb(Ser65) levels.

A. Representative images of pUb(Ser65) (green) following treatment of SCR, PINK1 and KAT8 siRNA KD POE SHSY5Y with 1 µM O/A for 3 h. Insets show Hoechst nuclei counterstaining (blue) for the same fields. Scale bar = 20 µm.

B. Quantification of pUb(Ser65) levels in A (n=3, two-way ANOVA with Dunnett's correction).

Data are shown as mean ± SD.

Extended Data Figure 5 - Knockdown of components of the NSL complex reduces pUb(Ser65) levels.

Quantification of pUb(Ser65) following treatment of SCR, PINK1 or NSL components siRNA KD POE SHSY5Y cells with 1 µM O/A for 3 h. Data are shown as mean ± SD; n=6, one-way ANOVA with Dunnett's correction.

Data are shown as mean ± SD.

Extended Data Figure 6 – Deconvolution of the KANSL1 and KAT8 siRNA pools

A. Representative pUb(Ser65) IB of SCR and individual KANSL1 siRNA KD POE SHSY5Y treated with 1 µM O/A for 3 h.

B. Quantification of pUb(Ser65) in A (n=6, two-way ANOVA with Dunnett's correction).

C. RT-qPCR validation of KANSL1 siRNA KD vs SCR for the samples used in A-B (n=6, one-way ANOVA with Dunnett's correction).

D. Representative pUb(Ser65) and KAT8 IB of SCR and individual KAT8 siRNA KD POE SHSY5Y treated with 1 µM O/A for 3 h.

E. Quantification of pUb(Ser65) in D (n=6, two-way ANOVA with Dunnett's correction).

F. RT-qPCR validation of KAT8 siRNA KD vs SCR for the samples used in D-E (n=6, one-way ANOVA with Dunnett's correction).

G. Quantification of KAT8 in D for DMSO treated cells (n=6, one-way ANOVA with Dunnett's correction).

Data are shown as mean ± SD.

Extended Data Figure 7 – Rescue of pUb(Ser65) and PINK1 mRNA deficits in KANSL1 and KAT8 siRNA KD cells with V5-KANSL1 and V5-KAT8 overexpression

A. Representative pUb(Ser65), KAT8 and V5-tag IBs of SCR and KAT8 KD POE SHSY5Y with/without V5-KAT8 overexpression treated with 1 µM O/A for 3 h.

B. Quantification of pUb(Ser65) in A (n=4, two-way ANOVA with Dunnett's correction).

C-D. RT-qPCR of SCR and KAT8 KD POE SHSY5Y with/without V5-KAT8 overexpression. KAT8 mRNA expression (C), PINK1 mRNA expression (D) (n=4, one-way ANOVA with Dunnett's correction). Note broken axis in (C).

E. Representative pUb(Ser65), KANSL1 and V5-tag IBs of SCR and KAT8 KD POE SHSY5Y with/without V5-KANSL1 overexpression treated with 1 µM O/A for 3 h.

F. Quantification of pUb(Ser65) in C (n=4, two-way ANOVA with Dunnett's correction).

Data are shown as mean ± SD.

G-H. RT-qPCR of SCR and KANSL1 KD POE SHSY5Y with/without V5-KANSL1 overexpression. KANSL1 mRNA expression (G), PINK1 mRNA expression (H) (n=4, one-way ANOVA with Dunnett's correction). Note broken axis in (G).

Extended Data Figure 8 - KAT8 and KANSL1 knockdown reduce pUb(Ser65) levels in WT SHSY5Y and H4 cells.

A. Representative images of pUb(Ser65) (green) following treatment of SCR, PINK1 and KAT8 siRNA KD WT SHSY5Y with 1 µM O/A for 3 h. Insets show Hoechst nuclei counterstaining (blue) for the same fields. Scale bar = 20 µm.

B. Quantification of pUb(Ser65) levels in A (n=6, two-way ANOVA with Dunnett's correction).

C. Representative IB of whole-cell lysates from SCR, PINK1, KANSL1 and KAT8 siRNA KD H4 cells treated with 1 µM O/A for 3 h.

D. Quantification of PINK1 in D (n=3, one-way ANOVA with Dunnett's correction).

E. Quantification of pUb(Ser65) in D (n=3, one-way ANOVA with Dunnett's correction).

Data are shown as mean ± SD.

F-H. RT-qPCR of KANSL1 and KAT8 KD H4 cells. *KANSL1* mRNA expression (F), *KAT8* mRNA expression (G), *PINK1* mRNA expression (H) (n=4, one-way ANOVA with Dunnett's correction).

Data are shown as mean ± SD.

Extended Data Figure 9 - KAT8 and KANSL1 knockdown reduce PINK1-dependent phosphorylation of Parkin at Ser65, but not total FLAG-Parkin levels

A. Representative pParkin(Ser65) and FLAG-Parkin IBs of SCR, KANSL1, KAT8 and PINK1 KD POE SHSY5Y treated with 1 µM O/A for 3 h.

B. Quantification of pParkin(Ser65) in A normalised to FLAG-Parkin (n=4, two-way ANOVA with Dunnett's correction).

C. Quantification of FLAG-Parkin in A normalised to GAPDH loading control (n=4, two-way ANOVA with Dunnett's correction).

Samples shown are the same as those used in Extended Data Figure 10.

Data are shown as mean ± SD.

Extended Data Figure 10 – KANSL1 and KAT8 reduce PINK1-dependent phosphorylation of Rab8A (pRab8A(Ser111)) in PINK1 OE SHSY5Ys

A. Representative pRab8A(Ser111) and total Rab8A IB of Rab8A IPs from SCR, KANSL1 and KAT8 PINK1 OE SHSY5Ys

B. Quantification of the pRab8A(Ser111)/Rab8A ratio in A (n=4, two-way ANOVA with Dunnett's correction).

Data are shown as mean ± SD.

Extended Data Figure 11 – pUb(Ser65) deposition but not total-Ub availability is reduced following KANSL1 and KAT8 KD

A. Representative pUb(Ser65) IB of SCR, KANSL1, KAT8 and PINK1 KD POE SHSY5Y treated with 1 µM O/A for 3 h.

B. Representative total-Ub IB of SCR, KANSL1, KAT8 and PINK1 KD POE SHSY5Y treated with 1 µM O/A for 3 h.

C. Quantification of pUb(Ser65) in A normalised to GAPDH loading control (n=4, two-way ANOVA with Dunnett's correction).

D. Quantification of pUb(Ser65) in A normalised to total-Ub in B (n=4, two-way ANOVA with Dunnett's correction).

E. Quantification of Ub(1) monomer in B normalised to GAPDH loading control (n=4, two-way ANOVA with Dunnett's correction).

F. Quantification of poly-Ub / Protein-Ub (>8kDa) in B normalised to GAPDH loading control (n=4, two-way ANOVA with Dunnett's correction).

G. Representative KAT8 IB of SCR, KANSL1, KAT8 and PINK1 KD POE SHSY5Y treated with 1 µM O/A for 3 h.

H. Quantification of KAT8 in G for DMSO treated cells (n=4, one-way ANOVA with Dunnett's correction).

Data are shown as mean ± SD.

Extended Data Figure 12 - Neuronal loss of *mof* or *nsl1* causes locomotor deficit, shortened lifespan and neurodegeneration.

A, B. Climbing ability of pan-neuronal (*nSyb-GAL4*) driven knockdown of *mof* (**A**) or *nsl1* (**B**) measured at the indicated age of adults, compared to control RNAi (A: Kruskal-Wallis test, with Dunn's post-hoc multiple comparisons; B: Mann-Whitney test).

C, D. Lifespan of ***mof* (C) or *nsl1* (D)** pan-neuronal knockdown (*nSyb-GAL4*) compared to control RNAi (Log-rank (Mantel-Cox) test).

E, F. Quantification of dopaminergic neurons (PPL1 cluster) after pan-neuronal or dopaminergic (DA) neuron (*TH-GAL4*) driven depletion of *mof* (**E**), nsl1 (**F**), or control RNAi. Representative images of PPL1 neurons (as bounded by the box) under depletion conditions are shown. Flies were aged 30 days, except for pan-neuronal *nsl1* kd which are 16-days-old. Scale bar = 20 µm; Mann-Whitney test.

For all tests, n numbers are indicated in the graphs; $p<0.0001$ = ****; $p<0.001$ = ***.

Extended Data Figure 13 - Overview of the PD GWAS genetic signal at the *MAPT* locus.

A. *MAPT* primary GWAS signal.

B. *MAPT* conditional GWAS signal.

Extended Data Figure 14 - ASE sites in *MAPT* in LD with the H1/H2 SNP.

ASEs derived from putamen and substantia nigra that are in LD with the H1/H2 tagging SNP, rs12185268 and their position along the *MAPT* gene. The missense variants track displays the variants annotated as missense by gnomAD v2.1.1 [70]. The valid track displays the heterozygous sites (orange = missense) with an average read depth greater than 15 reads across all samples, in LD with H1/H2, which were examined for ASE. The topmost track displays the –log10 scale for the minimum FDR across samples for the sites that show an ASE in at least one sample.

Extended Data Figure 15 – KANSL1 and KAT8 KD but not Tau KD reduce pUb(Ser65)

A. Representative pUb(Ser65) IB of SCR, KANSL1, KAT8, MAPT and PINK1 KD POE SHSY5Y treated with 1 µM O/A for 3 h.

B. Quantification of pUb(Ser65) in A (n=3, one-way ANOVA with Dunnett's correction).

C. Representative KAT8 IB of SCR, KANSL1, KAT8, MAPT and PINK1 KD POE SHSY5Y treated with 1 µM O/A for 3 h.

D. Quantification of KAT8 in C for DMSO treated cells (n=3, one-way ANOVA with Dunnett's correction).

E. Representative Total-Tau IB of SCR, KANSL1, KAT8, MAPT and PINK1 KD POE SHSY5Y treated with 1 µM O/A for 3 h.

F. Quantification of Total-Tau in E for DMSO treated cells (n=3, one-way ANOVA with Dunnett's correction).

G-J. RT-qPCR validation of target gene siRNA KD vs SCR for the samples used in A-F. *KANSL1* mRNA expression (G), *KAT8* mRNA expression (H), *MAPT* mRNA expression (I), *PINK1* mRNA expression (J) (n=3, one-way ANOVA with Dunnett's correction).

Data are shown as mean ± SD.

Extended Data Figure 16 – RT-qPCR Assessments of *KANSL1* mRNA expression in control KANSL1$^{+/+}$ and heterozygous KANSL1$^{+/-}$ iNeurons, and confirmation of target gene KD in CRISPRi-i3N iNeurons

A. RT-qPCR quantification of *KANSL1* mRNA in control KANSL1$^{+/+}$ and CRISPR heterozygous KANSL1$^{+/-}$ d17 iNeurons (n=6 inductions, unpaired t-test).

B-D. RT-qPCR validation of target gene sgRNA KD vs Non-targeting sgRNA for the samples used in Figure 6 D-E. KANSL1 mRNA expression (B), KAT8 mRNA expression (C) and PINK1 mRNA expression (D) (n=3 inductions, unpaired t-test).

Data are shown as mean ± SD.

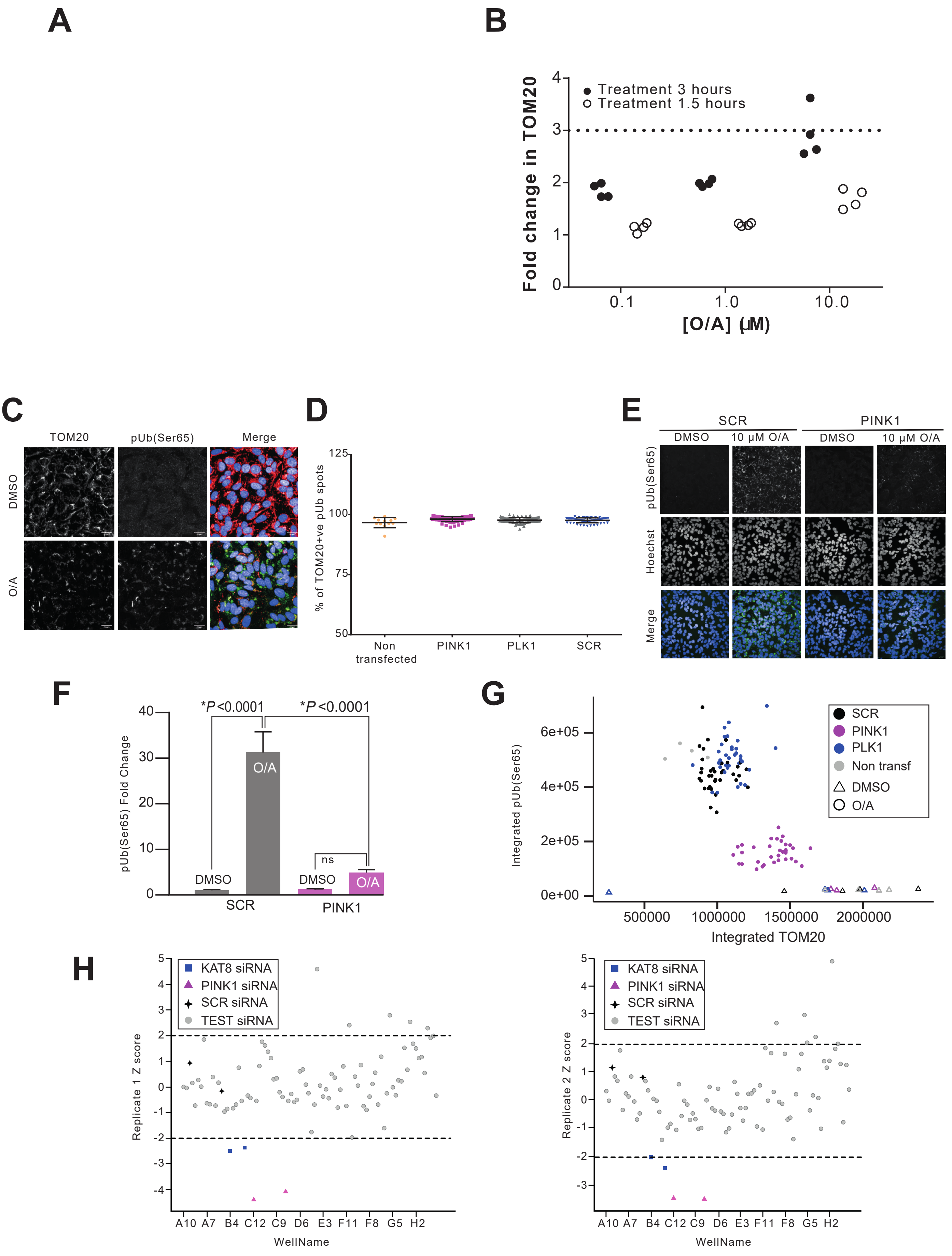

Extended Data Figure 1 - High Content siRNA Screen for modulators of pUb(Ser65)

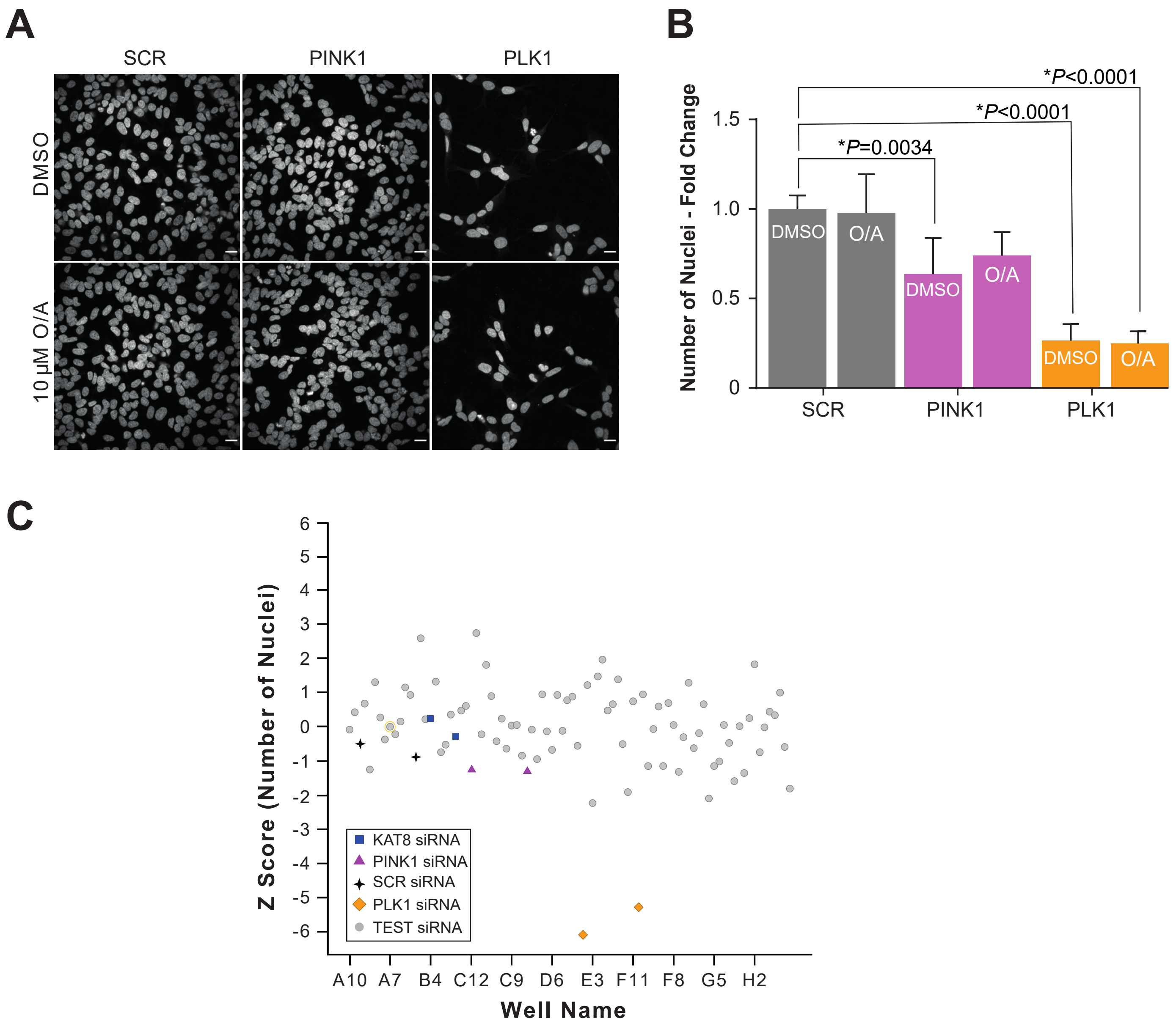

Extended Data Figure 2 - KAT8 knockdown has no effect on cell viability

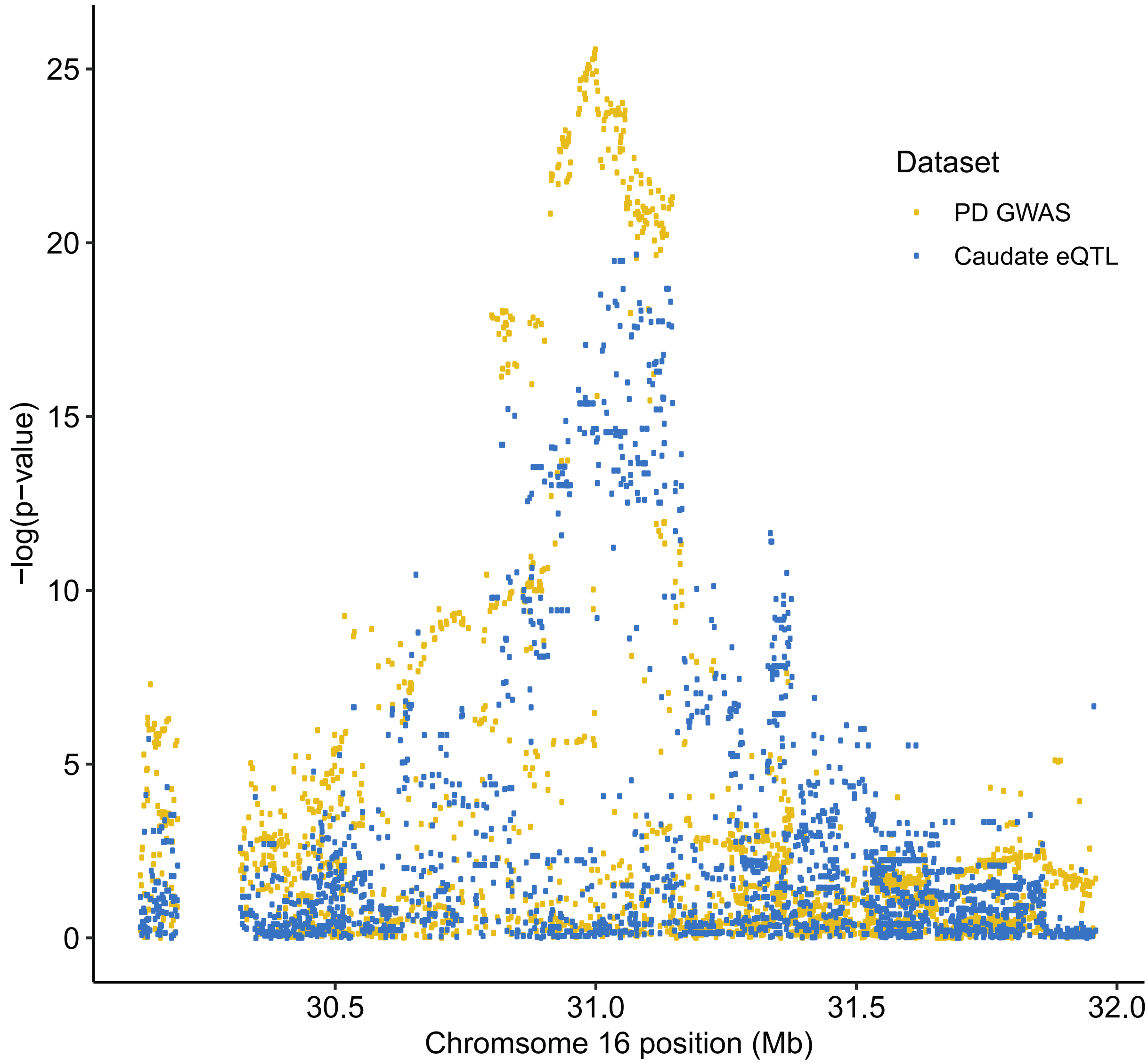

Extended Data Figure 3 - KAT8 eQTLs colocalise with SNPs associated with PD risk

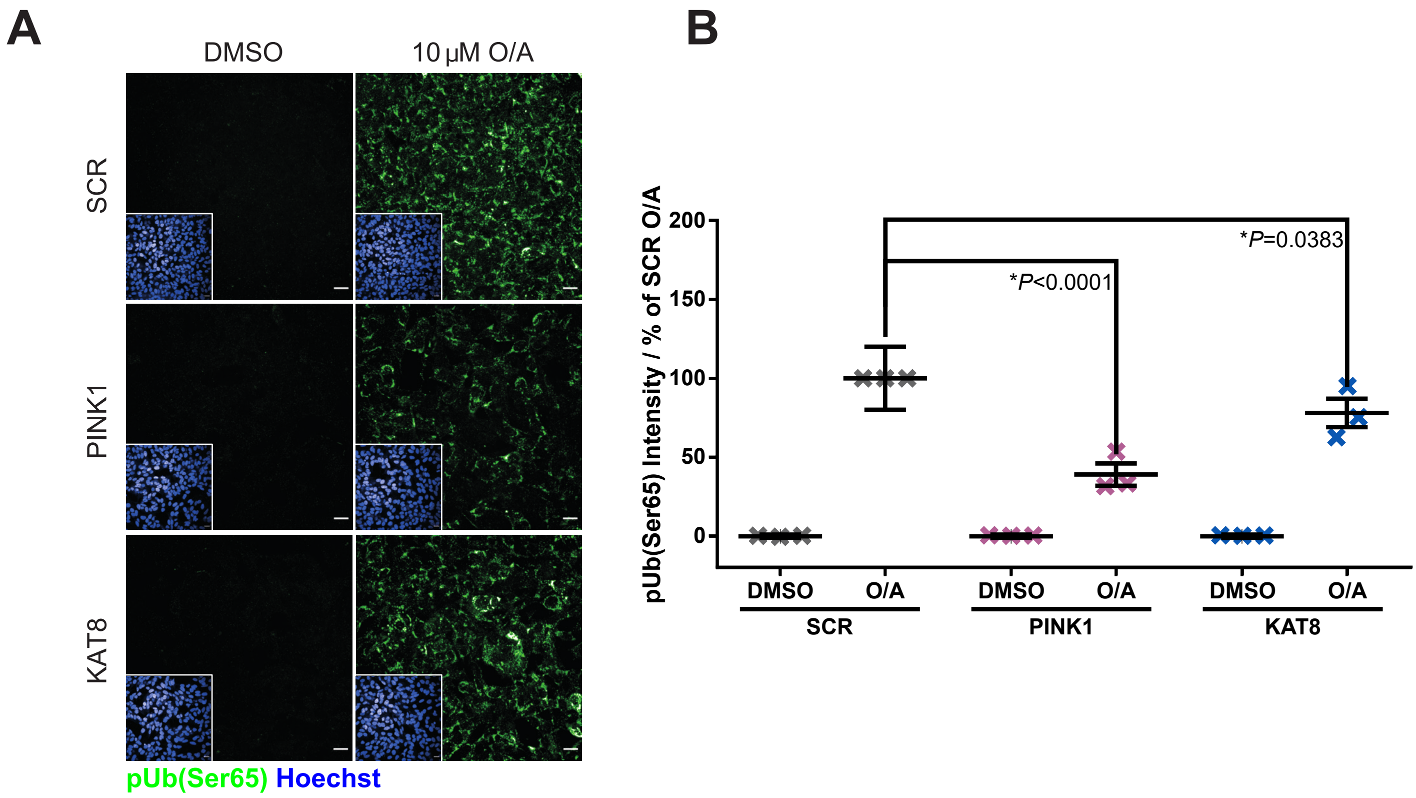

Extended Data Figure 4 - KAT8 knockdown decreases pUb(Ser65) levels

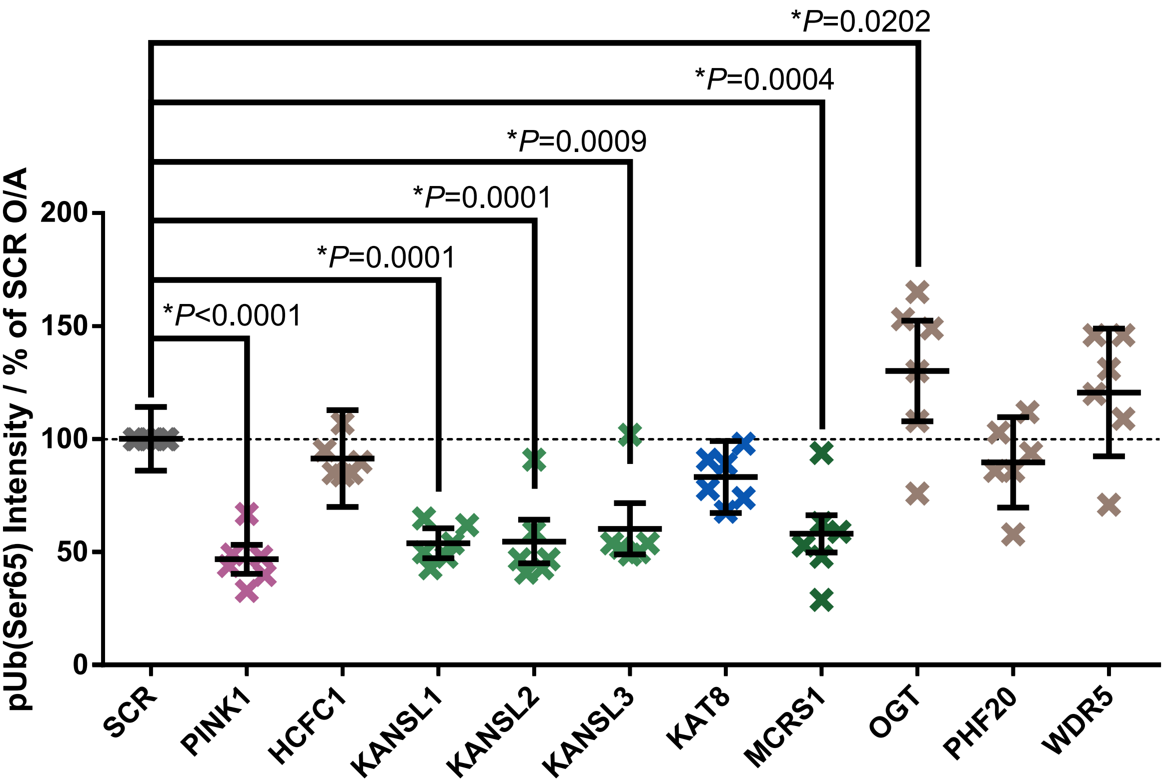

Extended Data Figure 5 - Knockdown of components of the NSL complex reduced pUb(Ser65) levels

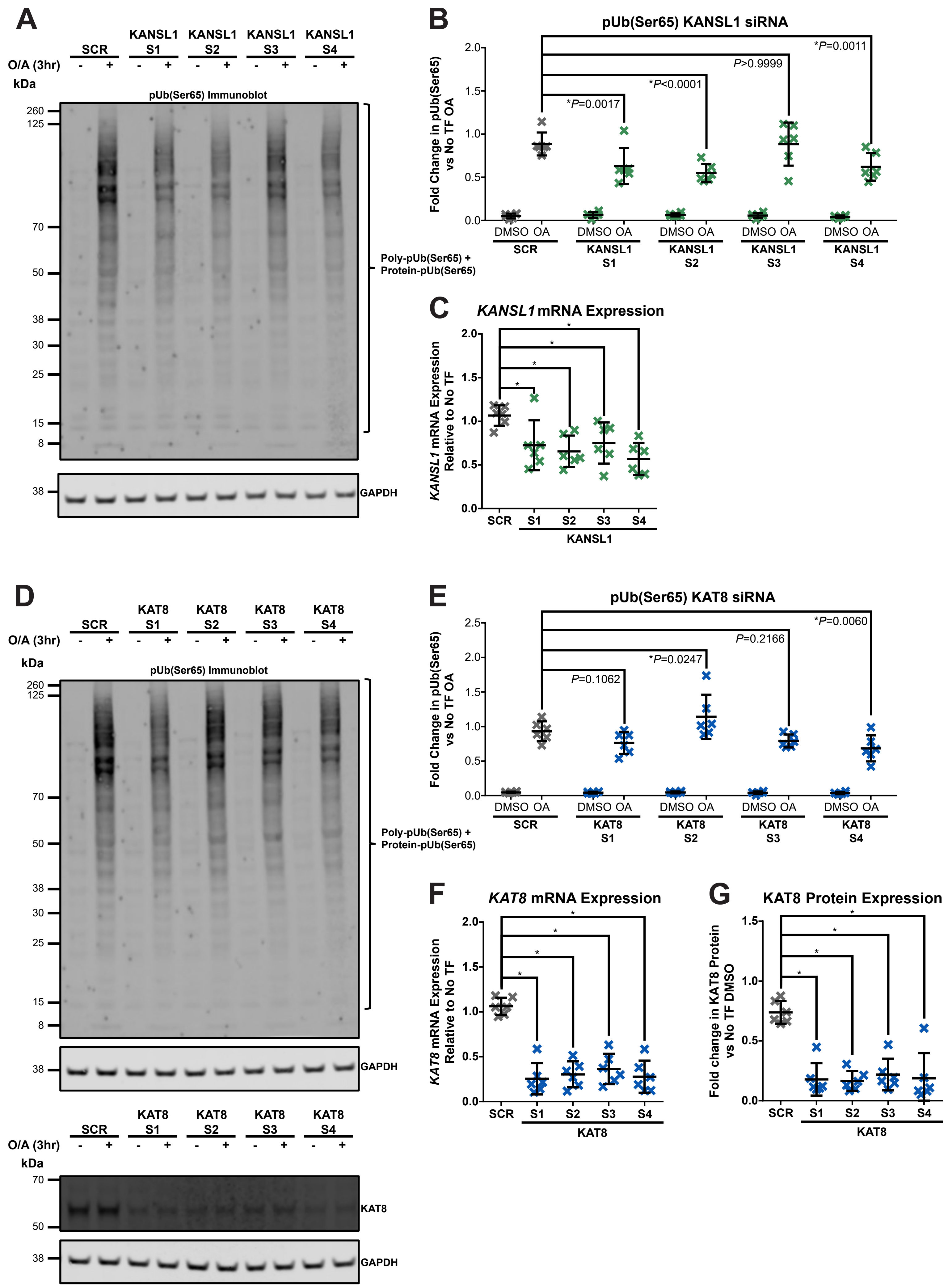

Extended Data Figure 6 - Deconvolution of the KANSL1 and KAT8 siRNA pools

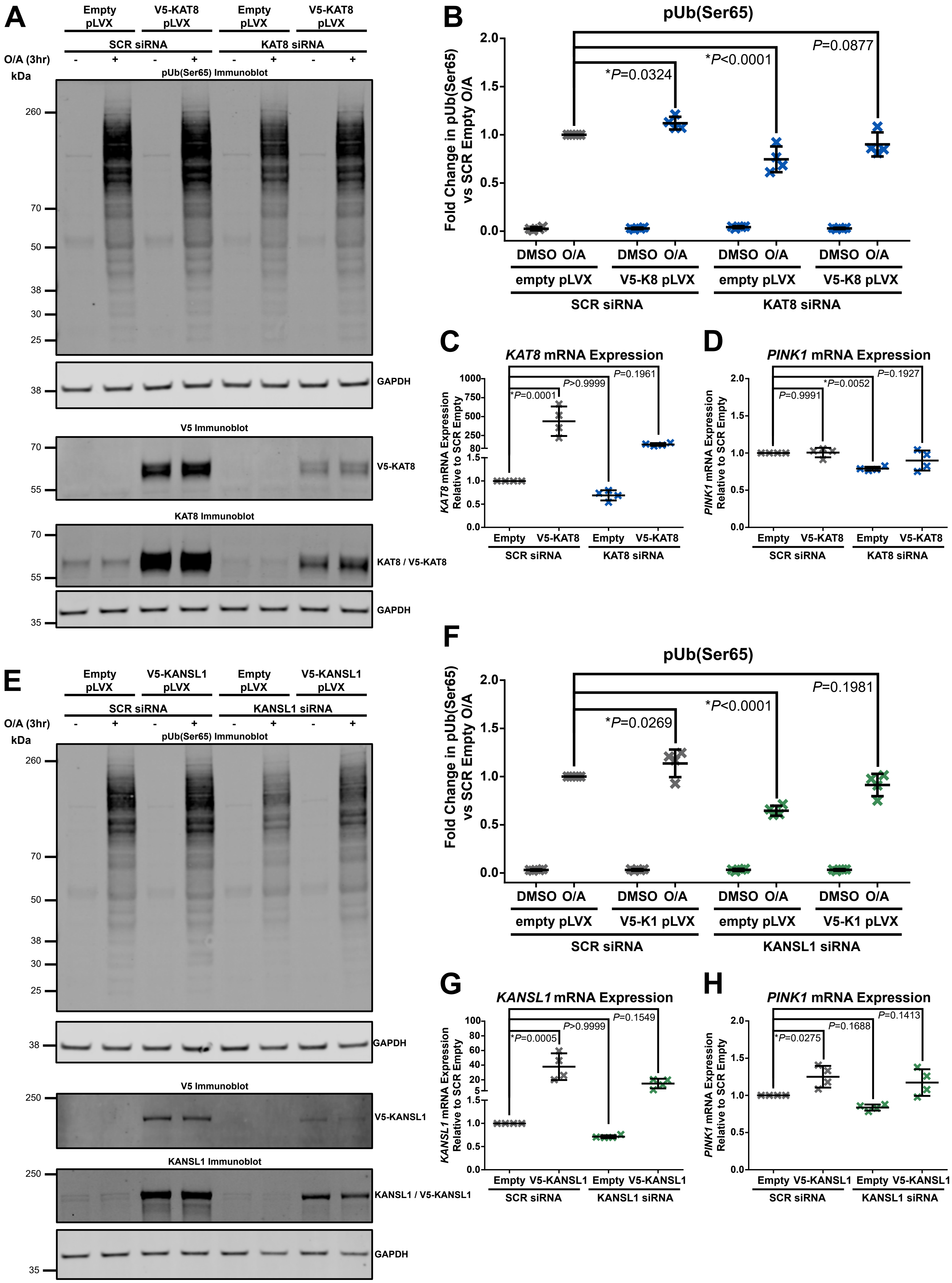

Extended Data Figure 7 - Rescue of pUb(Ser65) and PINK1 mRNA deficits in KANSL1 and KAT8 siRNA KD cells with V5-KANSL1 and V5-KAT8 overexpression

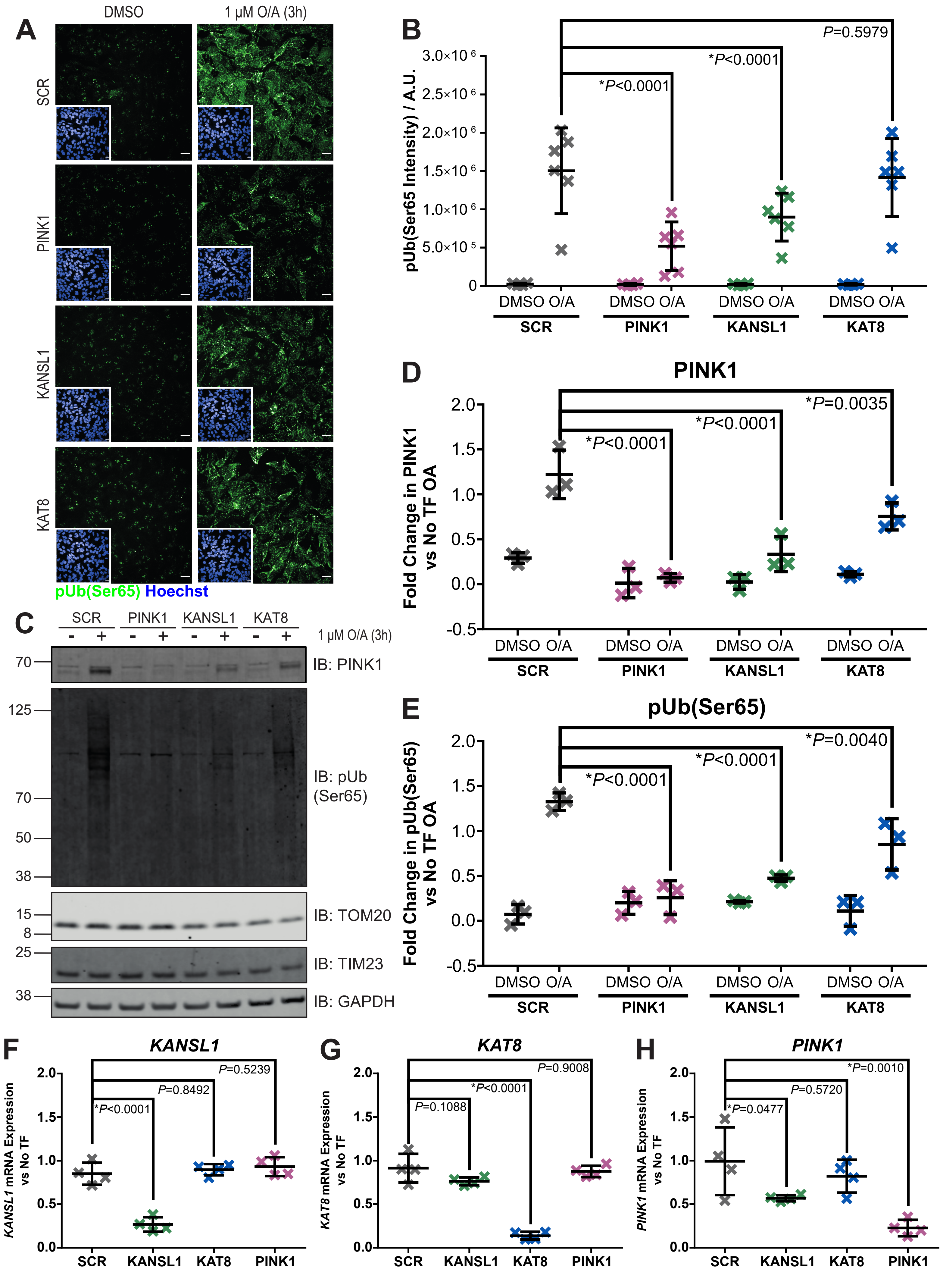

Extended Data Figure 8 - KAT8 and KANSL1 knockdown reduce pUb(Ser65) levels in WT SHSY5Y and H4 cells

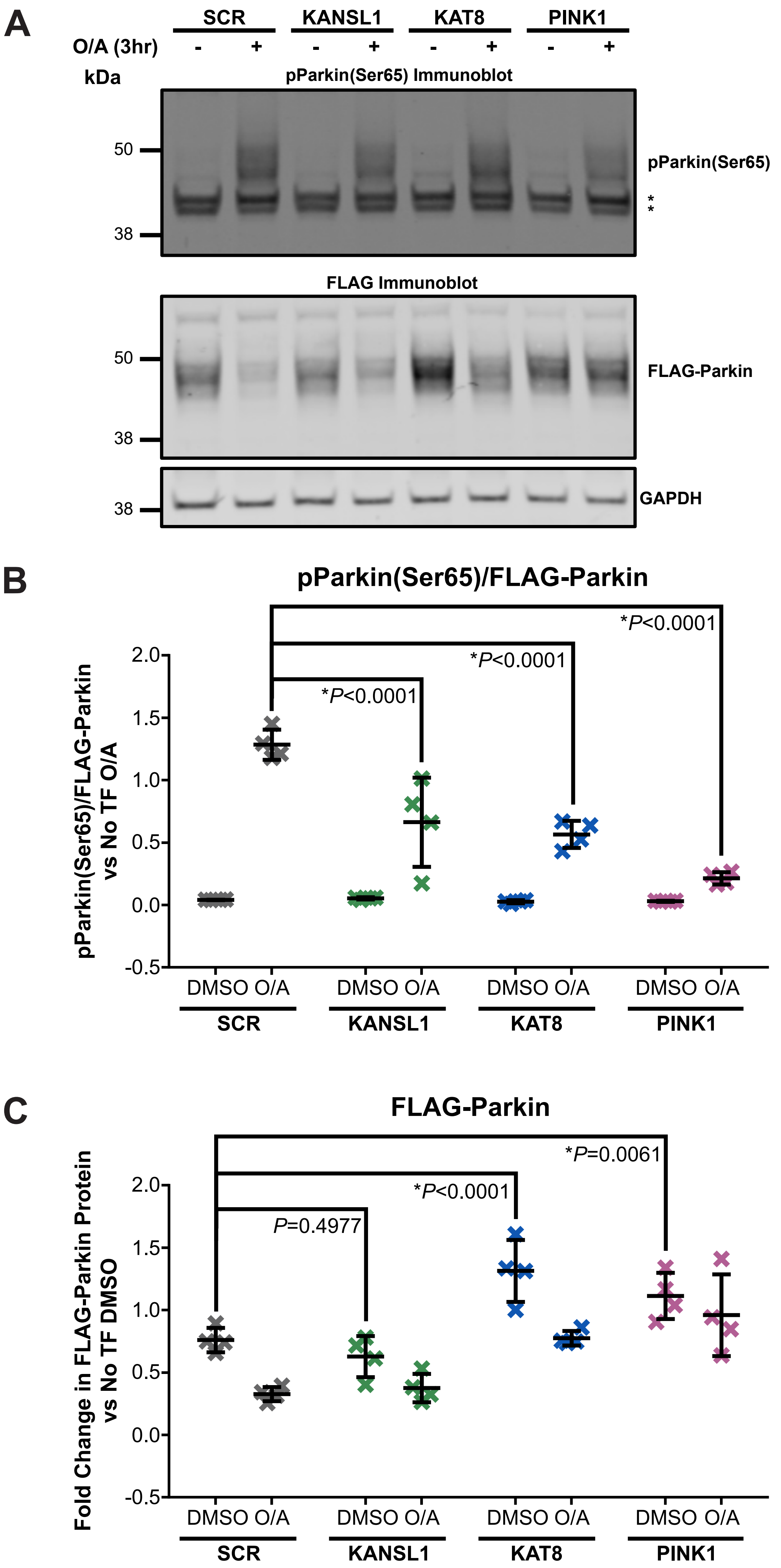

Extended Data Figure 9 - KAT8 and KANSL1 knockdown reduce PINK1-dependent phosphorylation of Parkin at Ser65, but not total FLAG-Parkin levels

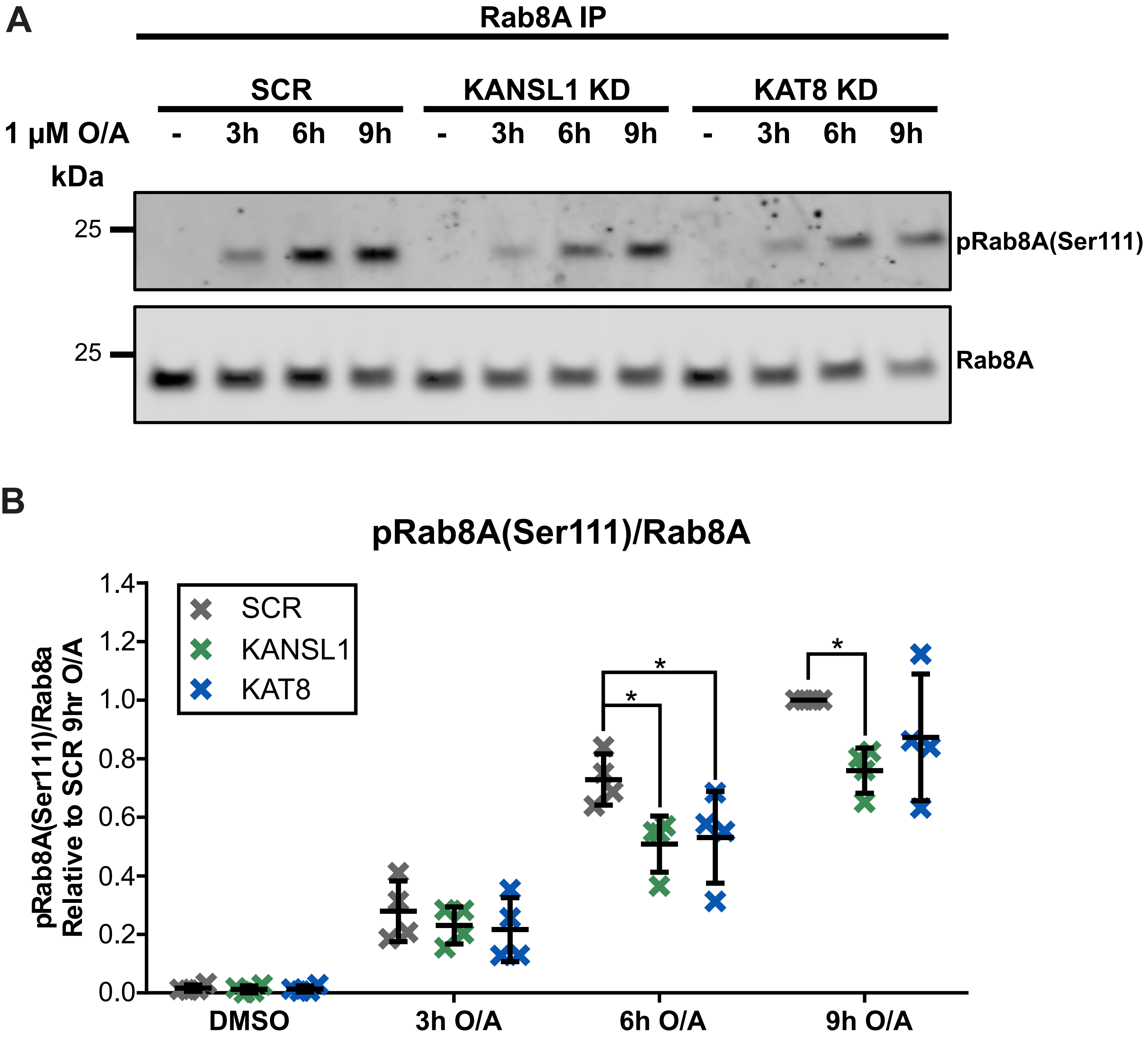

Extended Data Figure 10 - KANSL1 and KAT8 KD reduce PINK1-dependant phosphorylation of Rab8A (pRab8A(Ser111)) in PINK1 OE SHSY5Ys

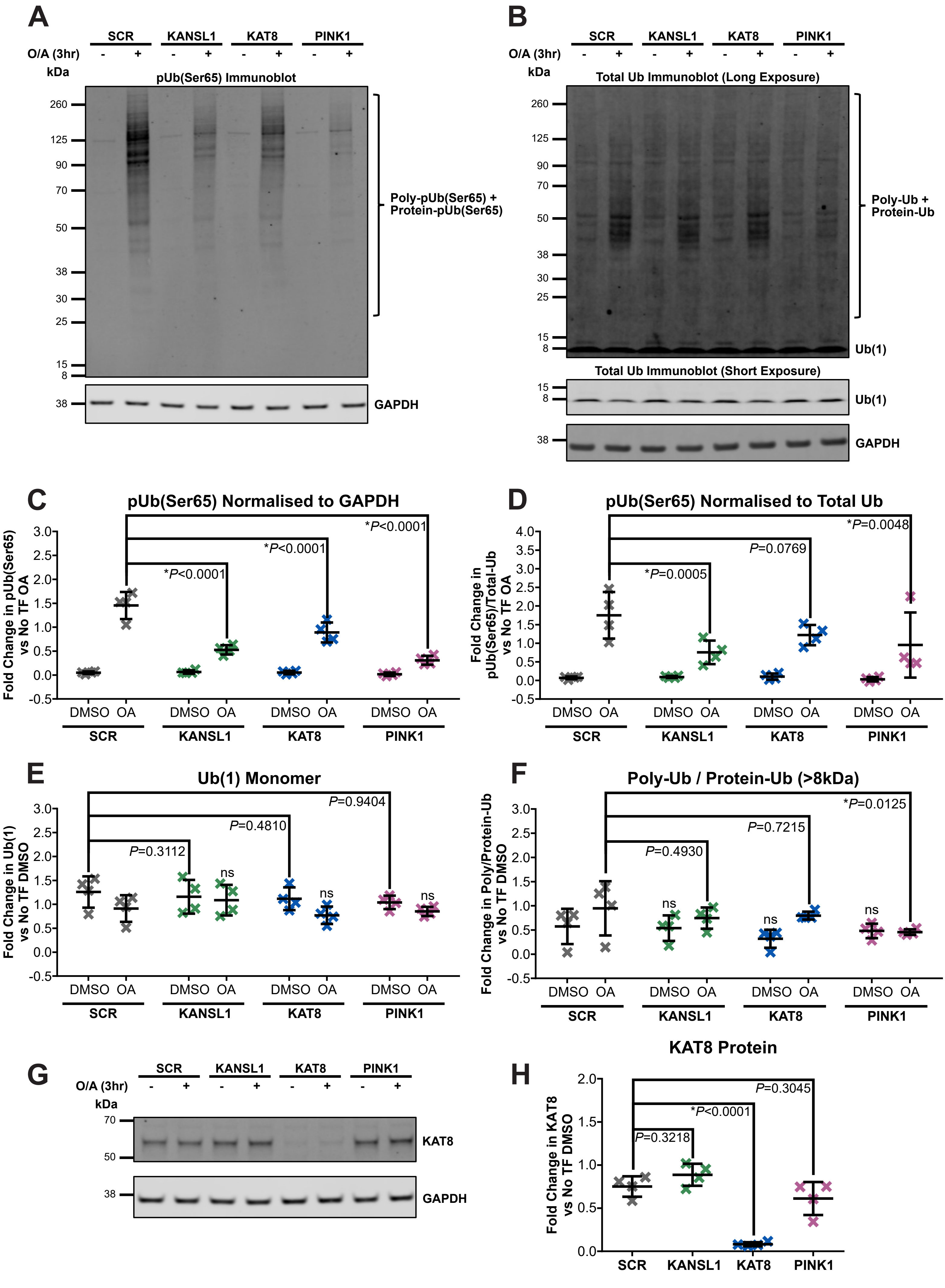

Extended Data Figure 11 - pUb(Ser65) deposition but not total-Ub availability is reduced following KANSL1 and KAT8 KD

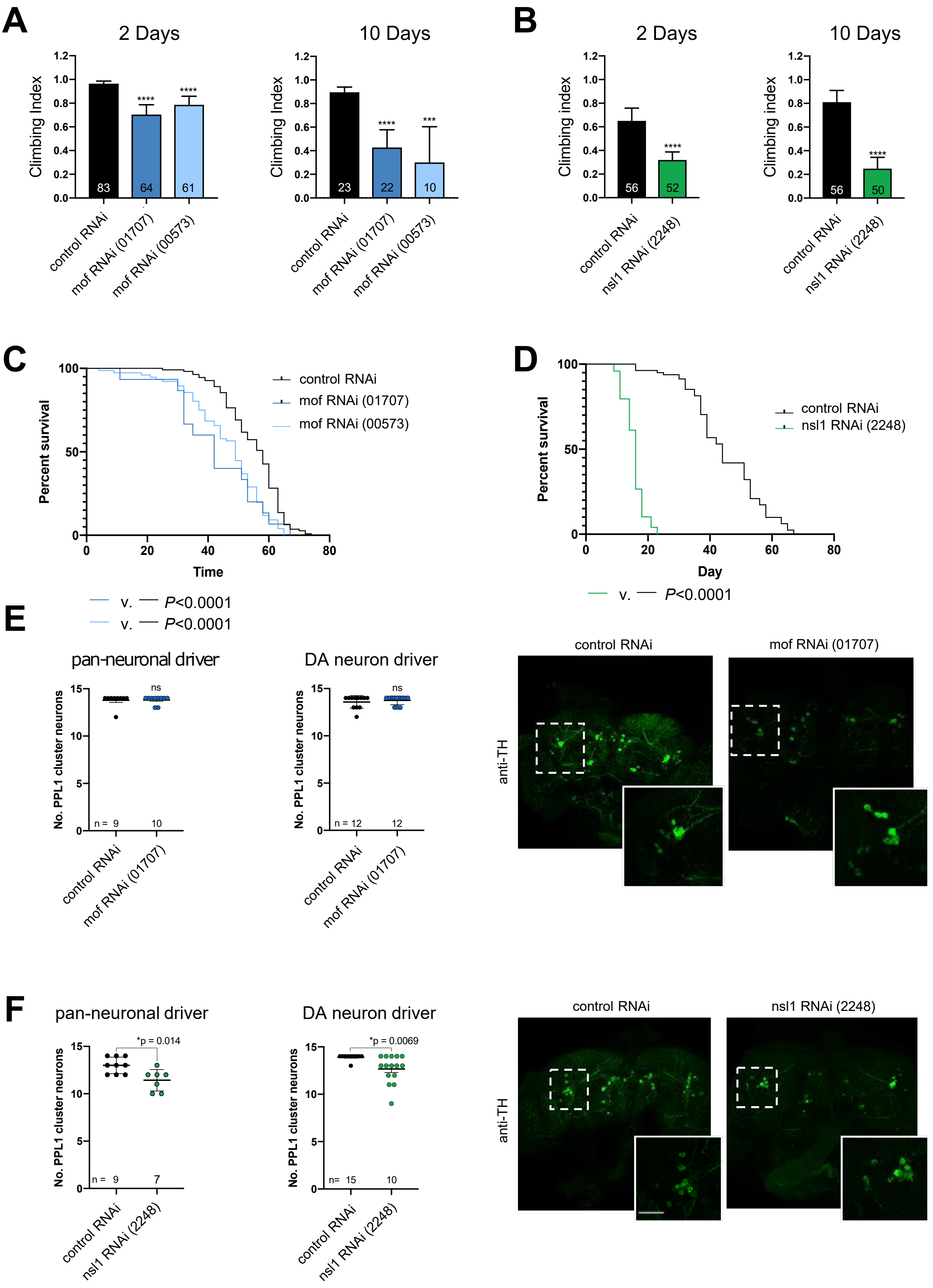

Extended Data Figure 12 - Neuronal loss of *mof* or *nsl1* causes locomotor deficit, shortened lifespan and neurodegeneration

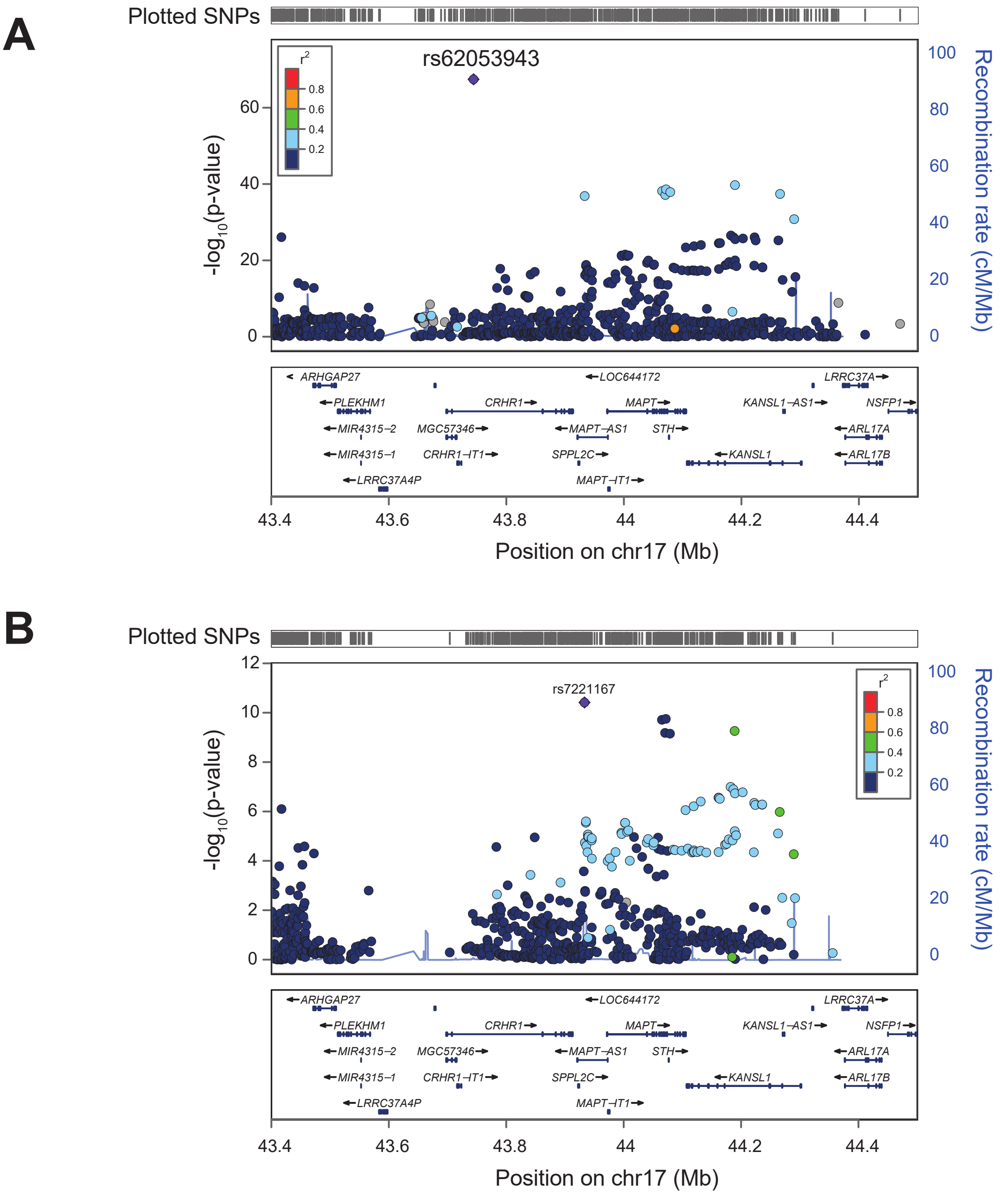

Extended Data Figure 13 - Overview of the PD GWAS genetic signal at the *MAPT* locus

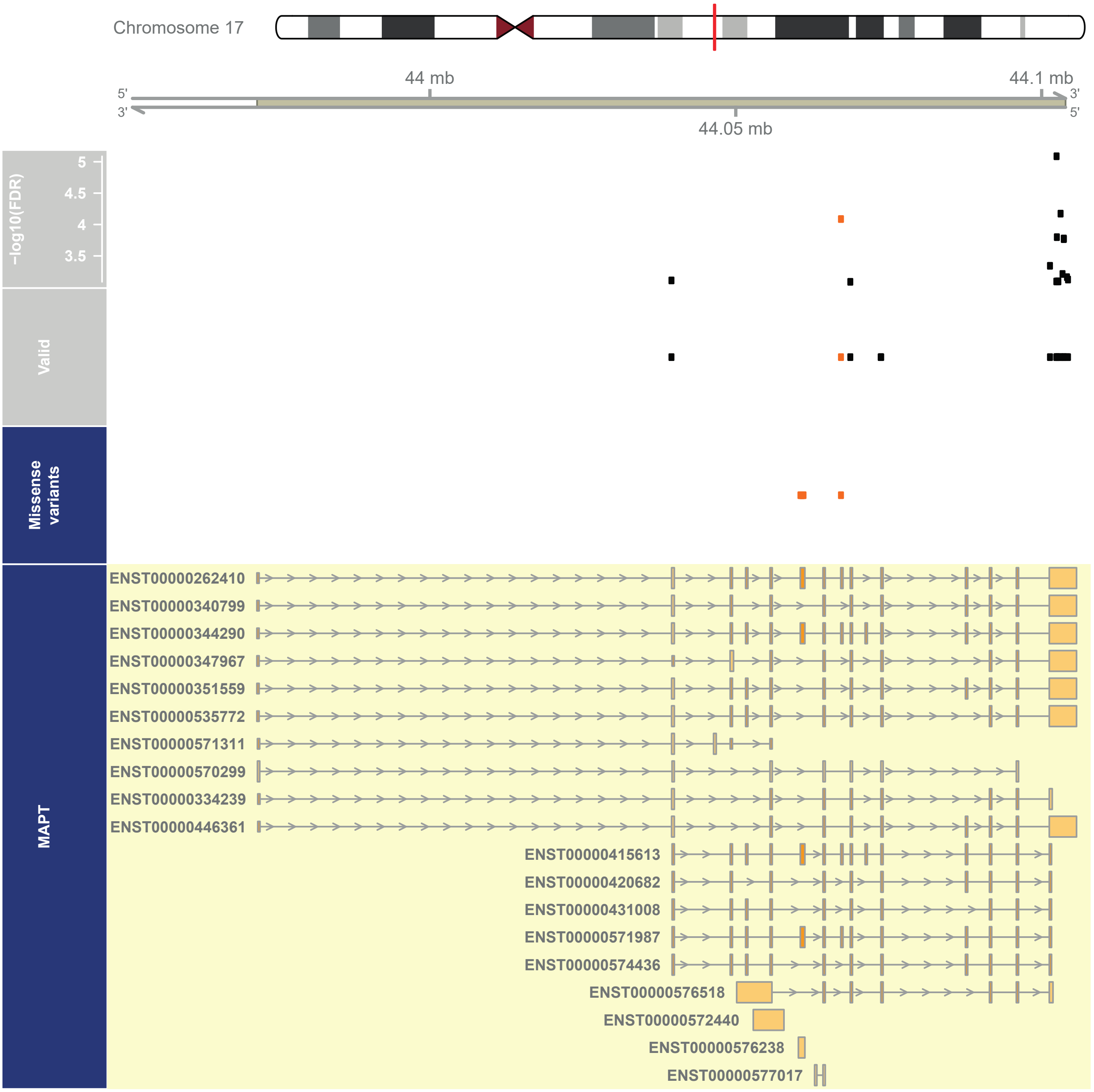

Extended Data Figure 14 - ASE sites in *MAPT* in LD with the H1/H2 SNP

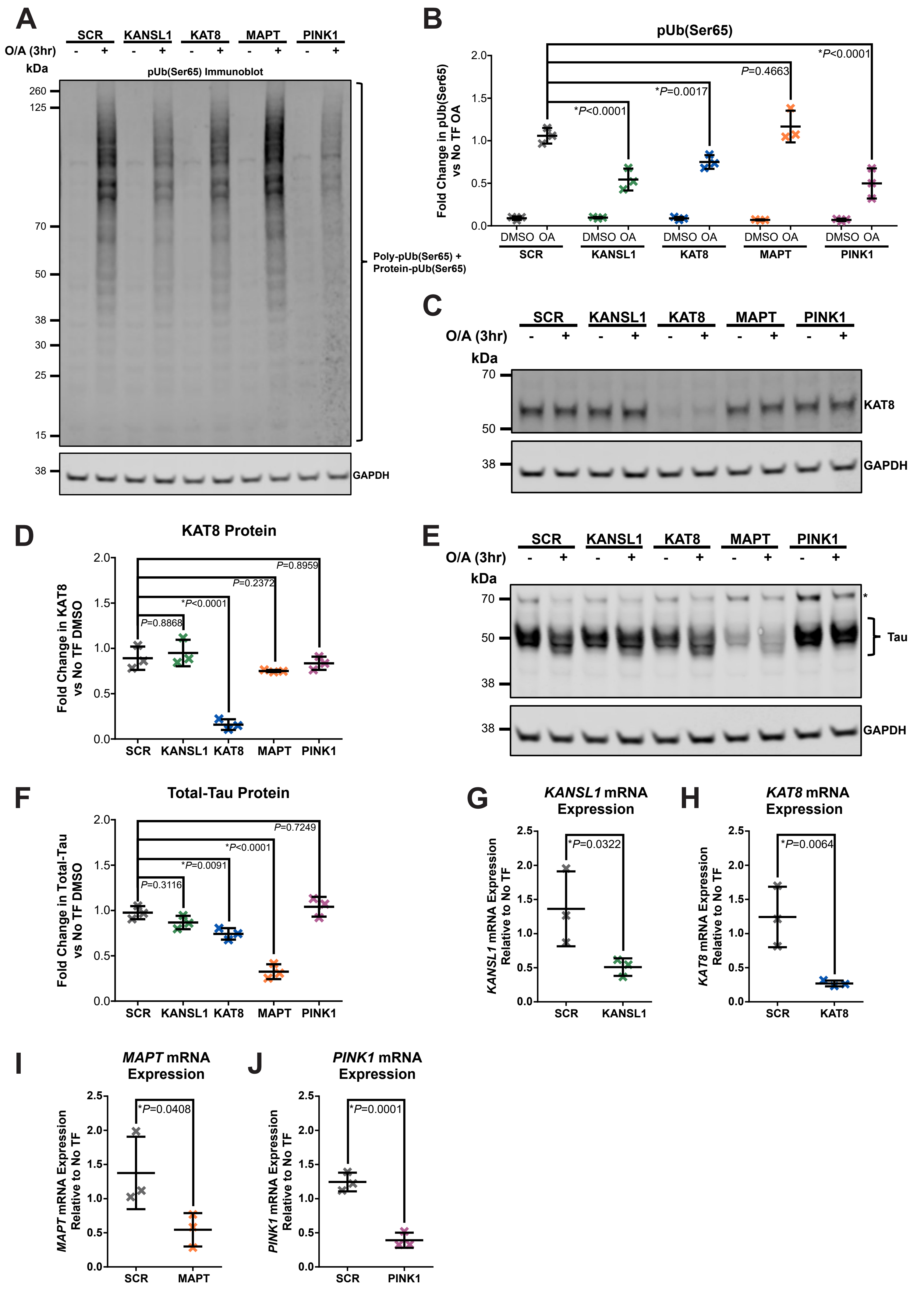

Extended Data Figure 15 - KANSL1 and KAT8 KD but not Tau KD reduce pUb(Ser65)

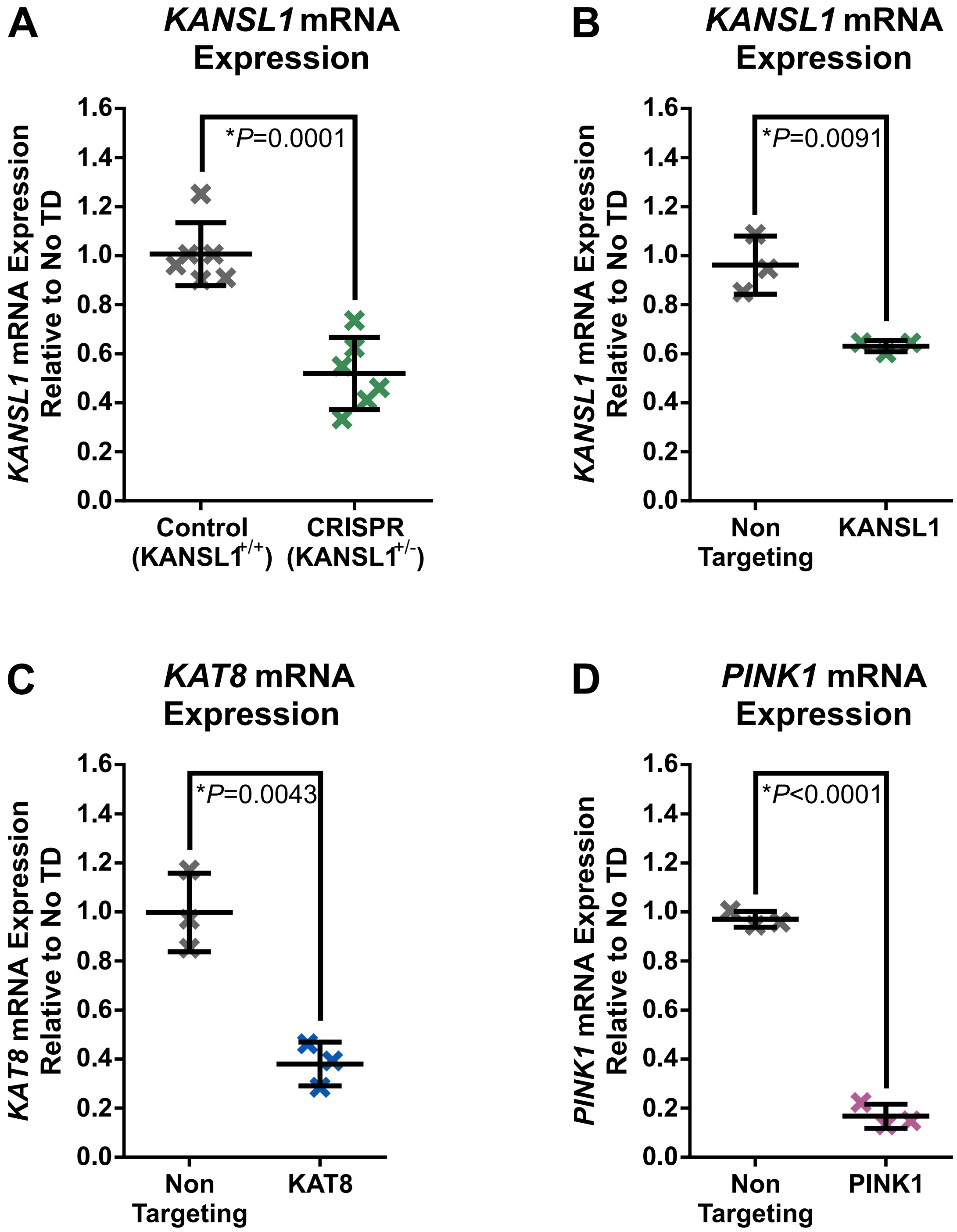

Extended Data Figure 16 - RT-qPCR Assessments of *KANSL1* mRNA expression in control KANSL1$^{+/+}$ and heterozygous KANSL1$^{+/-}$ iNeurons, and confirmation of target gene KD in CRISPRi-i3N iNeurons
