# Supplementary material for: Regulation of mitophagy by the NSL complex underlies genetic risk for Parkinson’s disease at 16q11.2 and MAPT H1 loci
Source: Brain. 2022 Sep 8;145(12):4349–67. doi: 10.1093/brain/awac325 (PMC9762952; doi:10.1093/brain/awac325)

TIM23 IB: Figure 1D

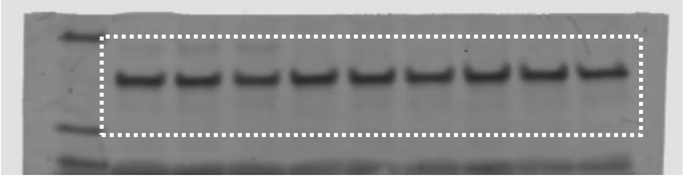

pUb(Ser65) IB: Figure 1D

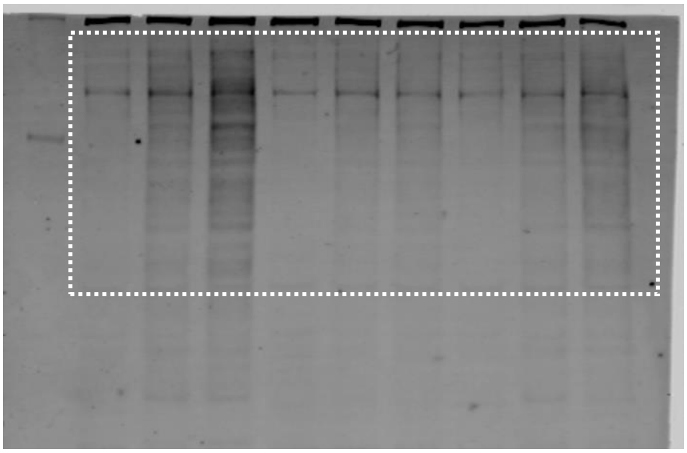

PINK1 IB: Figure 1D

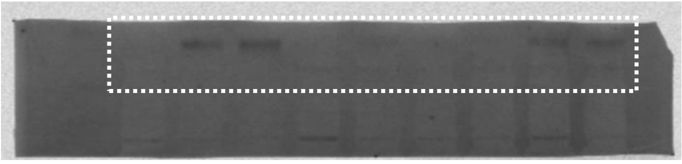

KAT8 IB: Figure 1D

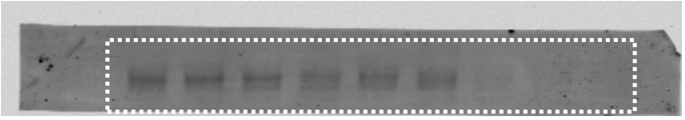

GAPDH IB: Figure 1D

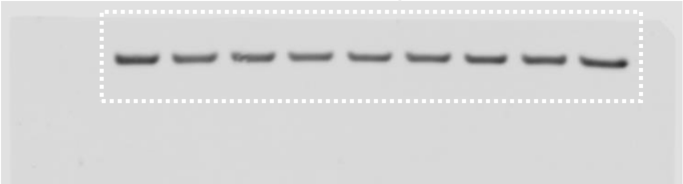

TIM23 IB: Figure 2D

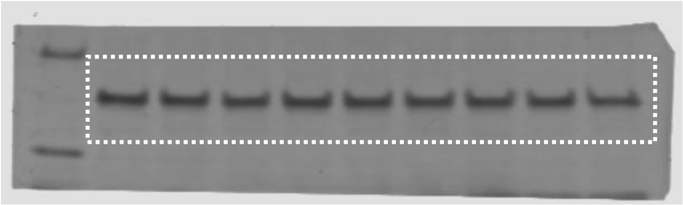

pUb(Ser65) IB: Figure 2D

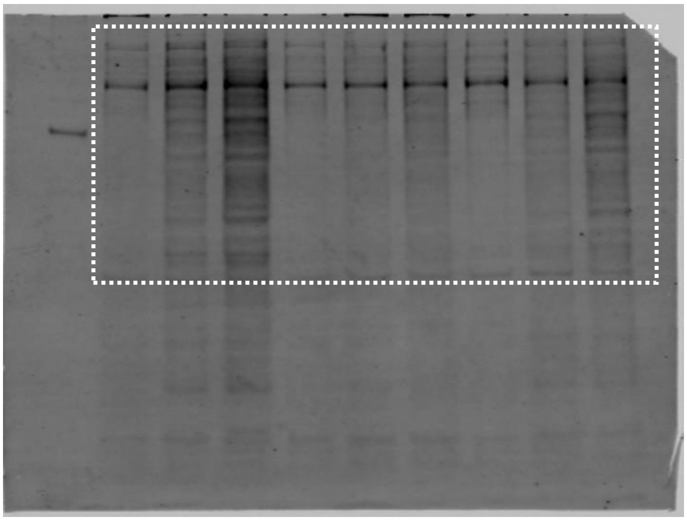

PINK1 IB: Figure 2D

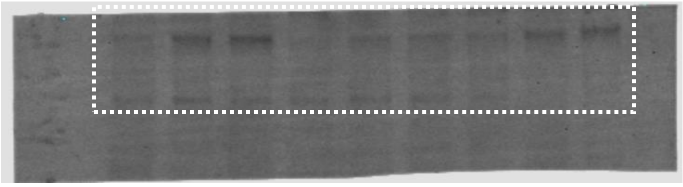

pUb(Ser65) IB: Figure 7A

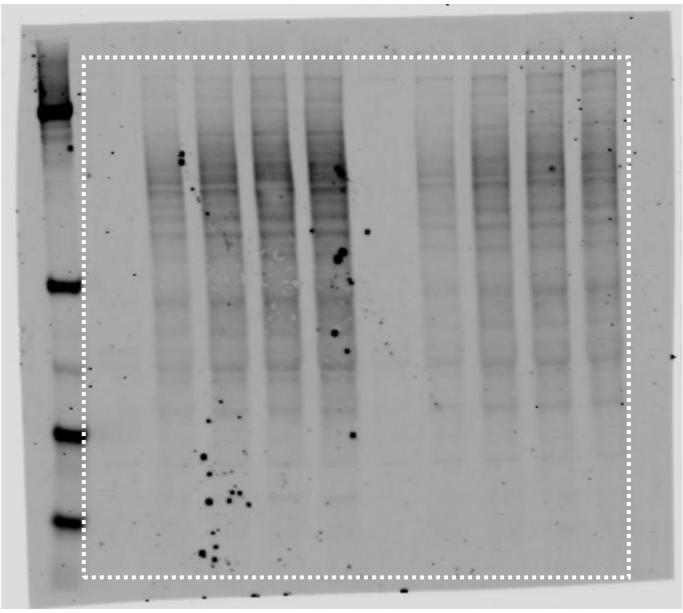

TOM20 IB: Figure 7A

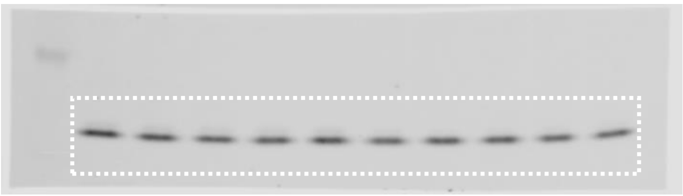

TIM23 IB: Figure 7A

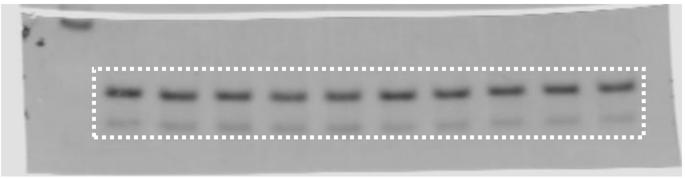

GAPDH IB: Figure 7A

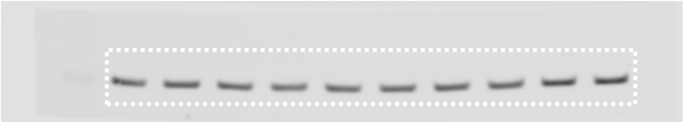

pUb(Ser65) IB: Extended Data Figure 6A

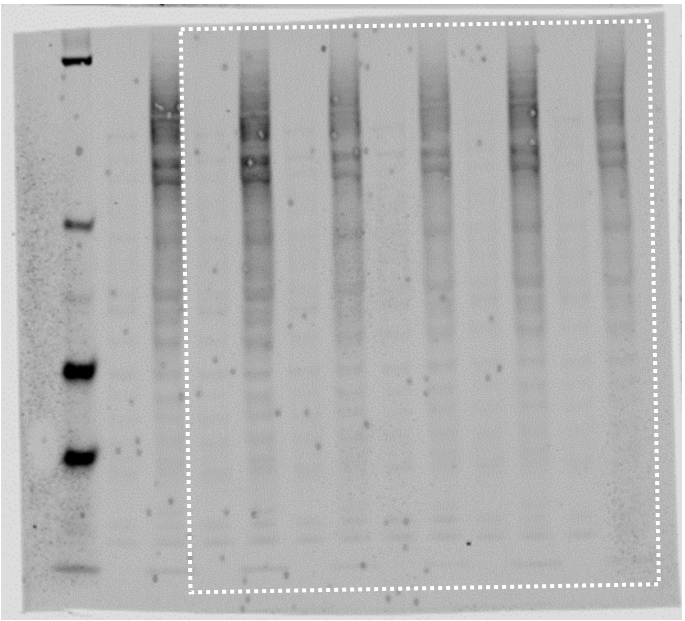

GAPDH IB for pUb(Ser65) IB: Extended Data Figure 6A

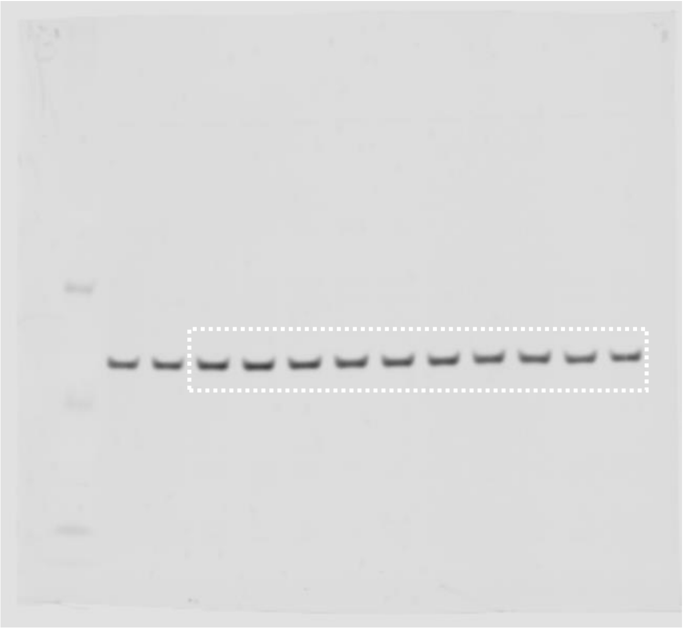

pUb(Ser65) IB: Extended Data Figure 6D

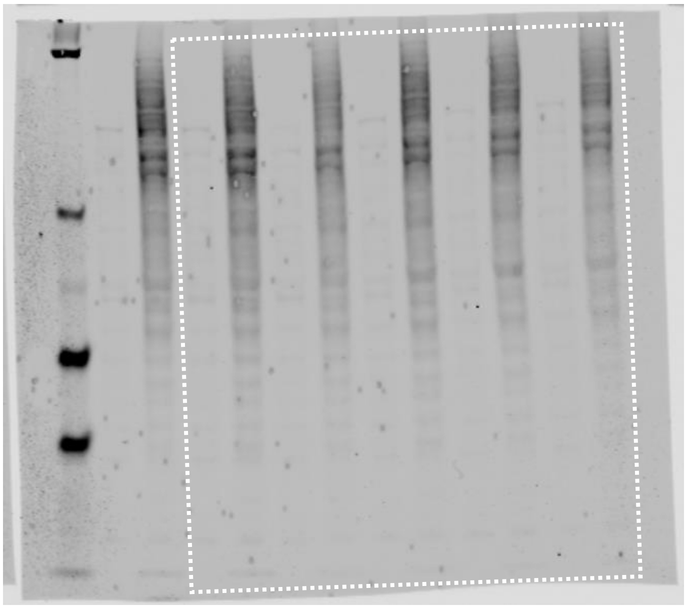

GAPDH IB for pUb(Ser65) IB: Extended Data Figure 6D

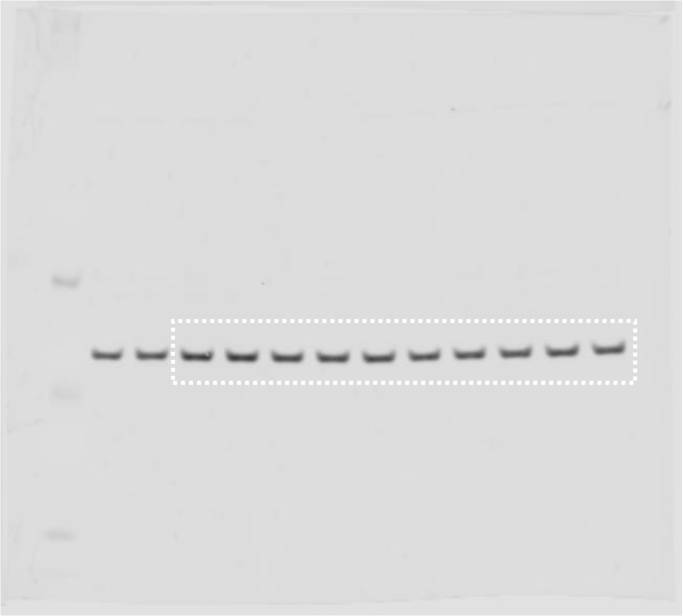

KAT8 IB: Extended Data Figure 6D

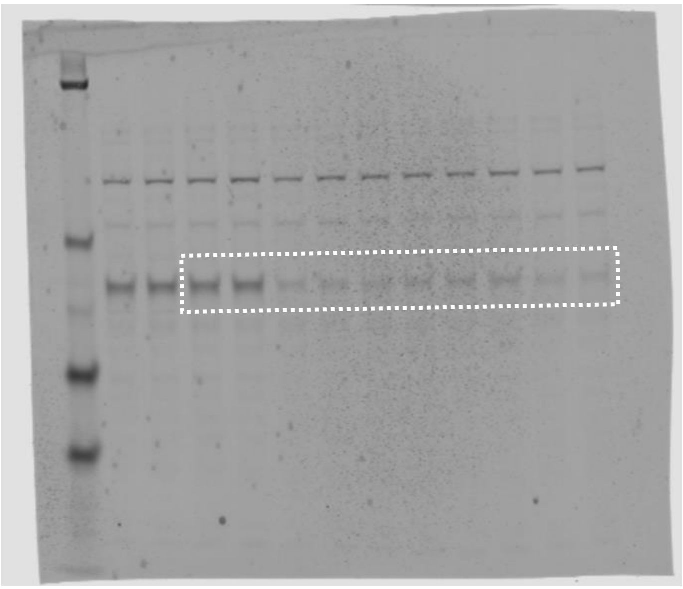

GAPDH IB for KAT8 IB: Extended Data Figure 6D

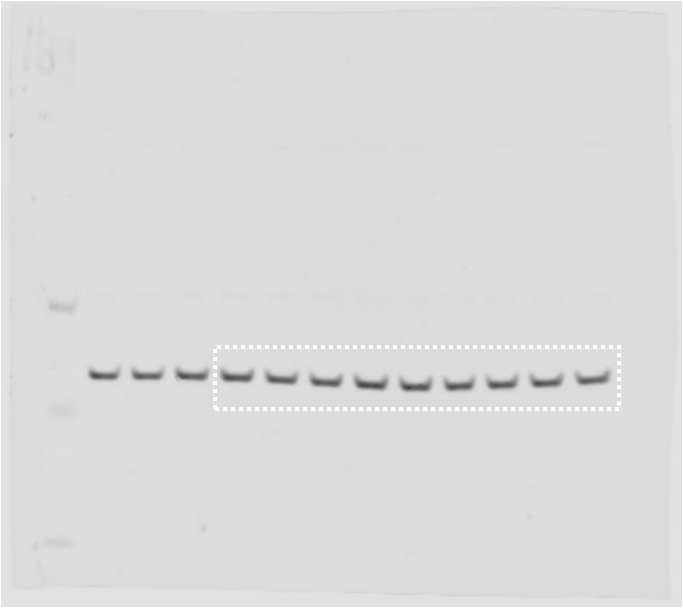

pUb(Ser65) IB: Extended Data Figure 7A

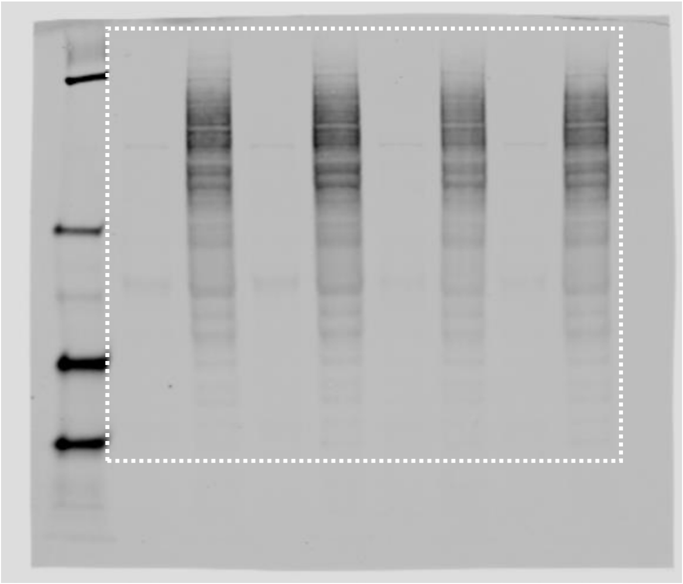

GAPDH IB for pUb(Ser65) IB: Extended Data Figure 7A

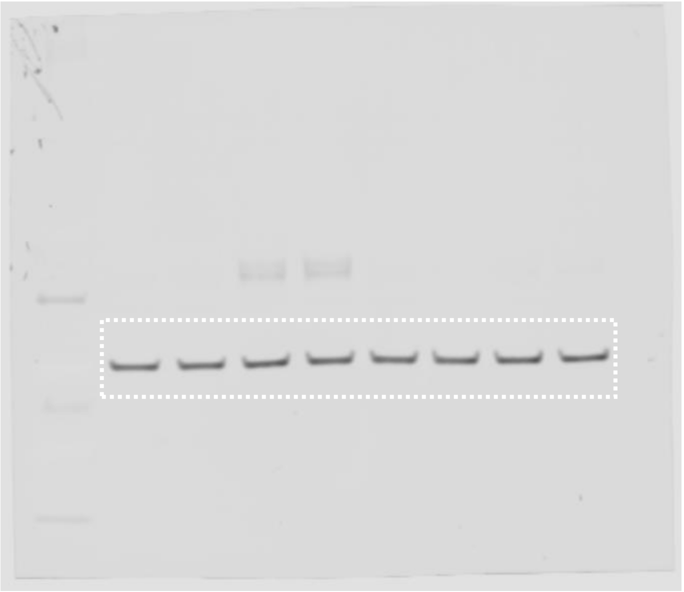

V5 Tag IB: Extended Data Figure 7A

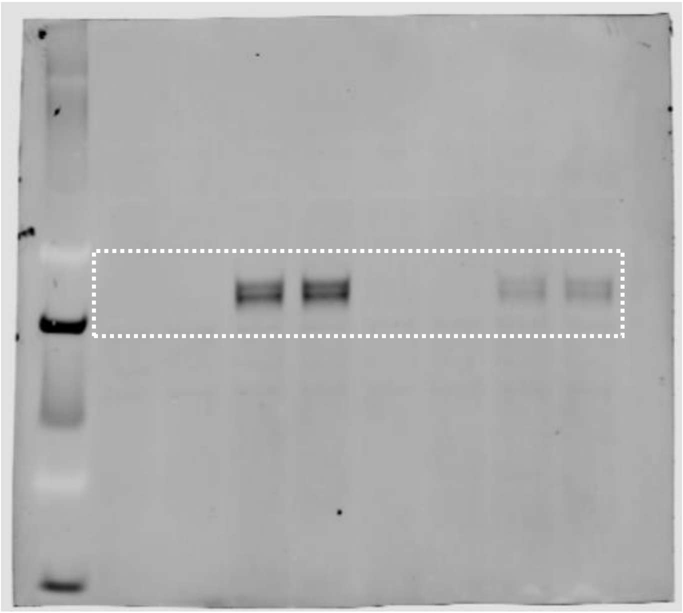

KAT8 IB: Extended Data Figure 7A

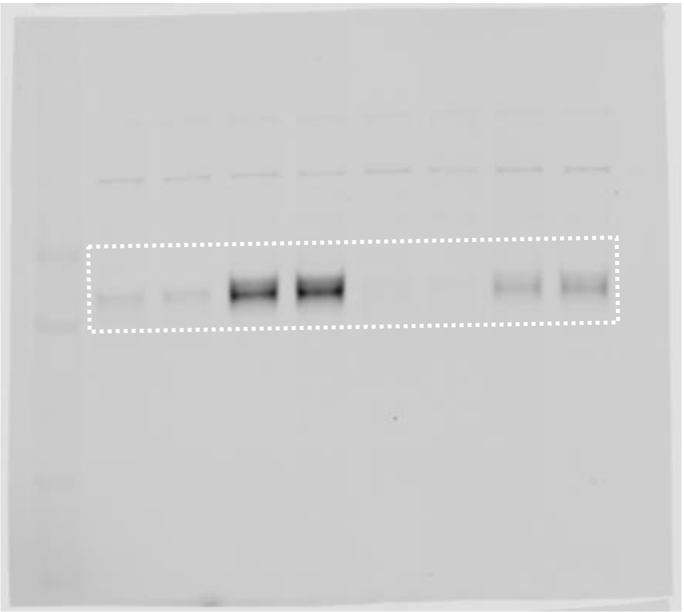

GAPDH IB for V5 + KAT8 IBs:
Extended Data Figure 7A

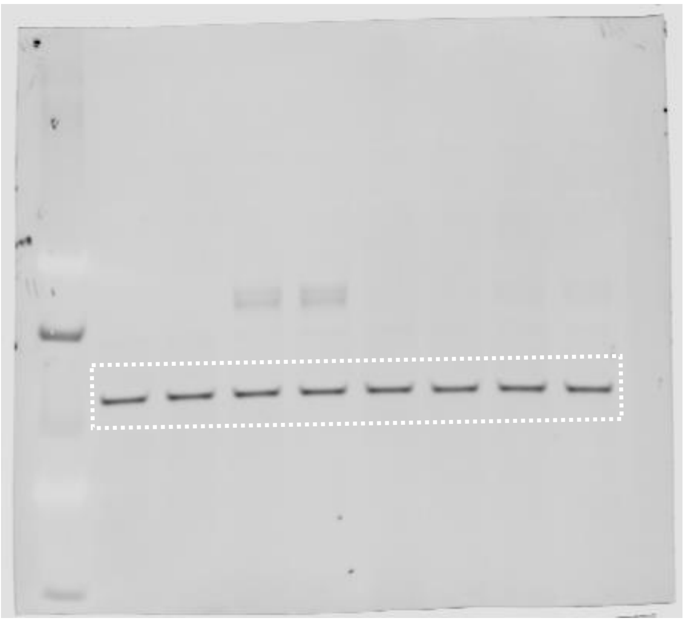

pUb(Ser65) IB: Extended Data Figure 7E

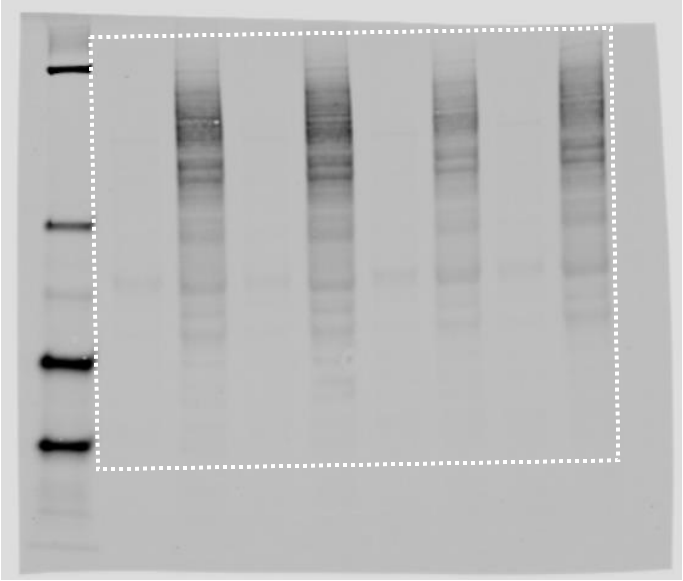

GAPDH IB for pUb(Ser65) IB: Extended Data Figure 7E

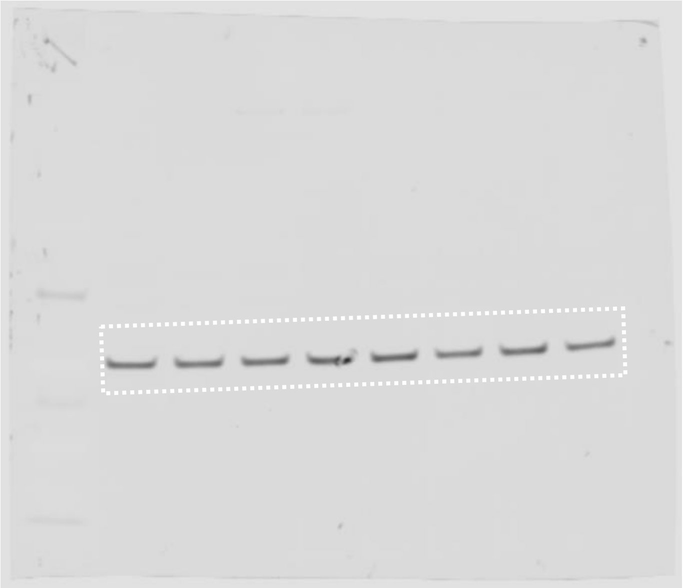

V5 Tag IB: Extended Data Figure 7E

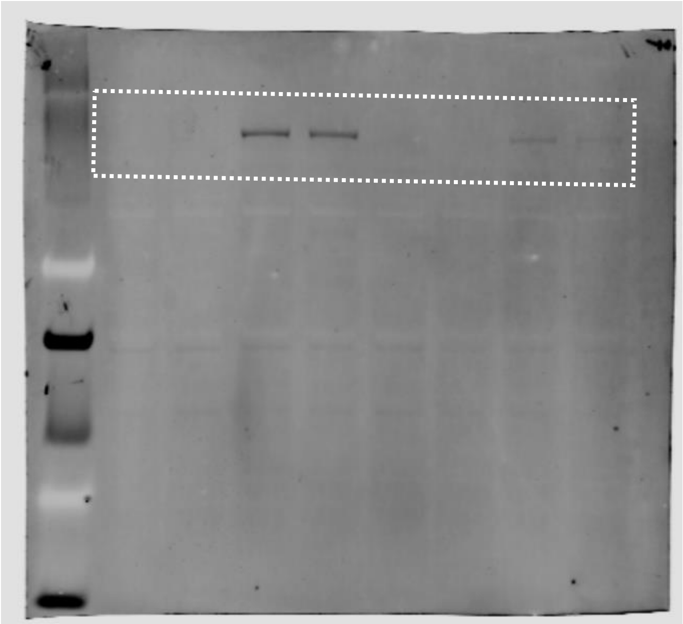

KANSL1 IB: Extended Data Figure 7E

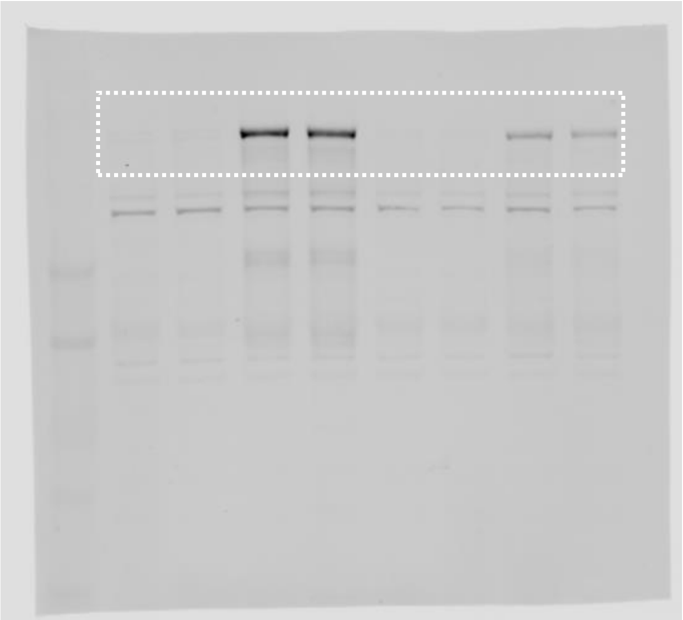

GAPDH IB for V5 + KANSL1 IBs:
Extended Data Figure 7E

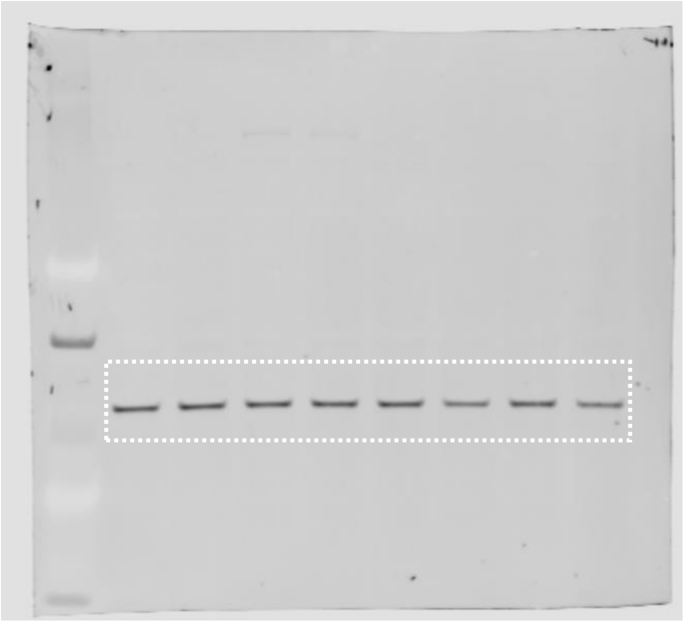

pUb(Ser65) IB: Extended Data Figure 8C

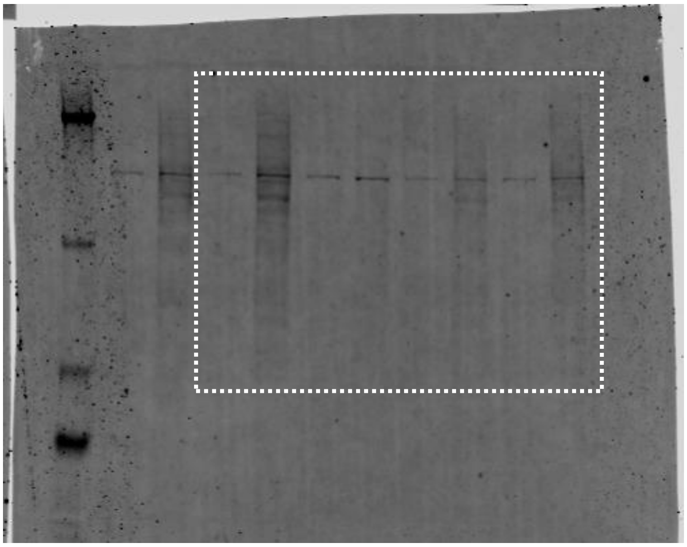

PINK1 (+MFN2) IB: Extended Data Figure 8C

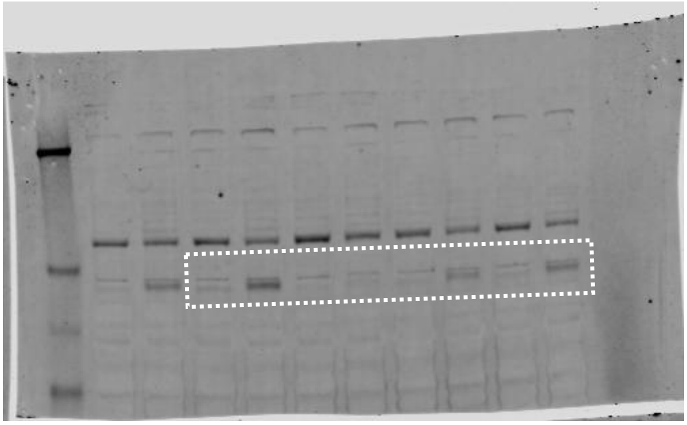

GAPDH IB: Extended Data Figure 8C

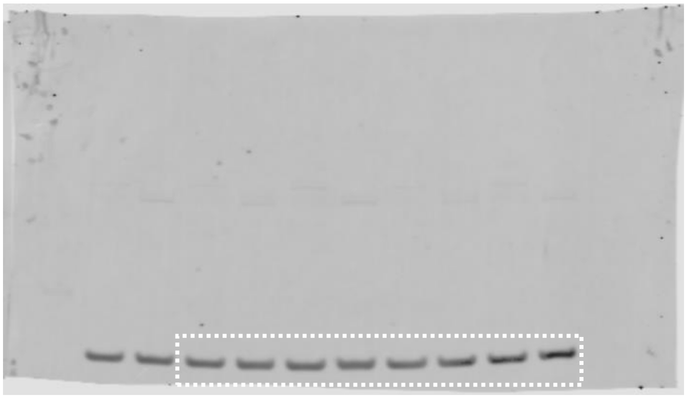

TOM20 IB: Extended Data Figure 8C

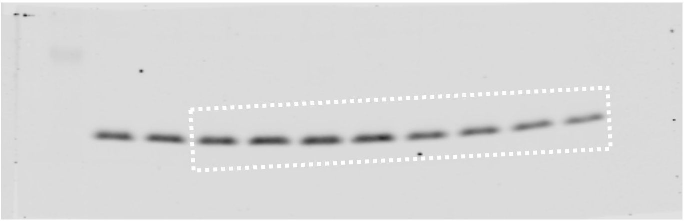

TIM23 IB: Extended Data Figure 8C

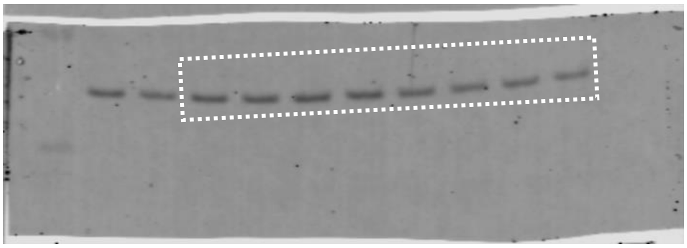

pParkin(Ser65) IB: Extended Data Figure 9A

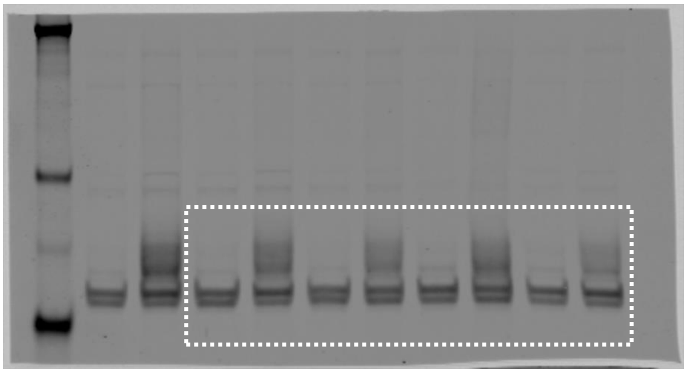

FLAG tag IB: Extended Data Figure 9A

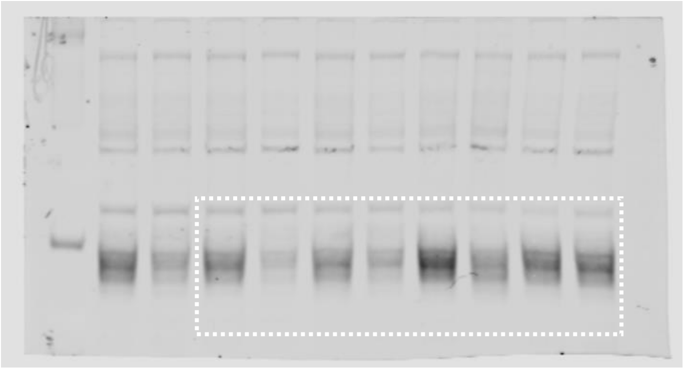

GAPDH (+FLAG tag) IB: Extended Data Figure 9A

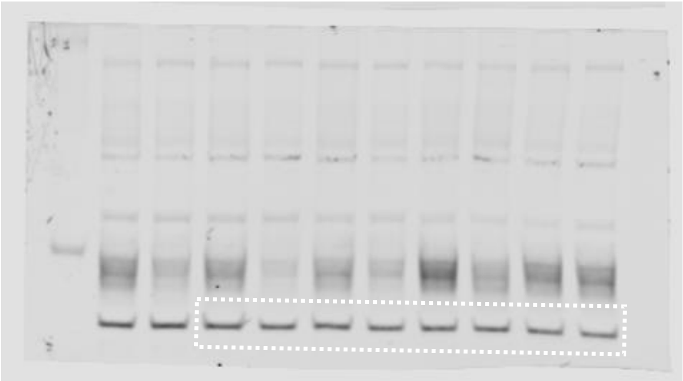

pRab8A(Ser111) IB: Extended Data Figure 10A

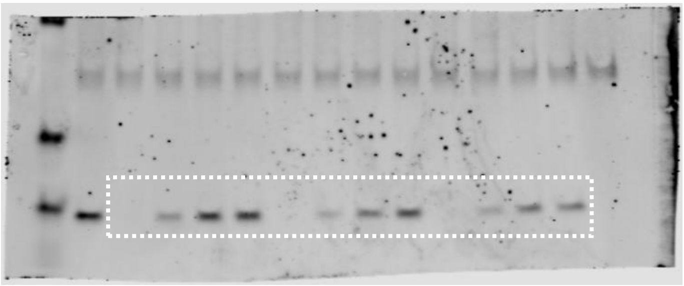

Rab8A IB: Extended Data Figure 10A

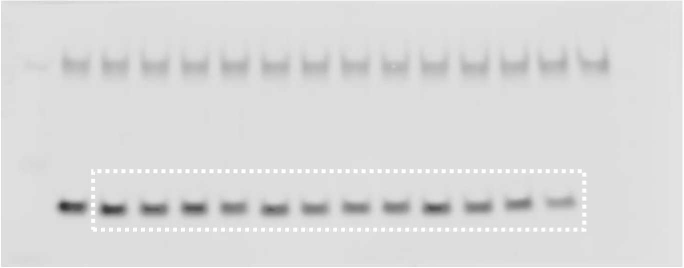

pUb(Ser65) IB: Extended Data Figure 11A

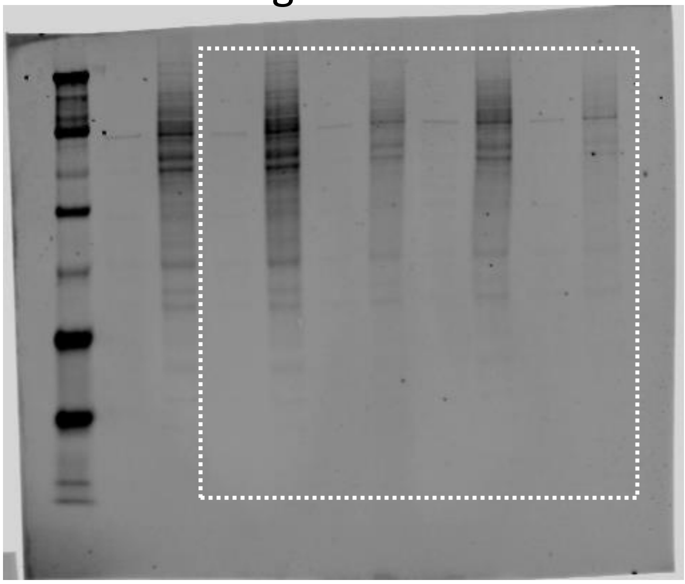

GAPDH IB for pUb(Ser65) IB: Extended Data Figure 11A

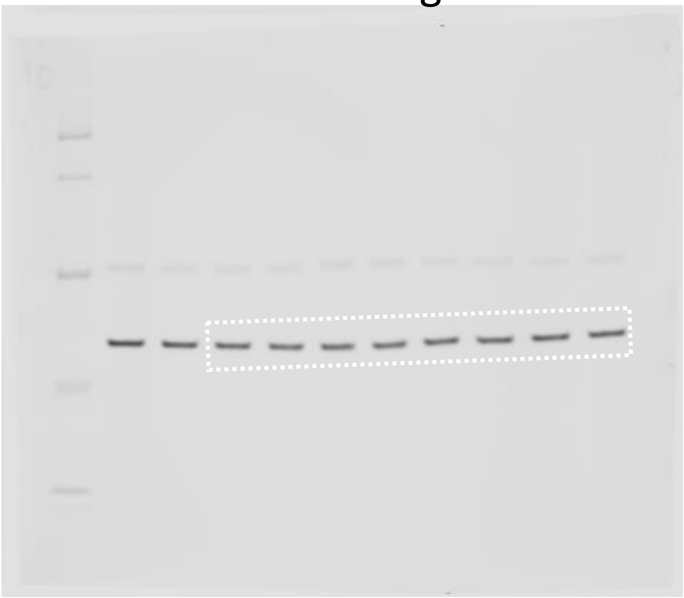

Total Ub IB (Short Exposure): Extended Data Figure 11B

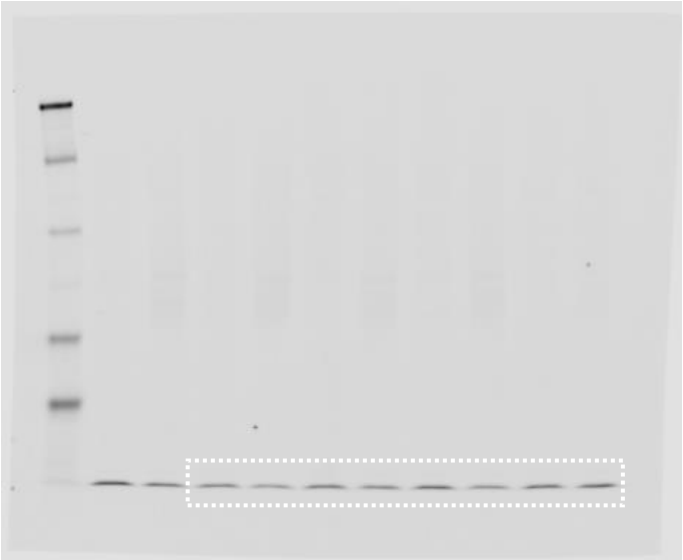

Total Ub IB (Long Exposure): Extended Data Figure 11B

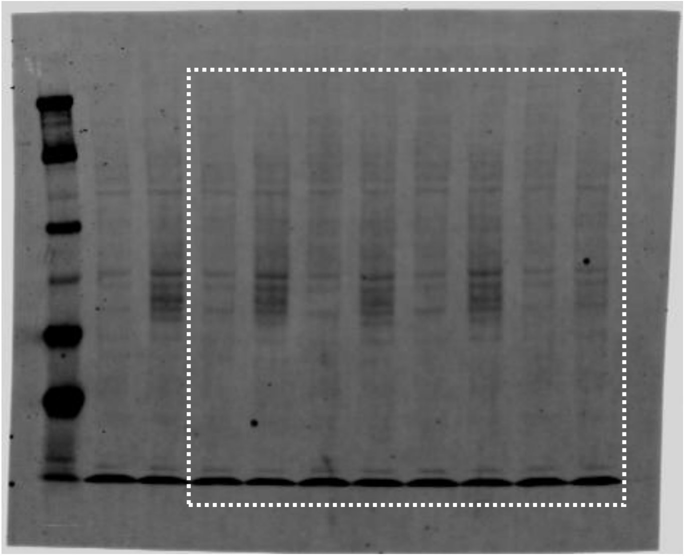

GAPDH IB for total Ub IB: Extended Data Figure 11B

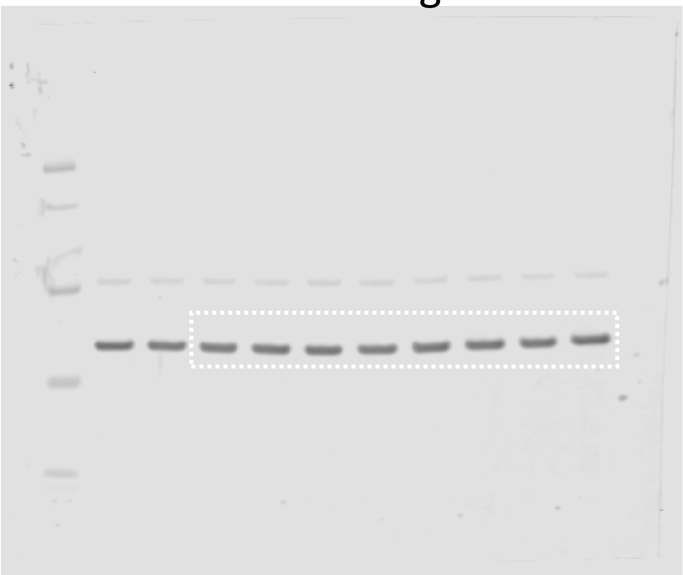

KAT8 IB: Extended Data Figure 11G

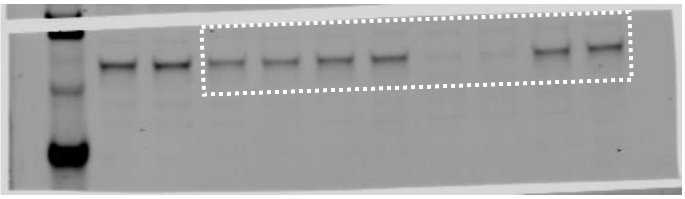

GAPDH IB for KAT8 IB: Extended Data Figure 11G

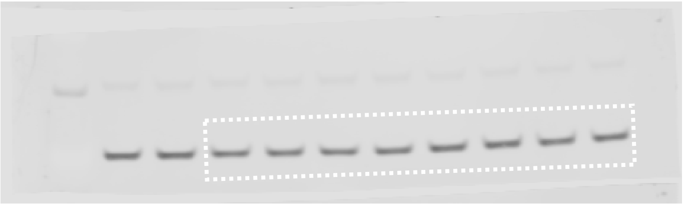

pUb(Ser65) IB: Extended Data Figure 15A

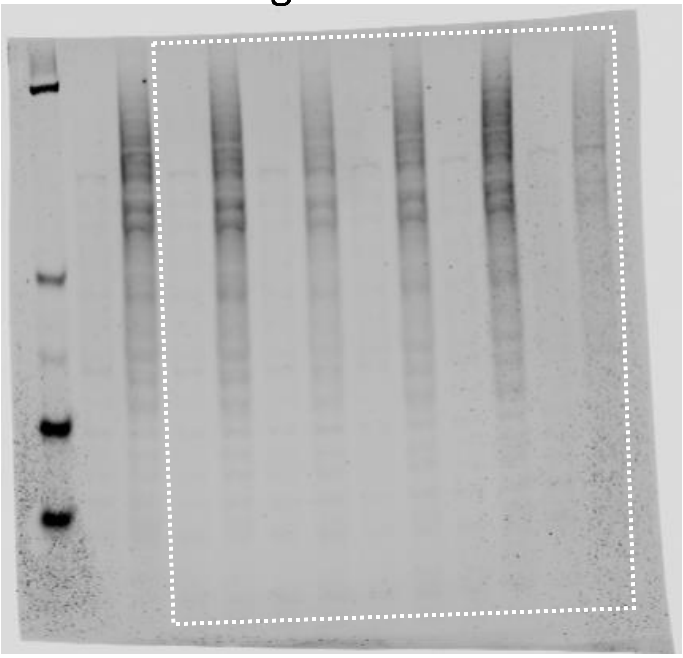

GAPDH IB for pUb(Ser65) IB: Extended Data Figure 15A

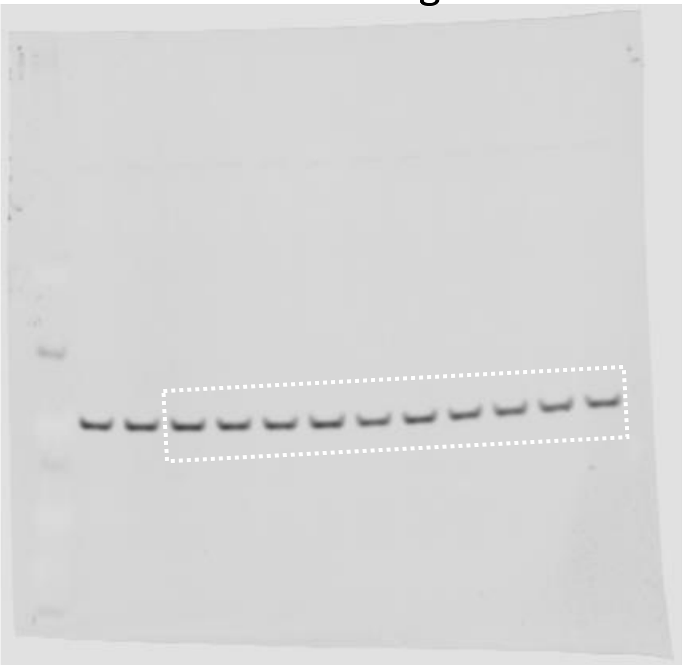

KAT8 IB: Extended Data Figure 15C

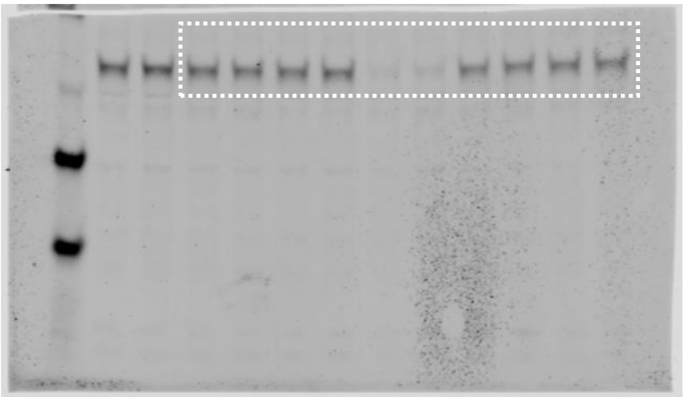

GAPDH IB for KAT8 IB: Extended Data Figure 15C

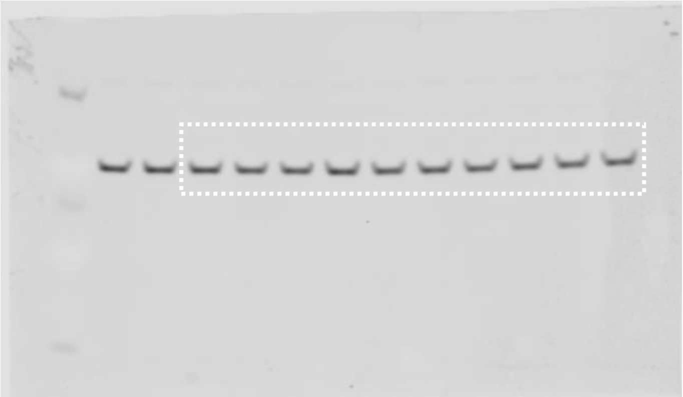

Tau IB: Extended Data Figure 15E

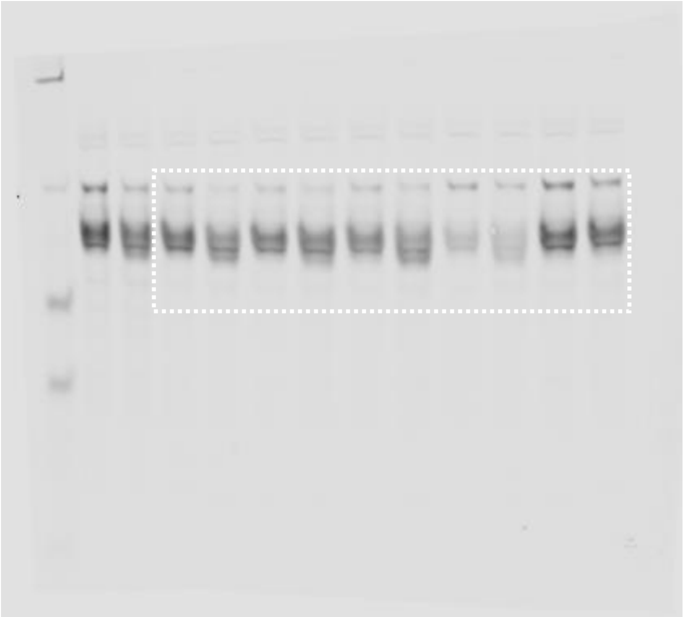

GAPDH IB for Tau IB: Extended Data Figure 15E

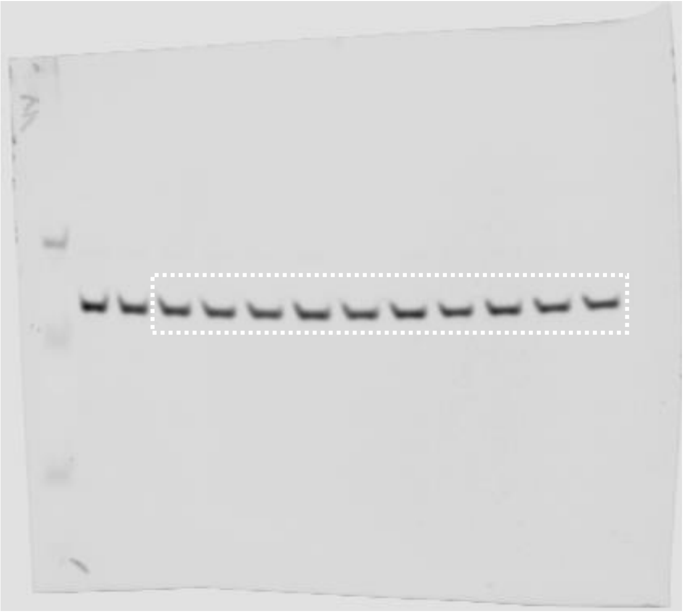

Supplement: awac325_Supplementary_Data [file awac325_supplementary_data.zip › brain-2021-02242-File012.pdf]
